# Agrobacterium-mediated transfer of the *Fusarium graminearum* Tri6 gene into barley using mature seed-derived shoot tips as explants

**DOI:** 10.1007/s00299-023-03129-z

**Published:** 2024-01-20

**Authors:** Dongying Gao, Sidrat Abdullah, Thomas Baldwin, Ann Caspersen, Edward Williams, Alvar Carlson, Mike Petersen, Gongshe Hu, Kathy Esvelt Klos, Phil Bregitzer

**Affiliations:** 1 Small Grains and Potato Germplasm Research Unit, USDA-ARS, Aberdeen, ID 83210 USA; 2 Oak Ridge Institute for Science and Education (ORISE) Research Participant, Small Grains and Potato Germplasm Research Unit, USDA-ARS, Aberdeen, ID 83210 USA; 3 Department of Plant Pathology, North Dakota State University, Fargo, ND 58108 USA; 4 Wisconsin Crop Innovation Center, University of Wisconsin-Madison, Middleton, WI 53562 USA

**Keywords:** Barley, Shoot tip transformation, Tri6, RNA interference, Somatic variation

## Abstract

**Key message:**

**We transferred the Tri6 gene into the elite barley GemCraft via new transformation method through shoot organogenesis and identified the rearrangements of transgenes and phenotypic variations in the transgenic plants**.

**Abstract:**

Despite its agronomic and economic importance, barley transformation is still very challenging for many elite varieties. In this study, we used direct shoot organogenesis to transform the elite barley cultivar GemCraft with the RNAi constructs containing *Tri6* gene of *Fusarium graminearum*, which causes fusarium head blight (FHB). We isolated 4432 shoot tips and co-cultured these explants with *Agrobacterium tumefaciens*. A total of 25 independent T0 transgenic plants were generated including 15 events for which transgene-specific PCR amplicons were observed. To further determine the presence of transgenes, the T1 progenies of all 15 T0 plants were analyzed, and the expected PCR products were obtained in 10 T1 lines. Droplet digital (dd) PCR analysis revealed various copy numbers of transgenes in the transgenic plants. We determined the insertion site of transgenes using long-read sequencing data and observed the rearrangements of transgenes. We found phenotypic variations in both T1 and T2 generation plants. FHB disease was evaluated under growth chamber conditions, but no significant differences in disease severity or deoxynivalenol accumulation were observed between two *Tri6* transgenic lines and the wildtype. Our results demonstrate the feasibility of the shoot tip transformation and may open the door for applying this system for genetic improvement and gene function research in other barley genotypes.

**Supplementary Information:**

The online version contains supplementary material available at 10.1007/s00299-023-03129-z.

## Introduction

Barley (*Hordeum vulgare,* 2n = 2x = 14) is an ancient crop that has been cultivated for about 10,000 years (Badr et al. 2000). Globally, about 70% of barley production is used for animal feed. However, barley is also an important food crop worldwide as it is rich in vitamins, minerals, and other chemical compounds, such as beta-glucans that are soluble fibers and can reduce the risk of cardiovascular diseases and obesity and improve metabolic syndrome (Pins and Kaur 2006; El Khoury et al. 2012). Barley is essential to the malting industry as its grains have all the enzymes required for converting starches into sugars. In the United States, malting barley accounts for over 60% of barley production. Barley is highly adapted to various climates, ranging from subarctic to subtropical regions, and has relatively shorter growth season that allows barley to grow and ripen in the colder and high-altitude areas where other cereal crops cannot grow well. Barley belongs to the grass family (Poaceae) which also contains bread wheat (*Triticum aestivum*, 2*n* = 6x = 42), oat (*Avena sativa,* 2n = 6x = 42), and other cereal crops. It is a diploid species that shares close evolutionary relationships with wheat and oat, although with a relatively smaller genome. For these reasons, barley has emerged as a model system for understanding cereal genome evolution and characterizing functional genes and metabolic pathways associated with important agronomic traits in polyploid cereals (Sato 2020).

Plant transformation is essential for crop improvement and a wide range of basic research such as understanding gene functions and precision gene editing (Lawrenson et al. 2015; Hinchliffe and Harwood 2019). Since the first transgenic barley was generated with microprojectile bombardment (Ritala et al. 1994; Wan and Lemaux 1994), several barley transformation systems have been developed, but the Agrobacterium-mediated transformation with immature embryo explants by somatic embryogenesis is the most widely used procedures for genetic transformation in barley as it can yield higher transformation efficiency (Tingay et al. 1997; Vyroubalová et al. 2011). However, this transformation system only works for limited barley genotypes and failed to generate transgenic plants for many elite barley cultivars. Mature seed-derived shoot tips, a region containing meristematic cells, have been demonstrated to be highly responsive to shoot organogenesis (Bregitzer et al. 2002) and have been successfully used for barley transformation in a patent application (Martinell et al. 2020).

Fusarium head blight (FHB) caused by *Fusarium graminearum* and other relevant species, is a devastating disease of wheat, barley, and other small grain crops (McMullen et al. 2012; Bai and Shaner 2004). This disease can result in yield loss and diminish grain quality due to shriveled kernels, lower test weight and contamination of deoxynivalenol (DON), a mycotoxin generated by *F. graminearum* (Salgado et al. 2011). As the mycotoxin can cause some serious problems for both human and animal health, such as harmful impacts to the immune systems (Fink-Gremmels 1999), grains with higher DON accumulation may result in lower market values and can even be completely rejected by the consumers. In addition, DON accumulation may promote the spread and development of FHB (Bai et al. 2002). Multiple management practices, including fungicide application, crop rotation and biological control, are used to prevent FHB outbreak and mitigate its losses (Salgado et al. 2011; McMullen et al. 2012). However, utilization of host resistance is the most efficient and environmentally friendly strategy to control this disease. Due to the lack of high-level FHB resistance resources, use of host resistance for improving barley FHB resistance remains as a big challenge, thus alternative methods, including transgenic approaches, may be necessary to address the FHB problem.

RNA interference (RNAi) is a molecular mechanism for gene silencing by which double-stranded RNA (dsRNA) targets the transcripts of specific genes and causes post-transcriptional repression. It is highly conserved across all eukaryotes and can trigger gene suppression for both endogenous genes and exogenous pathogenic sequences. Two general approaches of RNAi are widely used to protect plants, one is spray-induced gene silencing (SIGS) in which the dsRNA or sRNA derived from plant pathogens was directly sprayed onto plant leaves to suppress the expression of selective pathogenicity genes (Mitter et al. 2017). Another is host-induced gene silencing (HIGS) in which an inverted repeat that is homolog to functional genes of pathogens was integrated into plant genome to generate the small interfering RNA (siRNA) and induce the gene silencing of pathogens (Nunes and Dean 2012). HIGS is a transgenic modification method that offers a new and alternative strategy for plant disease management (Rosa et al. 2018). Thus far, RNAi has been used in many crops to reduce the losses caused by a wide variety of pests and pathogens such as fungi, bacteria, viruses, and nematodes (Baum et al. 2007; Cheng et al. 2015; Mitter et al. 2017; Worrall et al. 2019).

As DON is extremely important for promoting FHB spread and development and can severely impact the market value of the crop, suppression of DON production may provide another way to control FHB infection and reduce cereal seed contamination. Thus far, over 12 trichothecene genes have been identified to involve in the biosynthesis of DON in *F. graminearum* (Proctor et al. 2018). Among these genes, the *Tri6* gene serves as a global transcription regulator in the biosynthesis and transport of DON (Nasmith et al. 2011), and mutation of *Tri6* gene in *F. graminearum* significantly reduced its virulence and DON production in wheat (Baldwin et al. 2018). Therefore, the *Tri6* gene provides an excellent target to test the efficacy of RNAi technology for reduction of FHB infection and DON accumulation. To date, there are no reports in the literature of direct shoot tip transformation of barley, nor of a transgenic HIGS approach, targeting the *F*. *graminearum Tri6* for control of FHB and/or mitigating DON levels in barley.

## Materials and methods

### Plant materials

The malting barley cultivar GemCraft was developed by the barley and oat breeding program at the USDA-ARS Small Grains and Potato Germplasm Research Unit (SGPGR) and the seeds have been deposited to the U.S. National Plant Germplasm System (NPGS) with the accession number of PI 701910. The seeds of GemCraft were propagated in Aberdeen, Idaho, and used for barley transformation and other experiments in this study. In addition, the single-copy homozygous transgenic line TAGHG #20A generated by the barley genetics lab at SGPGR was also included.

### Assembly of plasmids encoding HIGS targeting of *F*. *graminearum Tri6*

Binary plasmids were generated in the Wisconsin Crop Innovation Center (WCIC) Molecular Technologies Department using synthetic parts (all produced by Synbiotech, Monmouth Junction, New Jersey) and Golden Gate cloning (Weber et al. 2011). A HIGS inducing transcriptional unit (TU) under control of the *Zea mays Ubiquitin 1* promoter (Christensen and Quail 1996) driving expression of an inverted repeat comprised of 602-basepair *F. graminearum Tri6* silencers (exhibiting over 99% sequence identity to the gene; accession no. LT222054:5,387,532–5,388,188) which were linked by 253-bp partial GFP sequence (accession no. AY056838) was designed. Polyadenylation of the HIGS cassette was under control of the *Agrobacterium tumefaciens* strain C58 *Nopaline synthase* (*NOS*) terminator (Bevan et al. 1983). The synthetic parts were used for *Bsa*IHFv2 (New England Biolabs, Ipswich, Massachusetts)-mediated Golden Gate Level 1 assembly of the *Tri6* HIGS cassette in the pL1R-3 Level 1 acceptor plasmid of the MoClo kit (pICH47822; Addgene plasmid # 48,009; Weber et al. 2011).

**Fig. 1:**
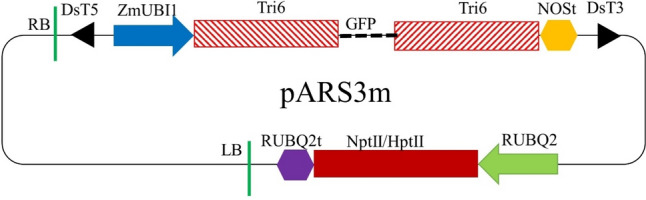
Schematic diagram of the vectors used for barley transformation. The two vectors, RC3947A and RC4625A, have all same constituents but selection marker gene. RC3947A contains the hygromycin phosphotransferase-II (HptII) gene whereas RC4625A has the neomycin phosphotransferase II (NptII) gene. The right border (RB) and the left border (LB) of the vector are marked by the vertical green lines (Colour figure online)

Plant selectable marker TU was assembled using for expression and polyadenylation the promoter and terminator from the *Oryza sativa* polyubiquitin gene (*RUBQ2*) (Wang et al. 2000). To facilitate selection of transgenic barley on media containing Hygromycin B, the *hygromycin* *phosphotransferase II* (*HptII*) gene (Waldron et al. 1985) was deployed. In separate experiments, which examined selection of transgenic barley on media containing G418 Sulfate, the selection marker was engineered to contain a copy of the *neomycin phosphotransferase II* (*NptII*) (Vancanneyt et al. 1990) gene which was synthesized to include the *Solanum tuberosum LS1* intron (Eckes et al. 1986) to ensure that the cassette would operate solely in eukaryotes. All plant selectable marker TU were assembled by *Bsa*IHFv2 mediated Level 1 Golden Gate reactions in the pL1R-1 Level 1 plasmid of the MoClo kit (pICH47802; Addgene plasmid # 48,007; Weber et al. 2011), ensuring that the direction of the transcription would be towards the T-DNA Left Border (Fig. 1).

Both constructs were engineered to allow for post-transformation migration of the HIGS cassette away from the plant selection marker to ultimately produce plant selection marker-free transgenic lines in our further work. Synthetic fragments of the inverted tandem repeats (TIR) of the maize Ds transposon (Upadhyaya et al. 2006) were installed into pL1R-2 (pICH47811; Addgene plasmid # 48,008; Weber et al. 2011) and pL1R-4 (pICH47831; Addgene plasmid # 48,010; Weber et al. 2011), ensuring in the binary construct that these elements would correctly flank the *Tri6* transgene inverted repeat cassette and facilitate transposition away from the plant selectable marker.

The aforementioned Level 1 TU were utilized in *Bbs*IHF (New England Biolabs) mediated Level 2 Golden Gate assembly in the MoClo kit (Weber et al. 2011) RK2 binary backbone pAGM4673 (Addgene plasmid # 48,014; Weber et al. 2011) to produce the final binary plasmids RC3947A (the *HptII* containing construct, produced by Golden Gate reaction number RCGG2583) and RC4625A (the *StLS1* intronized *NptII* containing construct, produced by Golden Gate reaction number RCGG3555) (Fig. 1).

Plasmids RC3947A and RC4625A were used to electroporate (McCormac et al. 1998) clones of *Agrobacterium tumefaciens* strain AGL-1, previously loaded with the helper plasmid pPHP71539 (Anand et al. 2018)) to produce the ternary vector *Agrobacterium* stocks WCIC-A-00426 and WCIC-A-00607, respectively.

### Barley transformation

GemCraft is a hulled barley variety for which the outer husks tightly attach to the whole grains. To obtain the explants for transformation, the hulls of mature seeds were manually removed. The naked seeds were sanitized in 20% Clorox solution for 5 min, and excess bleach removed with 10-min duration rinsing the explants with sterilized water three times. The cleaned seeds were subsequently dried in laminar flow hood to reduce the internal moisture content. The shoot tips were isolated and co-cultured with the AGL-1 strain of *Agrobacterium tumefaciens*, containing the ternary helper plasmid pPHP71539 and one of the aforementioned binary vectors, for about 1 h including 30 min incubation during centrifugation (H6000A Sorval rotor at 5000 RPM) and 30 min afterwards. The explants (not rinsed) were grown on the induction media with a high relative amount of cytokinin to auxin for a total of 2 weeks at 27 °C and photoperiod of 16 h light and 8 h dark to induce multiple buds/shoots. After the 2-week bud induction period, the barley explants were transferred to plant growth regulator free regeneration media, supplemented with the appropriate selective chemicals, then cultured at 27 °C and photoperiod of 16 h light and 8 h dark. Explants were transferred every 3–4 weeks as needed on fresh regeneration media with selection to prevent overgrowth of *Agrobacterium tumefaciens*. The T0 generation plants were transferred into soil and grown in the greenhouse at the Wisconsin Crop Innovation Center to collect the leaf tissues for PCR analysis and to produce seeds. All media used in this study are listed in Table 1.

**Table 1:** The list of the media used for barley transformation

Ingredients and Notes	Amount to add per liter
Inoculum resuspension and co-culture medium	
Phytotechnology Laboratories B5 salts G398	1.284 g
Glucose	30 g
MES hydrate (Alfa Aesar CAS 4432-31-9)	2.8 g
Note: PH 5.4	
Bud induction medium	
Phytotechnology Laboratories B5 salts G398	3.21 g
Maltose	30 g
L-Proline (Caisson Labs CAS 147-85-3)	1 g
Casein hydrolysate	1 g
Cupric Sulfate (CuSO4) (1 mg/ml)	1.25 ml
MES	2 g
Cleary’s	0.06 g
Phytagel	3.5 g
Carbenicillin (100 mg/ml stock)	2 ml
Cefotaximine (100 mg/ml stock)	2 ml
Timetin (150 mg/ml stock)	1 ml
Thidiazuron (TDZ) (1 mg/ml stock)	10 ml
2,4-D (1 mg/ml stock)	0.5 ml
Note: PH 5.8	
Regeneration medium	
Phytotechnology Laboratories B5 salts G398	3.21 g
Maltose	30 g
Cupric Sulfate (CuSO4) (1 mg/ml)	1.25 ml
Cleary’s	0.06 g
Phytagel	3.5 g
Carbenicillin (100 mg/ml stock)	2 ml
Cefotaximine (100 mg/ml stock)	2 ml
Timetin (150 mg/ml stock)	1 ml
G418 (30 mg/ml stock)	2 ml
Note: PH 5.8	

### PCR analysis

Two pairs of PCR primers were used to detect the presence of transgenes including D0037 (5′-GAAAAAGCCTGAACTCACCG-3′) and D0038 (5′-CATATCCACGCCCTCCTAC-3′) for amplifying a region of *HptII* gene (Collier et al. 2017); D0145 (5′-ATACGCTTGATCCGGCTAC-3′) and D0146 (5′- GTAGAAGCAGAAACTTACCGG-3′) for the *StLS1* intronized *NptII* gene. PCR analysis for T0 plants was conducted at WCIC using the RedExtract-N-Amp Plant PCR kit (Sigma-Aldrich, Burlington, MA), and was run on 1.5% agarose gel in 1X SB buffer and stained with the SYBR Safe DNA gel stain (Thermo Fisher Scientific, Waltham, MA). PCR detection for T1–T3 transgenic plants was carried out at SGPGR by following our previous protocol (Gao et al. 2022). Amplifications were conducted in a Bio-Rad S1000 Thermal Cycler in 20 μl reactions consisting of ~ 50 ng of genomic DNA, 0.2 mM primer, deionized water, and 10 μl EconoTaq PLUS GREEN 2X Master Mix (LGC Biosearch Technologies, Middleton, WI) containing 0.1 units/μl of EconoTaq DNA Polymerase, Reaction Buffer (pH 9.0), 400 μM dATP, 400 μM dGTP, 400 μM dCTP, 400 μM dATP, and 3 mM MgCl2. The PCR temperature cycling conditions were 1 cycle of 98 °C for 2 min; 35 cycles of 95 °C for 30 s, 55 °C for 30 s, 72 °C for 30 s; and 1 cycle of 72 °C for 5 min. Amplification products were run on 0.8% agarose gels and stained with ethidium bromide.

### ddPCR analysis

Transgene copy number enumeration via droplet digital PCR was conducted following the method as described previously (Collier et al. 2017). Young leaves of 16 T1 and 175 T2 transgenic plants, wild type and the TAGHG #20A (single-copy check) which was validated by PCR for four generations were used to extract genomic DNA. 4 ug of barley genomic DNA for each sample was digested with the HinfI restriction enzyme (New England Biolabs, Ipswich, MA) at 37 °C for 1 h. The Master mixture was prepared with the ddPCR Supermix for Probes (No dUTP) (Bio-Rad Laboratories, Hercules, CA) by following the manufacturer’s protocol, the mixture (23 μl) contains 5 uM FAM probe (reference gene, Actin 1 (Gines et al. 2018) Probe: 5′-[6-FAM]TGTTTGAGACTTTCAATGTTCCTGCC[BHQ1a-Q]-3′), 18uM reference gene Primer F (Actin F1: 5′ CCCAAAAGCCAACAGAGAGA -3′), 18 uM reference gene Primer R (Actin R1: 5′- GCCTGAATAGCGACGTACAT-3′), 5uM HEX probe (transgene, NOS P3: 5′-[HEX]ACAAAATATAGCGCGCAAACTAGGA[BHQ1a-Q]-3′), 18uM transgene (for detection of the *NOS* terminator included in the HIGS inducing *Tri6* RNAi cassette) Primer F (NOS F3: 5′- AGAGTCCCGCAATTATACATT-3′) and 18 uM transgene Primer R (NOS R3: 5′- TAACATAGATGACACCGCGC-3′). The reaction mixture (25 μl) containing 250 ng digested template DNA and 22.5 μl Master mixture was added to a new 96-well PCR plate. PCR was conducted with a C1000 Touch™ Thermal Cycler ((Bio-Rad Laboratories, Hercules, CA) under the following conditions: enzyme activation at 95 °C for 10 min, amplification of 40 cycles (94 °C for 30 s denaturation, 54 °C for 1 min annealing/extension), and enzyme deactivation at 98 °C for 10 min. Two independent reactions were run for each sample, and the ddPCR results were analyzed using the Bio-Rad QuantaSoft™ Analysis Pro software. Each of the two reactions was separately analyzed and their average represents the copy numbers of the reference gene and transgene.

### FHB evaluation

The *F. graminearum* strain PH-1 (NRRR: 31,084) was used to prepare the conidial suspension as previously described (Hao et al. 2019). The fresh spores were multiplied in mung bean broth using a previously produced stock of PH-1 (5  × 10^6^ conidia/ml) maintained in the −80 °C freezer. Each 250 ml flask was inoculated with 10–20 ul of the PH-1 strain in 100 ml mung bean broth, and the culture was incubated for 21 days at 28 °C in the dark with a rotary shaker set at 125 rpm. Conidia were isolated by centrifuging the culture at 3000 × g for 20 min after filtering the culture through Miracloth (Millipore-Sigma, Burlington, MA). The recovered conidia were then resuspended in 5 ml sterile deionized water. The conidial concentration was measured using a hemocytometer and diluted to 5  × 10^4^ conidia/mL. Tween-20 (0.04%) was added to improve conidial adhesion and dispersal (Hallen-Adams et al. 2011).

The seeds of two T3-generation transgenic lines and the wildtype (WT) GermCraft were planted in 4-inch square pots (800 mL volume) and the plants were maintained in the growth chambers and the greenhouses at the USDA-ARS Small Grains and Potato Germplasm Research Unit. For the growth chamber, fluorescent and incandescent lights were used to maintain a 13-h photoperiod with the light level of 54–90 μmol/m2/s at the temperature sets: 1 h at 16 °C, 1 h at 18 °C, 10 h at 20 °C, 1 h at 18 °C, and 11-h dark period at 16 °C. In the greenhouse, the light was maintained on a 14-h day cycle, with additional LED lights used to maintain light saturation during cloudy days. The greenhouse temperature ranged from 21 °C to 25 °C during the day and 18.5 °C to 20 °C at night. At roughly 8 weeks after sowing plants on which heads fully appeared were inoculated with the spore suspension using a G-R model 15 atomizer (G-R Electric Manufacturing Co LLC, Manhattan, KS). The inoculated plants were allowed to dry for 30 min and then moved into a dew chamber for 24 h. The dew chamber was kept at or near 100% relative humidity and operated at 25 °C with a 12-h photoperiod at a light intensity of 250 μmol/m2/s. The plants were removed from the dew chamber after an overnight incubation period and the inoculated heads of each pot were covered with plastic bags that were fastened with twist ties to produce a humid environment for 3 days and moved into the growth chamber with the same settings as previously described. After 3 days, the plastic bag was removed, a glassine bag was used and placed back inside the growth chamber. Heads of each pot were harvested separately and scored for severity on 21 days after inoculation (DAI). A total 17 individuals for each genotype including transgenic lines and the wild type were analyzed. The heads from same plant were mixed, freeze dried, ground and sent to the North Dakota State University Barley DON Testing Lab for measuring the DON accumulation in the grains by combining Gas chromatography-electron capture detector (GC-ECD) and Gas chromatography–mass spectrometry (GC–MS) (Tacke and Casper 1996; Jin et al. 2018). The disease severity of FHB was estimated by the ratio of fusarium-infected kernels to the total kernels (the number of infected kernel/Total number of kernels *100%) at the 21 days after inoculation. The disease severities and DON concentrations of 17 individuals for each genotype were used for *T*-test.

## Results

### Generation of transgenic plants with barley shoot tips

**Fig. 2:**
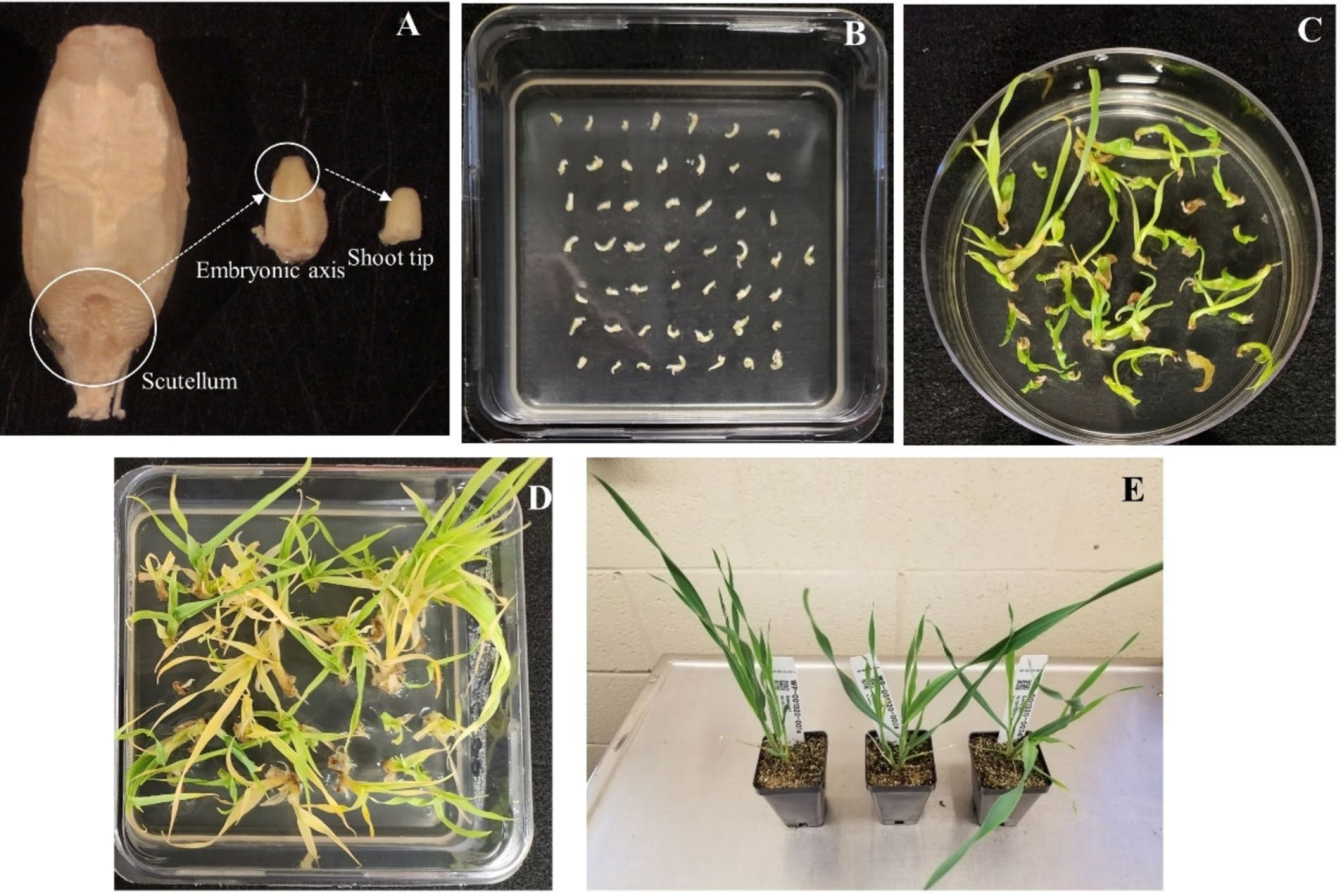
The major procedures of barley shoot tip transformation.** A** collection of barley shoot tips; **B** shoot tip explants after co-culture; **C** explants plated for bud growth and amplification; **D** explants after 3 weeks on selection media for root regeneration; **F** The first rooted events (T0 plants) in soil

The protocol widely used for barley transformation was established using the immature embryos of the spring type cultivar Golden Promise as the explants (Tingay et al. 1997). However, this approach has yielded very low transformation frequency or failed to generate transgenic plants for many elite cultivars even with the optimized protocols (Hensel et al. 2008; Harwood 2014; Lim et al. 2018). We applied a new barley transformation system which used the barley shoot tips (the main part of the embryonic axis was removed) as the explants by following the patent (Martinell et al. 2020) which transferred GUS and other reporter gene into the spring malting barley cultivar GemCraft. Two vectors, RC3947A and RC4625A, were constructed and used for transforming the shoot tips of GemCraft. Except for the selectable marker genes, both constructs have same HIGS inducing hairpin loop of *Tri6* gene segments from the *F. graminearum* fungus, promoters, terminators, and other components (Fig. 1). RC3947A carries the *HptII* gene whereas RC4625A contains the *StLS1* intron containing *NptII* gene. A ternary vector approach was used for co-cultivation of a total of 4,432 shoot tips isolated from the mature seeds of GemCraft (Fig. 2A), with *Agrobacterium tumefaciens* strain AGL-1 which carried both a helper plasmid and either the *HptII* (Agrobacterium stock WCIC-A-00426) or *NptII*-intron (Agrobacterium stock WCIC-A-00607) versions of the *Tri6* HIGS vectors. After a 4-day co-culture, shoot tips were transferred to selection-free bud induction medium with a high level of thiadiazuron (TDZ) to amplify potentially transformed cells and produce new shoots/buds (Fig. 2B, C). After 2 weeks on bud induction medium, shoot tips were transferred to either hygromycin or G418 sulfate containing selection media to select, recover and develop shoots and roots (Fig. 2D). In total, 25 transgenic barley events, rooted on selection media, were subsequently transplanted to soil, after which two died in the nursery (Table 2). The surviving 23 T0 plants, generated either from WCIC-A-00426 (containing plasmid RC3947A; assigned the number WP001320 or 1320 for short), or WCIC-A-00607 (containing construct RC4625A; assigned the number WP001337 or 1337 for short) were maintained in the greenhouse through sexual reproduction to produce transgenic T1 seeds (Fig. 2E).

**Table 2:** Summary of GemCraft shoot tip transformation

Construct	WCIC-A-426 (contains the plasmid RC3947A) (HptII /hyg)	WCIC-A-607 (contains the plasmid RC4625A) (NptII /G418)
No. of inoculated shoot tips	2478	1954
No. of T0 plants*	8	17
Transformation frequency (%)	0.20	0.51
T0 plants with less chimerism (Transformation frequency, %)	1 (0.04)	6 (0.31)
T0 plants with more chimerism (Transformation frequency, %)	4 (0.16)	4 (0.20)
Escaped T0 plants	2	6
Dead T0 plant in nursery	1	1

*Rooting on selection medium

### Molecular analysis of transgenic plants

To determine if the transgenes were integrated into the barley genome, eight leaves from each of the 23 T0 plants were collected and used for PCR analysis with the primers specifically amplifying the plant selection marker genes. Fifteen plants were confirmed to contain the selection marker genes including seven plants that had a less chimeric probability (LCP) for which at least five leaves generated the selection marker specific PCR amplicons. Additionally, specific amplifications were detected from one to three leaves of another eight T0 plants suggesting they had a more chimeric probability (MCP) for which less than 50% of the tested leaves (or four leaves) were PCR positive. No amplifications were found for all eight leaves of the remaining eight T0 plants (Table 2) implying that they may have escaped from the selection during the transformation process. It seemed that the vector with *NptII* gene showed higher transformation frequency (0.51%) and generated more LCP plants (0.20%) that the *HptII* system, but more experiments are needed to compare the selected efficiency of the two systems. The 15 potential transgenic plants with PCR amplifications were reared to maturity in the greenhouse, and all these plants were fully fertile and produced sufficient T1 seeds ranging from 31.3 g to 109.2 g for our next experiments.

To further confirm the presence of transgenes and test if they can be passed to the next generation, a total of 426 T1 seeds from the 15 T0 individuals were planted in the greenhouse at the ARS research unit in Aberdeen, ID, of which 324 seeds germinated and developed into plants. DNA was extracted from 323 T1 plants and used for PCR analysis (Table 3). Specific PCR products were found in the progeny of all seven LCP T0 plants, indicating the transgenes were inserted into the genome of GemCraft and can be passed to the T1 generation. In addition, we also detected specific amplifications in the T1 plants derived from six MCP T0 plants suggesting the transgenes may also have integrated into the barley genome. No specific PCR amplification was detected in the progeny of two MCP T0 plants, WP001337-5 and WP001337-12, so more T1 plants are needed for testing the presence/absence of the transgenes in those two events. Segregation of transgenes were observed for all 13 T1 lines with specific amplicons suggesting the transgenes were inserted into the barley genome before the emergence of gametes. It should be noted that some T1 plants may lost the selective marker by segregation but still maintained the Tri6 silencer, these plants cannot be detected by our PCR analysis as we only used the primers specifically targeting the selection marker genes.

**Table 3:** Summary of PCR analysis in T0 and T1 plants

Line name	Selection marker	PCR for T0 plants (%)**	PCR for T1 plants						
			Planted seeds	Germinated seeds	Germination rate (%)	Plant number for PCR	Plant number with amplification	Plant number without amplification	Fraction of amplification (%)
001320-1	hptII	3/8 = 38%	51	35	70.0	35	3	32	8.6
001320-2	hptII	1/8 = 13%	22	19	86.4	19	6	13	31.6
001320-4	hptII	3/8 = 38%	20	19	95.0	19	6	13	31.6
001320-6	hptII	3/8 = 38%	21	19	90.5	18	1	17	5.6
001320-7*	hptII	7/8 = 88%	26	19	73.1	19	15	4	78.9
Total			140	111	79.3	110	31	79	28.2
001337-2*	nptII	8/8 = 100%	36	29	80.1	29	27	2	93.1
001337-3	nptII	1/8 = 13%	52	44	84.6	44	2	42	4.5
001337-4*	nptII	8/8 = 100%	21	20	95.2	20	12	8	60
001337-5	nptII	2/8 = 25%	21	18	85.7	18	0	18	0
001337-8*	nptII	5/8 = 63%	21	20	95.2	20	17	3	85.0
001337-11*	nptII	5/8 = 63%	26	18	69.2	18	14	4	77.8
001337-12	nptII	3/8 = 38%	23	16	69.6	16	0	16	0
001337-15	nptII	2/8 = 25%	23	13	56.5	13	1	12	7.7
001337-16*	nptII	7/8 = 88%	30	17	56.7	17	11	6	64.7
001337-17*	nptII	5/8 = 63%	33	18	54.5	18	8	10	44.4
Total			286	213	74.5	213	92	121	43.2

* mean the T0 plants with low chimeric probability. ** the fraction means the number of leaves with PCR amplification to the total collected leaves (8 leaves from each plant)

To enumerate transgene copy number, the wildtype (WT) GemCraft, and 13 T1 plants derived from nine different transgenic events were used to conduct droplet digital PCR (ddPCR) analysis. Furthermore, we also used TAGHG#20 as a control, because it contains a single-copy homozygous *NOS* sequence (Ann Caspersen, unpublished data). No transgene was detected in the WT and four T1 plants which was consistent with our endpoint PCR results as no expected amplification, using the marker gene primers, were observed from any of these four plants. One copy of the transgene was detected in six T1 plants suggesting that they are single copy hemizygous for the transgene. Two copies of transgene were detected in TAGHG#20 and three T1 plants. Interestingly, three T1 plants contained 3–7 copies of the transgene (Figure S1). To gain more clues about the copy number variations in the transgenic plants, we conducted ddPCR for 174 T2 individuals from 16 different T1 plants, the transgene copy number measured from these T2 plants ranged from 0 to 15 (Table S1). All tested T2 plants from five T1 plants, including 1320-002a-2, 1320-002a-3, 1320-007a-3, 1337-015a-3 and 1337-017a-6, contained two copies of the transgene implying that these plants are either single copy homozygous or hemizygous and have one transgene copy at each of the two closely linked loci. To gain more clues about the genetic stability of the transgenes, two T3 lines named 1320-002a-2-2 and 1337-015a-3-8 were used for further molecular analysis as they generated more seeds for downstream studies. For each T3 line, 16 plants were tested, and specific PCR amplifications were observed in all plants. Eight individuals for each T3 line were also used for ddPCR assay and two copies of the transgene was detected in all tested plants. Thus, our PCR and ddPCR analysis suggested that both 1320-002a-2-2 and 1337-015a-3-8 are two stable T3 transgenic lines.

### Determination of the integration site of transgenes using long-read sequencing data

**Fig. 3:**
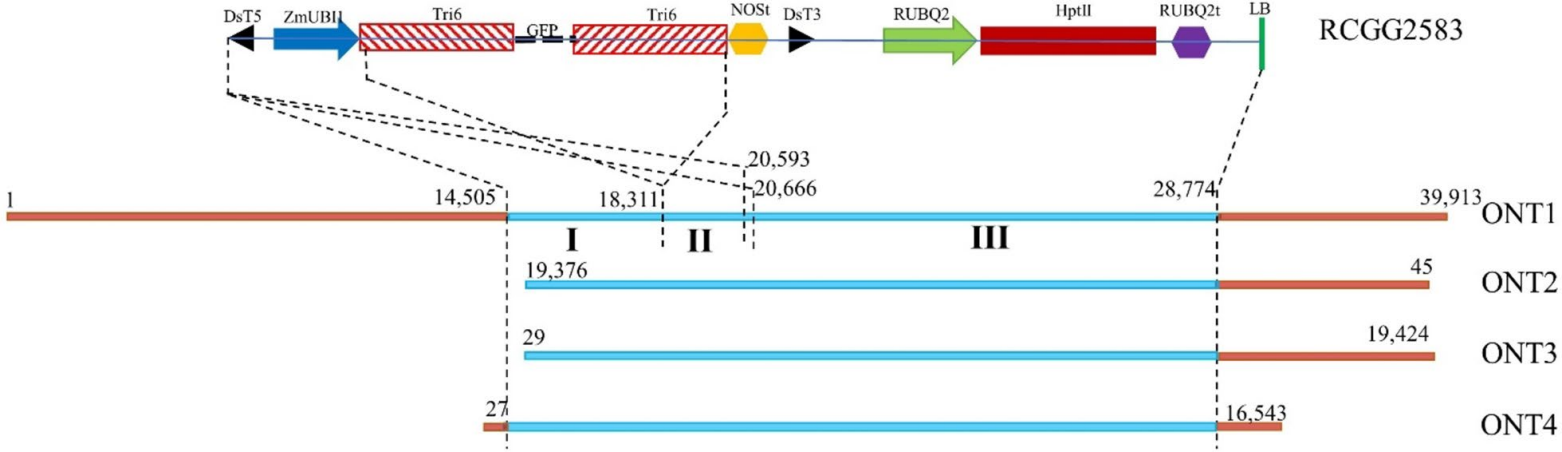
Comparison between RC3947A vector sequences and four long sequencing reads. Above is the vector sequence, the transgenes are marked as that in Fig. 1. Other sequences between the right border and the left border of the vector have been eliminated during the integration. Button is the four long reads which contain both vector sequences (blue) and the barley genomic sequences (brown). The alignment regions are indicated by the broken lines (Colour figure online)

To define the integration site of transgenes, we sequenced the genome of the T3 line 1320-002a-2-2 using the Nanopore GridION sequencer. A total of 531,066 reads covering 10,481,718,912-bp (10.5 Gb or 2 times of the barley genome) sequencing data were generated, and the average read length was 19,736.1 bp. We further conducted BLASTN search against the 10.5-Gb long read data using the RC3947A vector sequence as the query, and 194 significant hits (*E*-value < 1 × e^−10^) were found. Among these hits, 190 were ignored as their alignments were short (< 230-bp). However, four reads named ONT1 to ONT4, which sizes range from 16,548 bp to 39,913 bp, showed sequence highly identity with the vector sequence (*E*-value = 0) over alignment lengths of 7000 bp. Among them, ONT1 and ONT4 have barley genomic sequences that surround the vector sequence on both sides whereas ONT2 and ONT3 only contain the vector sequence and barley sequence on one flanking region (Fig. 3). Both flanking sequences of ONT1 and ONT4 were used to search against the barley reference genome (MorexV3_pseudomolecules_assembly) (Mascher et al. 2017), and the sequence alignments indicated that the transgenes were likely inserted into the site that is correspondent to 564,792,216 bp on Chromosome 5 of the reference barley genome. Impressively, our sequence comparisons also revealed the rearrangements of transgenic sequences during their integration. The pairwise sequence alignments between ONT1 and the RCGG2583 vector sequence can be divided into three blocks. The block I spanned 14,505 bp to 18,311 bp of ONT1 and matched the vector sequence covering DsT5 to the inverted Tri6 sequences. The block II located from 18,311 bp to 20,599 bp of ONT1 showed significant sequence identical to the vector sequence containing the DsT5, maize polyubiquitin-1 (ZmUBI1) promoter and partial Tri6 sequence. However, the block II exhibited opposite orientation with the block I. The 7.9-bp block III (20,666 bp to 28,774 bp of ONT1) showed same orientation with the block I and contained the entire set of transgenes spanning from DsT5 to the left boundary (LB) sequence (Fig. 3). It should be noted that the rearrangements of vector sequence were also identified in ONT2, ONT3, and ONT4 as they showed similar alignments with that of ONT1.

### Evaluation of FHB resistance in transgenic barley plants

**Fig. 4:**
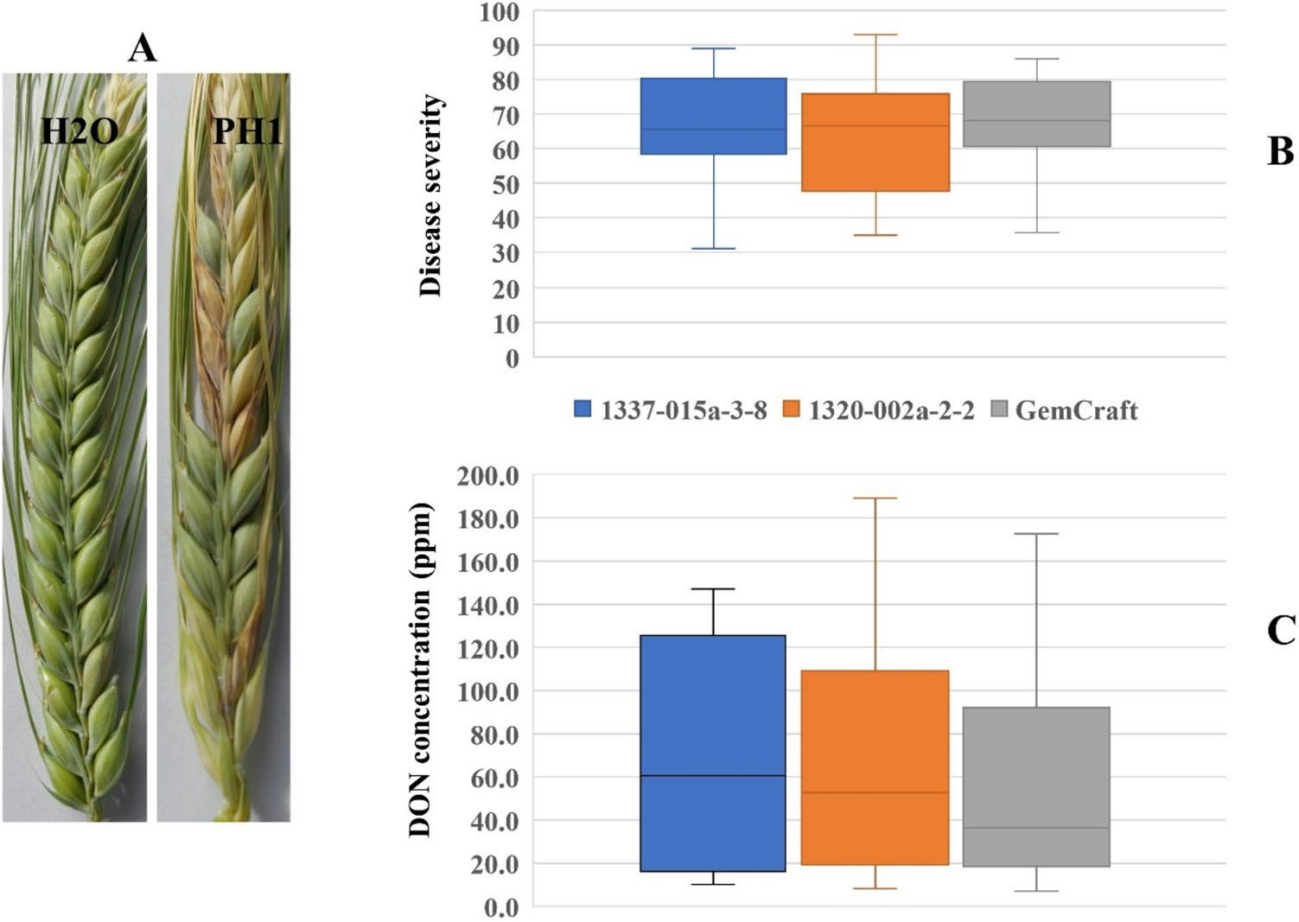
Evaluation of FHB resistance in two transgenic lines and the wild type (WT). **A** Spike inoculated with water (left) and *F. graminearum* (right). The infected kernels were shriveled and bleached. **B** Disease severity of two transgenic lines and the WT;** C** DON accumulation of two transgenic lines and the WT

To investigate whether HIGS targeting the *Tri6* gene can impact the resistance to FHB, two T3 transgenic lines, 1320-002a-2-2 and 1337-015a-3-8, and the WT GemCraft were infected with the *F. graminearum* strain PH-1 and maintained under controlled conditions for disease development. The plants inoculated with water (control) showed no FHB symptoms and all kernels were green (Fig. 4A). All inoculated spikes developed symptoms of FHB infection, such as shriveled and discolored kernels in both transgenic lines and the WT (Fig. 4A), suggesting the conditions were favorable for fungal growth and disease development. However, the percentages of fusarium-infected kernels (FIK) to total kernels greatly varied between different spikes within the genotypes (Fig. 4B). For the WT plants, the rates of FIK ranged from 36 to 86. The average FIK rates of 1320-002a-2-2, 1337-015a-3-8, and the WT were 64, 66 and 68, but no statistically significant difference was detected between transgenic lines and the WT (*T*-test, *P* > 0.05).

We next harvested the grains from both transgenic and WT plants and measured the DON accumulation to test if the HIGS targeting *Tri6* transgenic plants generated lower levels of DON than the WT. No DON contamination was found for the plants inoculated with water, but DON was detected in all plants infected by the *F. graminearum* fungus. Dramatic differences in DON concentrations were observed within all three genotypes (Fig. 4C). For the WT, the DON ranged from 7.1 ppm to 172,6 ppm between plants. The average DON accumulation in 1320-002a-2-2, 1337-015a-3-8, and the WT GemCraft was 71.6, 69.9 and 60.8, respectively. Two transgenic lines seemed to accumulate higher DON, but the differences were not statistically significant between three genotypes (*T*-test, *P* > 0.05).

### Morphological variations

**Fig. 5:**
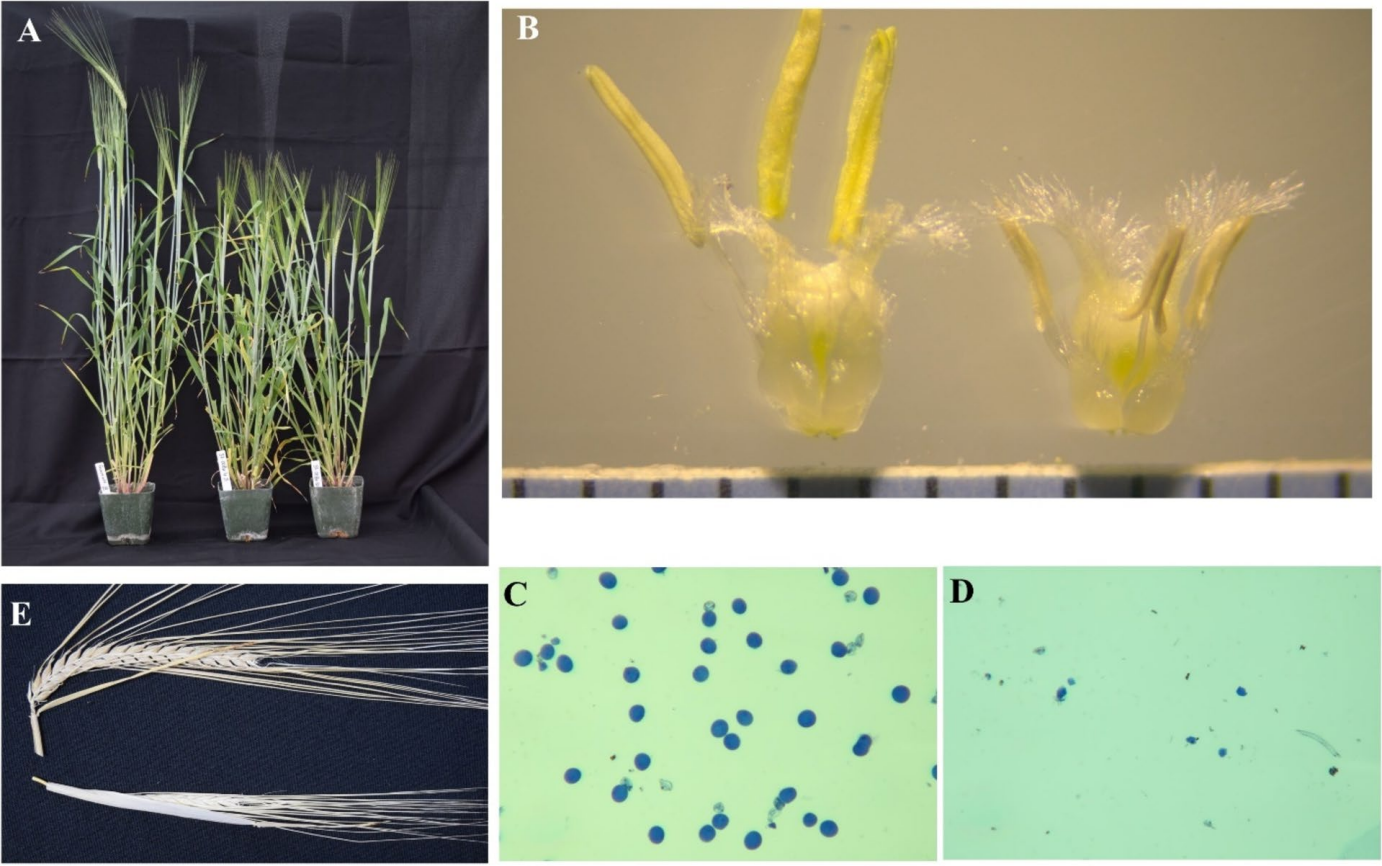
Phenotypic variation of the T2 transgenic line 1337-008a-6.** A** The WT (left) and two transgenic plants (right). **B** The anthers and ovule of WT (left) and transgenic plant (right). **C** The staining pollens of WT; **D** The staining pollens of transgenic plant; **E** The mature spike of WT (above) and transgenic plant (below), many spikes of the transgenic plants cannot fully extend out the leaf auricle

Previous studies revealed that transformation frequently produces plants containing genetic and morphological changes called somaclonal variation (Bregitzer et al. 2002; Coronel et al. 2018). During harvest of the transgenic plants, we found two T1 individuals which showed visible phenotypic variations. One individual, 1337-003a-6, generated no seeds (Figure S2A), whilst another plant, 1320-002a-5, exhibited angled bends in the internodes and fewer tillers, relative to WT (Figure S2B). To determine if the observed somatic variation is transmitted to the next generation, the seeds from 1320-002a-5 and other 17 T1 plants were planted on April 16, 2022. The T2 progenies of 1320-002a-5 showed distinct phenotypes, some plants exhibited angled bends in the internodes of some tillers (Figure S2C) whereas others developed a main stem but no or few tillers (Figure S2D, E). Two T2 progeny from the T1 plant 1337-008a-6 only amplified tillers and grew leaves but they did not flower and produce seeds (Figure S2F). To further confirm the morphological changes, we replanted the T2 seeds of 1337-008a-6 plant on January 25, 2023. All 10 T2 progeny of 1337-008a-6 showed similar phenotypic traits, they produced flowers, but were shorter than the WT GemCraft (Fig. 5A). The T2 plants also developed smaller and shriveled anthers compared to the WT plants (Fig. 5B). We further investigated the viability of mature pollen grains with 2% acetocarmine solution and observed a high number of stained pollens in the WT plants (Fig. 5C). However, the T2 plants of 1337-008a-6 seemed to develop very limited number of pollen and all the pollen we checked were smaller and abnormal (Fig. 5D). The heads (ears or spikes) of WT plants exhibited higher seed setting rate (> 98%) whereas most of the heads from the T2 plants were completely sterile (Fig. 5E). Additionally, four T2 progeny of the 1337-002a-3 T1 plant cannot head and flower in late August 2022 (Figure S2G). However, we replanted the T2 seeds of 1337-002a-3 in January 2023 and found that all the T2 plants generated flowers and mature seeds in May 2023, and they showed similar phenotypes with the WT (Figure S2H).

## Discussion

### Barley shoot tip transformation

Transformation of many major cereal crops including barley is still challenging. So far, immature embryos are widely used as the explants for cereal transformation (Sticklen and Orabya 2005; Harwood 2014; Hayta et al. 2019). However, this method requires to grow plants for manually dissecting immature embryos of the correct stage. Additionally, the entire process from immature embryo dissection to callus formation and finally rearing of transgenic plants in soil to produce transgenic T1 seeds can take 7–12 months or longer. The isolation of immature embryos is time-consuming and requires skilled hands. Additionally, the transformation systems with immature embryos only worked well with some specific genotypes and were inefficient for many elite cultivars. In this study, we conducted barley shoot tip transformation and obtained plantlets in 10 weeks after initial explant inoculation which significantly shortens the amount of time to produce T1 seeds. Also, the shoot tip system simplifies the culture steps and saves labor. Theoretically, shoot tip transformation systems are less genotype-dependent and some preliminary results also supported this hypothesis (Schrammeijer et al. 1990; Sticklen and Orabya 2005; Chen et al. 2014). Therefore, the shoot tip transformation system offers an alternative method for the transformation of barley and other cereal crops. The transformation frequency of the immature embryo-based systems usually ranged from 1 to 5% and has even reached 25% for certain cereal genotypes (Hayta et al. 2019). However, our barley transformation frequency was 0.34% (15/4,432*100) which was much lower than that of routine transformation systems in cereal crops but it is similar to that observed in wheat shoot tip transformation (~ 0.4%) (Ye et al. 2023).

It is necessary to optimize the barley shoot tip transformation system for improving the transformation efficiency and reducing the rate of production of chimeric plants. In this study, we conducted PCR analysis to identify the transgenes and the chimeras with the young leaves of transgenic seedlings. However, more experiments including Southern blot are needed to estimate the chimeric rate of shoot tip transformation for all tillers of each regenerated plants. Chimeras are always a major problem in shoot tip transformation as they can reduce the frequency of real transformation and cause extra works for identifying and eliminating the unfavorable plants. In most cases, chimerical plants were likely derived from the multiple cells or non-transgenic cells were escaped from the selection pressure. Thus, longer time of shoot selection with higher hygromycin or G418 concentration may be helpful for eliminating the chimeras.

Comparing the barley transformation system with immature embryo explants (Harwood 2014), our new method can significantly shorten the transformation cycle but showed lower transformation frequency. However, more experiments including more genotypes and more vectors are needed to evaluate the advantages and disadvantages (chimeras and lower frequency) of the new transformation method. Anyway, the shoot tip transformation offers an alternative method for the traditional transformation techniques.

### Phenotypic variations during shoot tip transformation

Transgenic plants may show undesirable genetic changes that can reduce their agronomic performance. Therefore, it is extremely important to maintain the genetic identification of the WT or minimize recalcitrant variations for the transformations of commercial plant cultivars. Thus far, despite several transformation systems have been developed and used for crop transformations, the shoot tip transformations were generally considered as the best method to produce transgenic plants genetically identical to the WT (Schrammeijer et al. 1990; Sticklen and Orabya 2005; Baskaran et al. 2016). In this study, we generated transgenic plants using the barley shoot tip explants which were not subjected to callus induction, thus should produce plantlets identical to their WT plants (Sticklen and Orabya 2005). However, visible morphological changes were observed in both T1 and T2 transgenic plants (Fig. 5, Supplementary Fig. 2). As the specific PCR bands were amplified in the T1 plants of 001320-002a-5, 001337-008a-6 and 001337-002a-3, we cannot rule out the possibility that the mutations in these plants were caused by position effects of the insertions of transgenes. However, no expected PCR band was found in the T1 plant 1337-003a-6 suggesting the phenotypic mutations can also be induced by the meristematic culture and/or transformation processes.

Plants with agronomic changes such as heading date and plant height have been reported in the shoot organogenesis process using shoot tips as target explants (Bregitzer et al. 2002). Therefore, it was not a surprise to recover transformants with abnormal phenotypes in this study. Thus far, the mechanism causing the somatic variation is not well understood. It seemed that shorter duration of tissue culture or skipping callus formation may reduce somaclonal variation (Larkin and Scowcroft 1981; Sticklen and Orabya 2005). The molecular mechanisms of somatic variations are still poorly understood but the previous results suggested that abnormal cell division, point mutations, unfaithful repair of broken DNA, methylation changes and activation of transposable elements contributed the induced variations (Bregitzer et al. 2002; Gao et al. 2009; Anderson et al. 2016; Coronel et al. 2018). Therefore, further comparison of the genomic and epigenomic changes between the mutants identified in this study and the WT may provide insight into the mechanisms associated with the genetic variations during shoot tip culture and transformation. Notably, we cannot exclude the possibility that the phenotypic variations in T1 and T2 generations may be caused by the complex genetic background as chimeras were reported in many transgenic plants and caused visible morphological changes (Schmϋlling and Schell 1993; Faize et al. 2010).

### Application of RNAi for improving barley FHB resistance

The applications of the synthetic dsRNA targeting some important genes in the fungus, including cytochrome P450 *lanosterol C-14α-demethylases* (*CYP*) (Koch et al. 2016), *Argonaute* (*AGO*) RNase protein and *DICER* (Werner et al. 2020) and *Tri6* (Hao et al. 2021), may reduce the fungal infection and improve the FHB resistance in wheat and barley. However, direct spraying of dsRNA on plant surfaces can only provide a short protection window (5–20 days) against the pathogens (Mitter et al. 2017; Worrall et al. 2019), and the protection cannot be transmitted to the next generation. Another strategy is to use the host-induced gene silencing for which the pathogen-related dsRNA was transferred into host genomes, and the transgenic plants can generate dsRNA derived from pathogen genes to protect plants. Cheng et al (2015) transferred the inverted *chitin synthase* (*Chs*) 3b fragments into wheat and found that the transgenic plants conferred high levels of stable resistance to FHB in the field and the expression of dsRNA derived from *Chs3b* gene significantly reduced mycotoxin accumulation in the grain. In this study, we transferred *Tri6* into an elite barley variety and our molecular and sequencing analyses demonstrated that the foreign genes were integrated into the barley genome and can be passed to subsequent generations. Therefore, our experiments offered useful resources for understanding the molecular interactions between the fungus and barley and other research. It is important to note that we found segregations of 13 T1 transgenic lines including 001337-2 likely represented a germline transformation event as all tested leaves of the T0 plant and the majority (27/29*100 = 93.1%) of the T1 plants were PCR positive (Table 3). The segregation ratios of transgenic plants to non-transgenic plants in three T1 lines, 001320-7, 001337-11, 001337-16, were about 3:1 implying a single copy of transgene in these lines. However, as the seeds of each of the T0 events were harvested and mixed and we cannot rule out the possibility that the seeds from chimeric (non-transgenic) heads may cause some bias on the estimations of transgene segregations. Also, the sample numbers of T1 plants were still small and it is possible that the two T1 lines, 001337-5 and 001337-12, may contain some transgenic plants.

We conducted FHB evaluation under controlled conditions, and no significant differences in disease severity and DON concentration were observed between the transgenic lines and the WT. However, this preliminarily phenotyping cannot provide solid evidence on that the HIGS cassette targeting *Tri6* is inefficient for improving the barley FHB resistance and reducing DON accumulation in grains. The expression of FHB resistance is highly influenced by many environmental factors and plant morphological traits that make it extremely difficult to reproduce phenotypic results (Cuthbert et al. 2006; Graham and Browne 2009; Srinivasachary et al. 2009; He et al. 2016). Hao et al. (2021) sprayed *Tri6*-dsRNA on wheat and found that the treated plants did not reduce FHB spread under controlled growth chamber conditions, but reduced FHB spread and DON production under greenhouse conditions. Wheat and barley exhibit different FHB resistance mechanisms, barley has natural resistance to reduce the spread of the fungus (type II resistance) whereas wheat combines two primary types of partial FHB resistance including type I (resistance to initial infection) and type II (Mesterházy 1995). The inoculation methods used in wheat may not be suitable for barley FHB evaluation. Therefore, novel and creative protocols are urgently needed to evaluate barley FHB resistance in greenhouse and growth chamber and to collect reliable and reproducible results. Additionally, it is necessary to phenotype the FHB resistance of the *Tri6* transgenic plants under natural field conditions for multiple locations/years with large sample numbers. It is also possible that the choice of a partial GFP sequence as the connector between the *Tri6* silencers in the inverted repeat may not perform as well as an intron sequence as used in other RNAi approaches. Furthermore, recent research suggests that it is now possible to exert even more control on the expression output of transcriptional units via use of alternative terminator sequences (Diamos and Mason 2018). The rearrangement of foreign genes in transgenic events generated via Agrobacterium mediated transformation was reported in other plants (De Neve et al. 1997), it is not clear whether the tandem organization of our transgenes affect the efficiency of gene silencing. As chimera is common for the shoot tip transformation, despite we used the transgenic plants/lines that were validated by PCR, we cannot rule out the possibility of that chimeric genetic backgrounds in some plants may infected the FHB evaluation results. Furthermore, due to the limitations of transgenic seeds and the growth chamber space, we were not able to evaluate more samples which may also cause some bias, thus more samples and multiple rounds of FHB evaluation are necessary.

### Supplementary Information

Below is the link to the electronic supplementary material.

Supplementary file1 (DOCX 1234 KB)

## Data Availability

The Nanopore long-reads containing the vector and barley genomic sequences are included in the Supplementary file of this manuscript.
