# Supplementary material for: Agrobacterium-mediated transfer of the Fusarium graminearum Tri6 gene into barley using mature seed-derived shoot tips as explants
Source: Plant Cell Rep. 2024 Jan 20;43(2):40. doi: 10.1007/s00299-023-03129-z (PMC10799836; doi:10.1007/s00299-023-03129-z)
Supplement: Supplementary file 1 — Supplementary file1 (DOCX 1234 KB) [file 299_2023_3129_MOESM1_ESM.docx]

Table S1. Summary of dd-PCR analysis in T1 and T2 transgenic plants

| T1 plant | Copy number of T1 plant | No. of tested T2 plants | Copy number of transgene | | | | | |
| --- | --- | --- | --- | --- | --- | --- | --- | --- |
|  |  |  | 0 | 1 | 2 | 3-5 | 5-10 | >10 |
| 1320-002a-2 | 2 | 8 |  |  | 8 |  |  |  |
| 1320-002a-3 | 2 | 8 |  |  | 8 |  |  |  |
| 1320-004a-3 | 5 | 23 |  |  | 3 | 8 | 12 |  |
| 1320-007a-1 | 1 | 14 |  | 10 | 4 |  |  |  |
| 1320-007a-2 | 1 | 15 | 3 | 7 | 5 |  |  |  |
| 1320-007a-3 | NA | 8 |  |  | 8 |  |  |  |
| 1337-002a-2 | NA | 16 |  | 12 | 4 |  |  |  |
| 1337-002a-3 | 1 | 8 | 3 | 4 | 1 |  |  |  |
| 1337-002a-7 | 1 | 18 | 1 | 10 | 7 |  |  |  |
| 1337-008a-1 | 7 | 8 | 1 | 2 | 1 |  | 3 | 1 |
| 1337-008a-5 | NA | 8 |  | 1 |  |  |  | 13 |
| 1337-008a-6 | NA | 8 | 1 | 1 |  |  | 1 | 5 |
| 1337-015a-3 | 1 | 8 |  |  | 8 |  |  |  |
| 1337-017a-6 | NA | 8 |  |  | 8 |  |  |  |
| 1337-017a-9 | NA | 8 |  |  |  | 5 | 3 |  |
| 1337-017a-19 | 3 | 8 |  | 1 | 2 | 4 | 1 |  |
| Total |  | 175 |  |  |  |  |  |  |


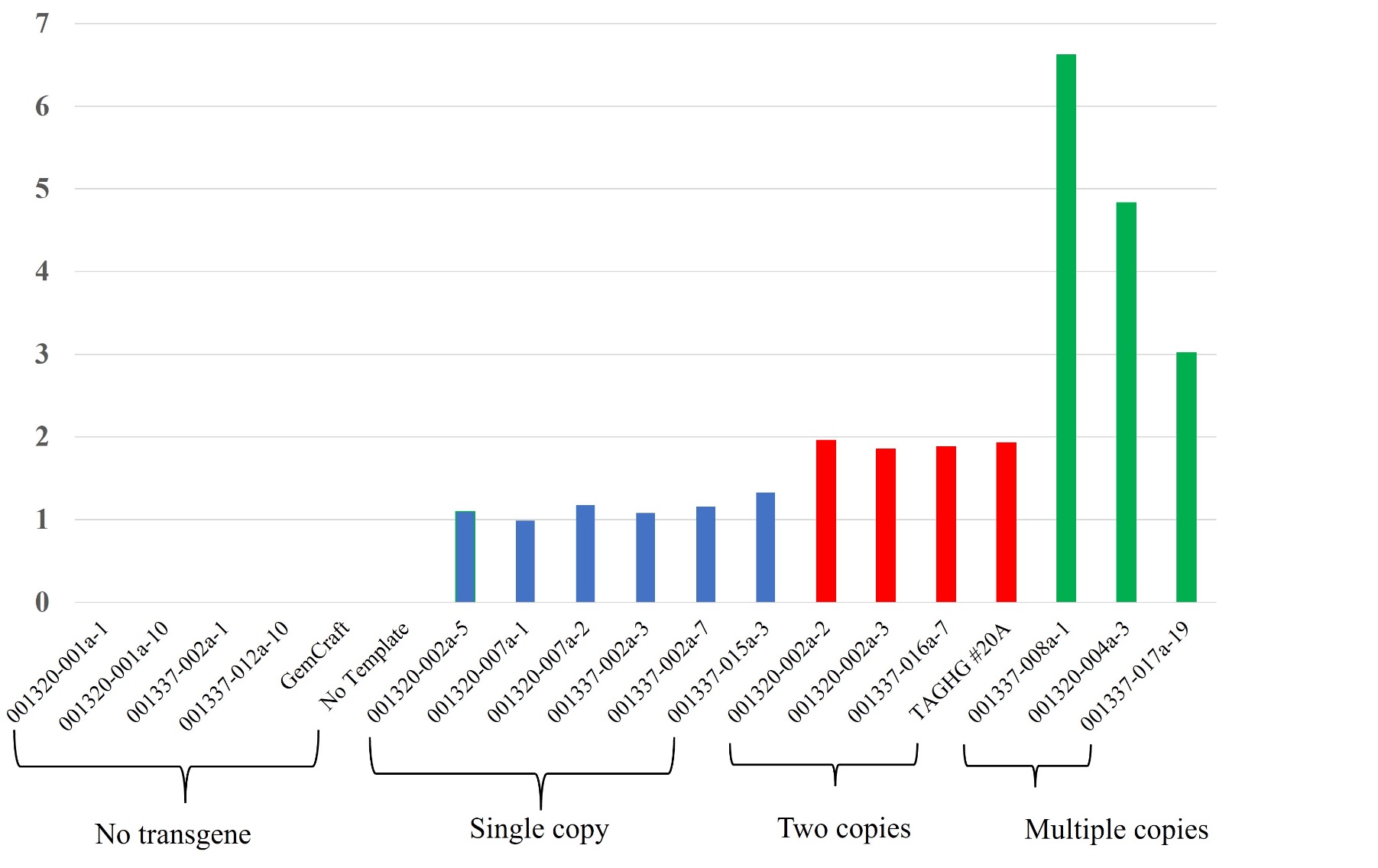


**Figure S1. DD PCR analysis of T1 transgenic barley plants**


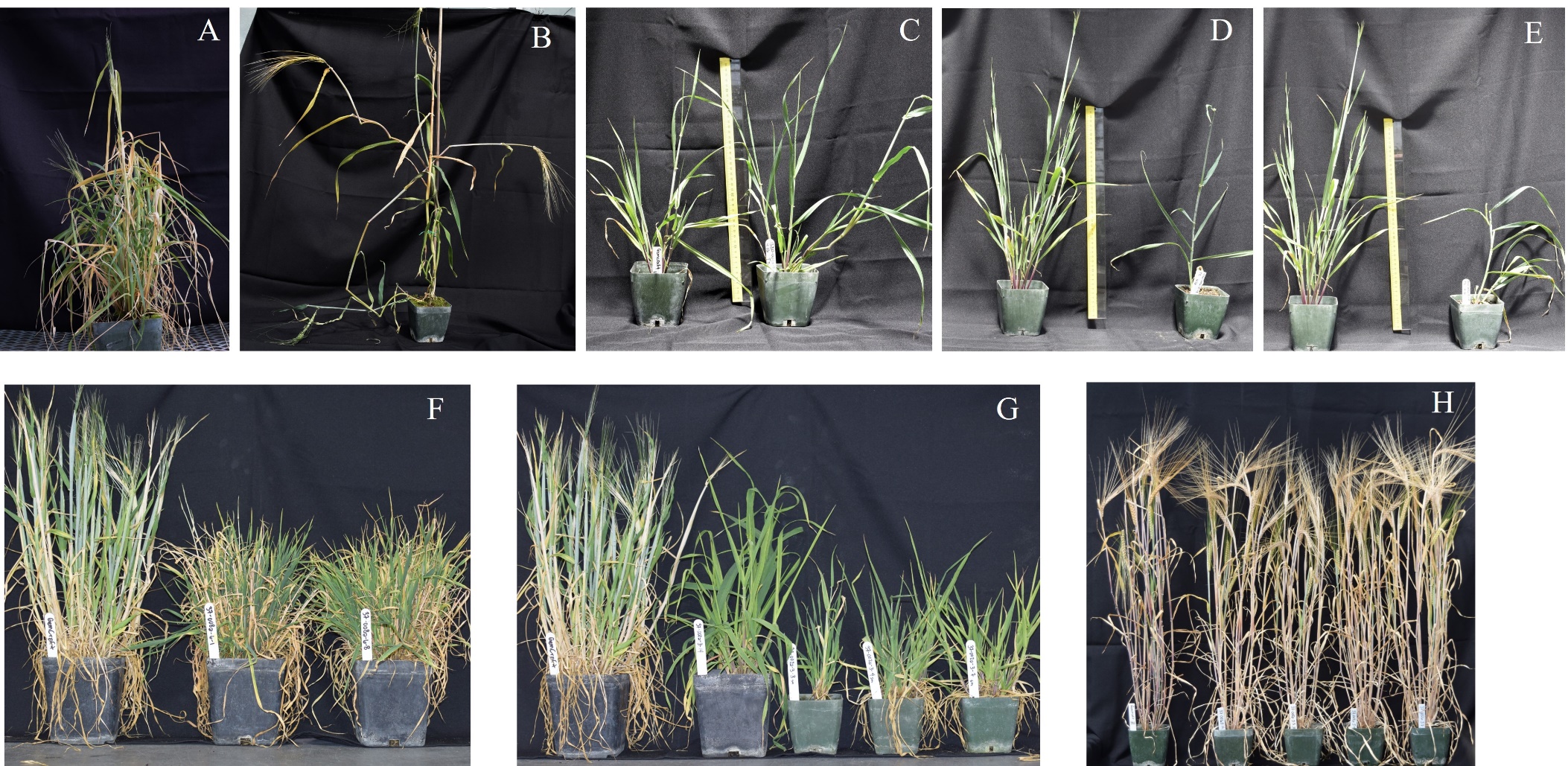


**Figure S2. Phenotypic variations in T1 and T2 generation plants.** A. 1337-003a-6 (T1 plant); B. 1320-002a-5 (T1 plant); C-E. 1320-002a-5 (T2 plants); F. 1337-008a-6 (T2 plants planted on April 16, 2022); T2 plants of 1337-002a-3 planted on April 16, 2022 (G) and January 25, 2023 (H).

**Supplementary data**

>ONT1

CGTCACTTGTACTTCGTTCAGTTACGTATCAAAATTGCTGAAGTCCCTTCAGCTCCCAGATTTCGCTTTATCAGACTTTCTTCTGAAATTCTTAGCTTTTTATCTACGATTCCAAATGCAGGATCACTGCCAATCTATCATTATCATGATTAAGTTAGATGACGCTTAGATTGTTATTGATAAGGTAAGCATAGAAGCATTAAAACATATGAAACTTGTCAAAGTATTTTTTTCTTTGGATCAGCATACCGGTTTTTAGGAGATGGCTTTGCAGGATATTTTAGTCTAAGTGATCGGATTCGGCCAATGTTAGGAGAGATGCTCCACGACAACGAAGGGGACCTGAGACCTGTTAATTTAATATGTTCACTGTTAGAAAGTTTTGCAAAACTATATCCGCAACTGTGGTGACAATAGAAGGCCCAAAAATATGTGCTTACCTTCATCTCAGAAGCTTCGTAGTGGCGATCTGAATACTTCAGAGGCACGTTACCAAATGCGGTAAAGATCTGAGCCCATCAGTATTTGCAAGTATCTTAAAACCACATTCTACTGTAGTAACAGCTTAAAGAAGATTGCCTTCTTTATTGAACCTTAACCGTGGATAGCTCTGTTAAAATTTACATGAGGCTAGATTACTAAACGGACAAAAAATGTTGAAAAATAAAATAGGGAACAAAAAACCCACTGGCAAGCCTCCACCAGCTTCAATGCAGGCAAGCATGTTGGTGTCATCGACATCCCAAAACTTAATTTGGTTATCTTCGCCAGCAGCTAAAATGTGATTCCGAGCTGTATCAAACTGCACGACACCCTGCGCTACACCAGATGGCTTTTTACGGAATCCAGAATATGACCTCTCGACAAATCCTTCACTTTGGTTCCACTCAACCAAATGTGTATCTCCCCTCTTTGCTGGTTCCACAAGAGAACAACCTTCAAATCGACAACACGTAAATATTACATCACCAATAACTTGAAACACTTCTAGCGAGCAAAATAACACATCTATGTTTACCTAGTTCCATCGGCGCTATAGAGCATTGCACTACACCATTTTCAGGAACATCAAAGTCCAGCCTAGATCCCAGATTGTCATAAAGCCATGTCTTCATTTTCCCATCAACAGAAGTTGAGGTAATAAACTACATGGTACACAATAAAATTATGTATCAGTACACCAGATAAATGCTGATGTGCTTGAAAAAACTATGCACTCGTAAATCTCTCTCAGAGCACAAAATACAGGCATCGTCCTCGTGATGGCTCGTACTGCCACACTTGGATTATAATTGAGTAGAGGAGCAACTGCCATCTATATCCGTTGCATTCTAAGATGTTAATGCCTAAATATTGTGTTCAGACAACACACACCTGAACCTGCTCTATGTGATGAGGGCAAATAGAATACACAGGGGCCTTATGCCCTTCAAACGAATATTTTCTGTCCATGCATGTCCCAGACCTACAAAATGGGAAGGGAAGTTATAGGAACCAGGCTGGGGGAAAGGAAATAAATGACAACCATTTAATTCATGTCAGATACCCTTATCAGCTTGTCATCTTCATAAAGTGACAAGACAAAGTTAATGCCCGGGAGAAAACTATGTCATTGTTACCTCGGAATGAGCCTCAATCTGACATGAAGAATGAATGGGTTAGTAACAAAAATCATTTAATATAATGAAATGTAACATCTAAATATAATTGAAGTTCAAACGCAATTAGAAAGAACAAACCTACAAATTGATATATTTTATTTGGATGCCGGTATACATGCAGATGGATCAAATGTTTTGTAGATGCAACTCCTAAAAGGTTTAAAAATAATTAAGCTTAAAAGCAACAATGGATCACAATACAAGAAGAACTTACTGGGTGCCATATATATAGAGTCACTGGGTGAAGACTGAATTATTAGCACTAACCAATCAAGTCTCCATCAGGGCTCCATGTAACTCGATTAACGGCCATGGAAGAGTCTTTAGCCATGACACCCTTCTCTCATACGATATAAAACAATGATGCACATATATAATATCAGAACTATTGGGTTTGCAAACAAAAAGAGGAGCAGACTACATACAAAAGTTCAGCTTGTGCCCAGTTACCTGAAATTGTGTTGAACATGCTTGCATGTCCCAAATTTCGAAGGGCTTTGTGACCAGCCTCTCACTCGTACCAATCTCCCAGAGCGTAAATTCACCATTAGCAGATCAACTATTTGAGCAGTAAACATGACAAGAAAATCAATGTGGTGTTGAAATCCGAACATTCATCCAAATCATGAAATACCTTTTTGTACTTGCAAAGTTGCAACTACATCTATTCTTTCCTGATATAGTAAATTAGCAACCCTTGATCATACCTACTAGTAGCGTATGGCGAGAAGGATGAAAGTCCATGCTAGTTACATTAGATCCATGTGATAACGTGCAAGCTACTTTCATAGGAAGGTCATCCAGTGACCGTGAAGGGATAGGAATATGTCCAGTGGGGCTCAGTTGCTCCATCGATGATCCGGAATTCTGCAATTTGATAGTCTGTTATATTTGGGCGTTTTCATCACGAAACCGATTTGTCATAATGCAATATAAGTACAGAACTGAGGTCACAGACCACATCGACACATATTAGGCTCCCTTTTGGTTCATATGATAGAAAAAAAAATACAGGAATAGGAAAACACACAAATAAAATAGGAATATGGTGGAGAACAAAGGAGTAGAAAAACACAGGAATTTTTCAAGTTAGTCATTGCAGGAAATGTAAAAAAAAACAAATCTAATTAACTGAAGTGAATAAATTGGTTTAACAAATCTGTTTATAGCCAGCAGCTTGCCTAACAGCATAATAAGAACATGTCTTATTTGCTCAATAAATAGACCCATATAATGCATGTCAACCATAAAGGAAAGTGGATTGAACCGAAAGAACCAACTCACAGACCCGTTGACGAAAAAAAGAAGGGAAAAACAAATAAACAACTCGTTTAGGAACACACCCACACAAACAGTCAAACTGTTCGCACAAGCTGGTTGTCAAAAGGTAGGCTTCCTGTAATCTGCGGTGCAGAGAAGCCTTTGTATTTGTAATCATCCGTTGGTGTTTGGAATGGTTTCTACAACTTGAGATATTGTAGTTGGTAGATGGTGTATACAAAGTCAAAAAAACGCGCATAACATAAAAATGTGAAGGCAACGCTACTCTTGCATATATCCACACAGAGATACATGCATCAACGAAGATATAACCAAAATTCATTCTGGCACTGCAGTCTAAAACAAACTTTTGTAACCACAGGTCTTGCACATGACTCAACCTGGGACTGGGTCGGTTTGCCTGGGTCCAAATCCGATGTTTTTCGTGTGGTTCTCAAACAGCGCGGTTCAAACGGGCTAACAAGAAACTGGGCTAGACTGGTTCAGCACATTACCGACCGTTGTTGATACTTTAGAAAGCACACATGTCCAGCCAGATCCCGATGTTGGAACTCGTCCACGGATCAGTCACATTAAATCACAACAAACACGCACACAGAAAATCTTATAGCCAAGGGTCCTTGTCGTGTCCTAATTTTCTCTTTTTTTATTTCTTCTTTTTATCTCAACTCATGGTGTTACAAAACCCACGTTAACCCTGCATATATATTGACCTGGCTAACCATCGACTCAACCTAGACATCCGACTCCAATACACCTTTGACCCAAAACCTAACAGAACTCTGCTAATCTCAGCCTATCTTGATTGTAATTAGCCTAAATCTATTTGCATACAACTAGTACTACTCTGGCAGCACTCGTGTATACGACTTTAACACCACAAACTTGCCTAGATGCTACTAACAAGCTGACCAGCTAACCCGACCAATCAAGATTATGGTAACAATCTCCTCCTAATTTTGATTTCTCCTAATATGAACTTGTCATCGCTTTGTACTCCTCCTGATCTTGGCCCGTTGTAGATCCACGGCCACGACGATCCATCTCAACGTGTGATCCTGAATTCCCGATCCGCTGCAGGACGACATCATCGGTCACCCCTAACGCAATACATGTCAACGGATCACACACCATCCCATGTAGAGACGGAGTCCTCATTGAAGGCTCCCCTTCACTGGTTATAGTCCCACTGCCACGCTTGAGCTTGATCTTCACTTCACGTCTTCGCCCTCGGTGCTACACCACACGGGACAACACATCGACCGTGACACGCTTCATCTTCAACCGGACCTCACGCAGAGACAAGTACTTGCACATGACGACGATGACTCAACGACACTATGGCCAAGATGTGCCAACCCCTGCTTTACTACTTGACTCCCTCGGACAACTCGACCGAACGTGCGAACTCGCACGACTATTTGACTCAACTCCCTTGGACGATGACCTTGATGCCTTCTTGGCACCGCACATCGGCACCACTGCTCCCATCAACGATCTTCTTTCACCGCCTTGCCAACGACGAGCGCTGTCCCTCCTTGTCGCTTCGCCCCGAGCAGCCGATCTACGAACCTCCTCATCCATCGCTCCACGAACGACTATTGCCGATCCTCGCGGTCGCTGCAGCACTCAGCGACTATCACCGCTCCTCCTCGCCGCTACACCATCGACTATATCACCGGCTCTTCTCGTGCGATGCACCACCCAACAACTATGTGCCCGCCCCGAGGGGCTGGCGGGGTCGCCACCCTAGGGCAAGCGCGGCCTCACCTCGACCTTGCTCTAGATATCAAATGTTGAAACTCGCCCATGGATCGATTACATCAAATCACAACAAACACGCGCACACACAAAATCTTATAGCCAAGGGCCCTTGTCGTGCCTAATTCCCTCTTTTTATTTCTTCTTTTTTATCTCAACTCACGGTGTTACAAAACTCACGTTAACCCTGCATATGTATTGACCTCGCCTAGCAAAACCGACTCAACCTAGACATGCGACTCCAATACACAGCTGACCCAAAACCTAACAGAACTCTGCTAATCTCCGGCTATGTTGATTGTAATTAGCCTAAATCTATTCGCATACAACTAGTGCCTACTAGCCTTTCGTGTATATGACTTTAACACCACAAACTTGCCTAGATGCTACTAACAAGCTGACCAACTAACCCGACCAATCAAGATTATGGTAACACCCGATACAGATCCTGTAAGTATCCTTTGATTTATTGATTTGAAAAAAAAATAAACTAATTAATGTCATGTGTGGGCATACTGCTGCGATACATTTTGGATAATGATGTGCGTGTGAGTGTGATGTGAGAATTATCAATGTGGTTACCTTGGTTGTTAATTAAGCAATTATCCATACATCGTCCATCGAAGGAAAATGAATGAATCATTGAATGAACTACAATATTTTTGAAGACTCCATTGGTTACATTGTAAGCATGATGTGTCTAACCATTTCATATTCTATTAGTATTTACCGCTGTACTAATTAAGTATGGTTTGATGTCATGTTTCCATTTGAAAGGTCAGATGATCTAAAGGAAACCTGGAATATTTGCTGGAAGACTTCATCACTTCAATAAAAAGGTACACACTTCCTATAAAAAATCTCCTTATTTTTAAAATTTTAAATAATTATTATCTATATACAAACTTATCCTTGTATCATAGTTTAAAGAATTTTGGTGTATCGGGTGTATCCTTGTATCGCATATCCGATACGTACGTATCCAAGTATCAGAGCAACATAGATTAATGATCTGGTGATCCCAAACCGCACATGTGGAGATAATAAAGCTCCATTTGAGGTCATCTTGTATCCTGTGAATACTATGCTAAAATGACAAGTAGTGTGATGTTGCTACCATGCTGAAATGCAATTCTTTGACTTTGTATGGTGATGCTACCTTGTATGGGTAGCTAGTGCTTCTGTTGTGCCTAGGAAATCAGTGAAGTTGCTGGTATTAAATTCTGATGCATAGGATCCATCATGTGCTATTAGTTTCATGAGTTTCTAACCTCTTTTTCTAACCATCTTAAGAGACTTCCTTCCTTGCTGGCACATGTGGAATGCTGATGTGTGTAGGAATAAAAGAGATAATTCTGTTTTCCATGTCAAGAAATGATTGTTCTTCTATGCCGAACATTTCTTTTCGGCAGGTTTTTCCATTGTCAACCAATCTATGCTTCATGTAGTACTATTGATTGTGGGCATGGGTAATTTGAAGGTACGTTTCGCCGTCACCGTCACTCTACTGCAGTGCCTTAAATCGGATTTTTCATATTTCGGACATGTTTTGTATTATTAATAGATGGAGATGCTCTAGAACACTTCCATAATGTTTTCCTTTTCTTATGAAAAAATTGTGTTGCATCGGGAAATACCATGTGAGAATTTTTTTATATCTTGCACTATTTTAGATTCTTTTTTTATGCGCCATACTGCACTCAGTGAAAGGTTCATGTAGAATTATGGTAGGAAATGTCCACTGTATGGACACCCGTACTCATGACTCAGTTATGGAAATTATCATAATATGACTCAGTTAGCTGTTGTGTCAATCTGATAGAATCCTTCGTTTGGCAGTCCGTGTGTAAACTGTGGAACATCAAGGTTTGATGAGCTATATTGTGCCAAATCCACCAAGTAACTGAAATGGACCAAGAAGCAGGTCTAAAGACATCAGTGAGTTGGTTTAATTTATATATGGGCATGGAGCAGATGACATCTGTCAGGCTTGTCCTGTCCTAGGTTGACAAACACATTAAATTTTAGATGATTTTTTCATATTAAAAATAAATACTAAAGCATGCCTATCATATGCAATGTTCATAAGAGAAAGGGTGAAGAGATCCATCCATGGCTGACTTTGATGTATAAATTGATGTTCCTACTGTAGCTATGGAAGCTTCTGGCAAAACCAAGGGAGCGAGGAAGGGTGGAGCAACCCTGTTATCTAGTTGCCTGCCTCACCCTCCCCTCCATCTCCTCTCCTGGAATGGACGAAGATGCATTGTCCAAGAAGACGTGCATTCCCTGTGCTTCCATTCATACTTCCTAACAGCTTCCTAATGGACTAGCAGGAAGATGGTGTGAAGATCCTTCCTCTCCTGGAAGGACTGCCTTAGTGAAGCGGGCCAAGGAAGCAATCCTCGCTGCCCCAAGCGGTCAAAACTCCGCTCAAAACCGGATGCAAAATTGCGCCCGTGATGCAAAACCAGCCAAGCGGTGCTCTCTCGACTGTCCTACATAATCTTCGCCGTCAAATCGTGCCTCCTCCGAAGAATCGGCTCACATACGCGGAACGGAGATGCGAAATTATGAAAAGTGGGGAAACTAAGATGGGAATCGCGAAGAAGAAAAAAACGAAGATTGGTTGGTGGAGGAGAGACCTTGGTCTGCGACCGGCGCCGGGAGGTGTGAGGAGGGCCGGTGAAGAGGGATCGGAGCCTGGGAGGTGGGATCGAGAGATGGCCGGAAATGACAAGGGGGAGAGAAGAGAGAGAAAGAAATCAGTACGTAGGAGATGGGGAGATGGAGAGGAAGAGGGAAGGAGGTGGACTCACCTCGAATCATGCAGCTTGACCGCCAGGCCGCGACGGGGCAGGATCCAGAGTCGCCGGCGGCGCCTCTCCTCGTTTCTGTCGCTCGCTCGGCTCCGACCGCCGCCGTTTCTGGCAGTGGACACAAGACTGAGGGATGACCTGTTTTTTGAGCAGCTGCGGGAGCTTATATTTTATAGAGAGGAAAGTGCATGGGTGCAGCGGCCTACTAAGTACTAACCCAGCCCAGCCCAGCCCACCTATCGGCTTTGCCCAGTCCCGAATTCAAGCTAGCCCATGTATGCAAAAGAAAAAAAAAAGATCTTCTCAAACAGGAAACAATAAATACATTCCACTCCAAGAAACCAAGGAAAAGGAAAGCAATACTGTACGTGTTAATGAAAAATGGATGGAATACAGTCGCTTAGTTGACAAGGACGGCAAACTAATCAACGGATGTATTCATCCGCAACAAGTGTTGCAGAGACGATGCGGTATATATGGTAAATCTAGTCGTCTCTGCGTCGTCTTCCGCTCTCACCGGTCACCACCGCCGGCATGCGTTGTTTGCGCCGCCACCTTGCATCGCCGCTCTTCTTGTATTTCTCGAACACTCTTCTTTTATGGTTTTCTCTCAAGAACACACACCTTTGTAAAAACTCAGAAAACACACACACGGATCTATATACATATGTATCAAGGAGGAGCACAAAGCTTTGCCGGGAATCTCTTCTCCTTGGTGAAACAAAGACTACATGTTTTCTTCCGGCCCTCATGGACTCAATCTTTTCTCATTCACTTGTCCCCGCCCAAATAGTAAGTACGGAAGACCTACGACGGCCACGCGGCCACCACGTCTCTCCATGCACTAAGGGCGTGTTTGGATGGATGGGTCTCACATCACACCGGCGTGCAAAATCCAACGTGTTTGGTATCTGCGCGGCCTTCTCGGCCTTGCCTCTCGGATGCAAAGAGGACCTCTCGGCCAGTGAGGAAAACGTGAGAATCGGGACGTATCCGCTGACCCGGGCTCGCGCATGCATCGCCCTTTCCTTTCTCTCGCTCTCCCTCGCATCGGATCCAATCGAACACACCAACCCATCTTGTCGTCGTCTAAGTCCACTTGTCCGCCGGCGGTGTGAACTCCCTCGCCGGAGATCGAATCCGTCGGCGCTTCTACATGCCCACGTCGTGGAGCTGCCCCGCCTTGCTTCGACATCTTCGGGTTCCGTCCCGTGTGAACTCCCTCGTCGGAGATCGAATCCGTCAGCGCTTCTCCATGCCCACGTCGTGCACTCACCGCCTTTCTTCGACATCGTCGAGTTCCGCCCGGCCCCACGCAAATACGACATCACCGGTGCCGGCCGGCGTGGGTCCTTCAGTGCCACTCCTTGCATCGCCCGTTCTTCTTCTACCTCTTGGACACTTTTCTTTCATGGTTTTTATAAGAACATACACATTGTAAAAACTCAAGAAAATACATACACACACACATGTATATACATGTACCAAGGAGGGGGCACAAAGCTTTGCCGAGGGAATCTCTTCTCCTTGGTGGACACAAAGACTATATGTTTTCTTCCGGCCCTCATGGCCTCAATCTTTTCTCATTCACTTGCCCCACTTACATAGTAAGTACAAATGACTAGGCAGCCACACGACCACCATGTCTCCGGGCACTAACAAACTCATGCACTAACCCACTAACCAACTAACTCATACATGCATGCACACACACGTTCTGCTATATTTCTCGAAATAGGCTTTTTGTCGAAAAAGGGTTTCCCCCGCTTTGTATTACAAAGCAACCAACCGATACAATCAACAACGAGGTCTTCAGGACGAGCCGGCATAATCACACCTAAAAAGAAGAAAGAGAAACAACAAAGAGAAAAATGCCGACAACGGCGGATCAACGGATACAAAGACGCTTCCCGGAGAATACTTGTGCCCAGAAGATCTCCCACCAAGCTTCAAGGCTCCCGGCTCACCGTGCAATCAACACCTCCAAGAAGGACCGCGACGATGGCCATTGCTGCTAGGGTTTCCCCGTGCGTGGCCGAGGCAAGAGGAAGGGTCGCCCAGCGCCCTCCCGGGAAGGTCCGGCGGCACCCACAAGCGTCGCCGCGTCGGTGTCGGCCGGGCGGCAGGGGTTTCCCCCCCGATCCCAACCTTCACCCCTCCAACTCTGAGCCTGCCACCAAACCGACCACCATCTTGCGCCACCGCGGTCATCGGAAACCTGTGCAGTGCACGGCACCATGAGTGAACTGCACAACGGGGGACGGGGTAGGGACTGGGGGCAAAACACCGTCGGCACGCGGGGAGGGTACAACCTCCACGCGTCAATGACGGTGACGGCCGGCAAAATATTAGGAGCATACCGAGGCCCGTGGGCCCACGGGGCCCAACCGAGCTCTCACGCCGTCGCTCCCGCCATCGCGGCGCAACGGGCGCGTCATCGGCGTCGCCGCCCCTCGTCCTAGCTGCTCTCCTCCTCACCACATGAGCCGATGAAGCCACGCGGGGAGGCGGCCCGCGAGGACCCGATGGGGCCCACGGGGCCACGATGCAGGCGGGGCGCGCCAGAGCCAACCTGCGTCACCACGCCGTCTCATCGTCAAGGGGGCAACACCACCACCTCGCGCGTCGCCGTGCCTAGCTGTAGGGTCGCCGCCCACAAATGCCCGGGCGAGCCGCGCGGGGACATGTAGTCCTACCGCCGCCAACACCACCCGACCAGCGGGGCGACCGGCGGCTTGGCCGGGGAGGGGGAAGGGGAAGGAGGACGAAGGGGAGGGGCTCGCGCGCGGCCACCTCTGGGGAGATGGCACGGGACGGATCCGGGAGGGGGAGGGGTGCGGGGACGGCGGCGGCGGCGGGGGCCTAGGAGGCCATCGGCGGGGGGGGGGTGAACCCTGGGGAGCGGGGGAGCGGGGGTAGGAAAGACCGGAGCTCACGTGTCATCGAAATAGGCTTTAAGCCCCGCGCTATGGATATAACAACCACGAGATACAACATCTAGATCAACGGCGGGACGGCACAAGCACGCCCAAAGAAAATACGAGAGAAAAATGCCAACAGCAGCCGGATCCTTCGAAAATCGCCTCCCGGCGGTGTATTTCGTGCCGCAAGATAACCACCATGCTCGAAGCCCATAGAGGTTGTCGTGTACCAGCACCACCTTCAAGAAGGAATGCGACGATGTCGGCAATGGCACATGGACAGATGGTTCTCCCGGTACACGGAGGGTGGGGGTGAGAGGGTCACCGACGCCCCTTCAAGAAGGATGGCGGCGCTTCCCGCAGCGTCACCGCGTTGGTGCGGCAGACCGTCGGGATGCCCGGCCACTTCGCACCGCCACCCCGGGACCAACCTTTGGCTCCCTTACACCGCCGCCCACCAACATGCGGCAACACGGTCACGAGGACGCCTTTGCGGGTCGGCTCACACGACCCAACGAAGCGAGGCCGGTCGGATTCAAACGAGACACACGTAAACATGGACCGACAACACCACAACCACAAGGAAGGAACAACCGCCCATAGGCATAGGCGATGGCAGAGACCGTACATAGCGAGTGAGCACACCCCGAGACCAAATGTCCGGCCCGAGCCCGAACGGGCATGTGAAGCTCCACCGCCAAGGCTGAGCGGTCGCGGCACAGCAGGCGCCGTCCGTGTCGTGAGGAGCTCGCCAAAGCCACTTAGAAGAACAACCCACCGCGGCCAGCGGGGCCCGCCCCGGCCAGGATTGGGCCCTTAGGGCCTAGATCTAAGCCGGTCGGGGGCAGCCGGCCGCCGCAGGCCATGGGCCATAACCGCCGCCAAGGGCAAAGCCTCCGCACCGCCACCGTCCGGGATCCGCGCCGGACGCCAGGTGAAGGGCTCGCCGCCGAGGACCGCATGCGGCGGGGTCCGACGGGGAAGAAGGGGGCCGCCACCGCCGGACTGCGGGGCGCCGTGCGGGCAACGCCGTGTTGGAGATATGCCCTAGAGGCAATAAAATGGTTATTATCATATTTCCTTGTTCATGATAATCGTCTATCGTTCATGCTATAATTGTATTAACGAAACGGTAATGCATGTGTGAATGAATAGATCACAATGTGTCCCTAGCAAGCCTCTAGTTGGCTAGCTCGTTTAGTCAATAGATGATCATGGTTTCACGATCATGGACATTGGTGTCATCATTGATAACGGGATCACATCATTGGGAGAATGATGTGGTGGACAAGACCCAATCCTAAGCCTAGCACTAGATAAATGCTTGTTCGTATGCTAATGCTTTTCTAATGTCAAGTATCTTTTCCTTCGGCTTAGTGAGATTGTGCAACTCCCGGATACTTGTAGGAGTGCTTTGGGTGTATCAAACGTCACAACGTATTTGGGTGACTATAAGGTGCACTACGAATTTTCAAAGTGTACGGCACGAATCGAGATCGGGATTTGTCACTCCGTGTGACGGAGAGGTATCTGCAGGCCCACTCGGTAGAACATCATCATGAGCTCAATGTGACTAAGGGGAGTTAGTCACACGATGACGTGCTACGGAACGAGGTAAAGAGACTTACCGGTAACGAGATTGAACAAGGTATAGGTATACGAAGCCGAGTCGAATCTCGGAGCAAGTTCTATGCAAACCGAGACAAAGGGAATCGTATACGGGATTGATTGAATCCTTGACATCGTGGTTCATCCGATGAGATCATCGTGGAGCTAGTGGGAGCCATGGGTATCCGGAACCCCGCTGATGGTTATTGGCCGGAGAGGTGTCTCGGTCATGCACTTTGTCTCACGAACGTGGGGTCTACACACTTAAGGTTCGATGACGCTAGGGTTATAGGGAATTGTTATACGGTTATCGAAAGTTGTTCGGAGTCCGGGATGAGATCCGGACGCGTCACGAGGAGCTCGGAATGGTCCGGAGGTAGATTGATATATAGGACGGATGGTTTCGGACCTTGGAAGTGTTTGGGGCATCCGTGCGTGGGGACCACGGAGGAGGTGGTCCAGGGGACTTCTCGAAGGGGGTGACATTTTGGGAGGCTTGATGGGCTAAGTGGAGCAGGGAACCAGCCCCTAGAAGTGGGCTGGTGCGGCCCCCACCCAGCCCATGTGCACCGAAAAAGGGGAAAGGGGGGAACCCTAACTTAGGTGGGCCTTAGGCCCACCCGGAGGGGTACGCCACCCCTCCTCCTTGCCACAGCCGCCACCACCCCCATCTGGGAGGGCTGCCGCACCTGCCTAGGGTGGGAACCCTAGGGGTGGCACCCCCTCTCCCCTTCCCCTATATATAGTGGGCACTTTTGGCCTTTGGAGGACACGGTTTTTCTCTCTCCCTCGGTGCAGCCTGCCCCTTCCTTCCTCTCTCATCTCTGCCGGCGCTTGGCGAAGCCTGCCGGGAGACCTCGTCTCTTCACCAACACCACGCCGTCGTGTTCTTGGTCTTCTTCCCCAACCTCTCCCTCCTCCCCTTCTTGGATCAAGGTGCGGGAGCGATCACCGGGCTGCACGTGGGTTGAACGCGGAGGTCTTAGTTGTTCGGCACTAGATCGCGGAGATATTGCGAGTACGACGACTCCACATCAACCGCGTTCTCGCGGCCTTCGCTCTTAGCGATCTTCAAAGGTACGAAAGATGCTCTGCACCCCTCTCTCGTTGCTGGTCTCTCCATAGGAAGATACCGAACATGCGTAGAAATTTTTTGAATTTATGCTACGTTACCCAACAACCGGCCGCGCTCACCCGACGGCAGCGGCGAGGAGGCGGCGGCGGCTCGCGGGGAGGGGACGGATTGGGGAGCCGCCCGGTCACCTCGCTCGGGGGAGGGCGACGCGGGGGCCTCGGGGAGAGTTTTCTTCCACCCGTTCCGGCGAGCAGTAATTGTTCATCTTTCCCGAAGGTGTGCATAGCCCCTTTGGCTGAGGAGGCTAGGTTCTTCGGCTTGGCGAGGATGGTCGACCCAAATACCACATGGTGAAGTGGGTCGGAAATCTGCCTGCCCGAAGGACCGAGGAGGGTTGAGCATCCGCTTGCATCTCGCCGTATGAACGTTGTCCTCATGCTTCGTTGGGTGTGGTGGATCTGGGAGATGGGGGTTTGTGGCTACAAGCTGATTGAGGCCAAGTACATGCGGGGTAAACCCCTCTTGGCCTGCTCGCACTCGGCTTTAGGTCCCAGTTTTAAAAATCTATCCAAGTCATCAAGGAGGATATTAGGTTGGCCTGCATGCAAGTCACCCGGCCAACCCATACATTGGCTACCCACCTGCCCGGAATAACAATCAAATCTACAGATCCTCTAGATGGACATGTCCAGTACCAAGTCACCACTAATTGCTCCAGACCATCCCTCCTTTGCTTGATCTATCAAACGACTCCATGAAGCTGCTCCGGCTCCGTAGCGTATAAAGCCAATCATTTAACATATCCATACATGCTATTAATAACAAGATTAGTTCCAACATGTGTTGCTAGGCAAAGCCCCACGGTGCCAAGAGCGGGAGGTGCTGTTCAGGGCCGCCTTATTGGAGAATTTTAGAGGTCTTGGAGAGCTAGTACGGTTGAAGAAGGAGATGACGGCTCGGTTGAAGATCTTCGTAAGGCGGTGCTCGGTTACGGTGTTGCTGAAGCCACCGTTGATCCGCCAATCAGTATATAGTGTGTTTCTATAAAGGCTATGCTTGGCTAGGCCATTGCAGAAGACCGCGTCGGCACATGGCCCTGGAGGGGACGATGAGTTCGATCTCGGAAGCGGCGTACGCCCTGGCTATGTGATGTTAATTTGTGAAGCGACACGCCCTAGCTATGTGATGTTAATTTCGTTCTTTTTCCATGCGCTTAGCGCGTAATCAAGTGGAGGTAGGTTAATTACCGCCTTGAGACACATTCAGGGCCGGCCCATAGGCAGGGGCAGCGTGGAGACTGCCCTGGTTTTATCATCACACTGATATTATTGTAGTACTATAGATTATATGATAGTTTAAGCGGATCACTTCGTGTCCCTCGGTCACATGTGCATCCTCTCACAGGAGTCGGGCGAACACGGACACGGCGCGGTGAAATCACTCGTCGTCCTAACTAGGGATGAAAACGGTCGGTAACGGTCGGTAAAATACCTCTACCGTTTTCATTTTCATATTTAACTGCTTGGGACGGAAGCAGCAGGGATATACCGGTAACGAAAACGAACGGGATAAATACGGTAATCGAAAACCGATACGATCCAGGGTCGGGTTAAGTCGAAATCGGACGGGAACCGGTATTTTTGTTCAGTAAAATCACACATGAAAACATATATTCAAAACTTAAAAACAAATATAAAAAATTGTAAACACGCTCCATGGGGTCGTAAGGAGCTGCAGTGCAGCGTGACGGTCGTGCCCCTCTCTAGAGATAATGAGCATTGCATGTCTAAGTTATAAAAAATTACCACATATTTTTTTGTCACACTTGTTGAAGTGCAGTTTATCTATCTTTATACATATATTTAAACTTTACTCTACGAATAATATAATCTATAGTACTACAATAATATCAGTGTTTTAGAGAATCATATAAATGAACAGTTAGACATGGTCTAAAGGACAATTGAGTATTTTGACAACAGGACTCTACAGTTTTATCTTTTAGTGTGCATGTGTTCTCCTTTTTTGCAAATAGCTTCACCTATATAATACTTCATCCATTTTATTAGTACATCCATTTAGGGTTTAGGGTTAATGGTTTTTATAGACTAATTTTTTTAGTACATCTATTTTATTCTATTTTAGCCTCTAAATTAAGAAAACTAAAACTCTATTTTAGTTTTTATTTAATAATTTAGATATAAAATAGAATAAAATAAAATGACTAAATTAAACAAATACCCTTTAAGAAATTAAAAAAACTAAGGAAACATTTTTCTTGTTTCGAGTAGATAATGCCAGCCTGTTAAACGCCGTCGACGAGTCTAACGGACACCAACCAGCGAACCAGCAGCGTCGCGTCGGGCCAAGCGAAGCGGAGACGGCACGGCATCTCTGTGTCGCTGCCTCTGGACCCCCTCTCGAGTTCCGCTCCACCGTTGGACTTGCTCCGCTGTCAGCATCCAGAAATTGCGTGGCGGGCAGCAGACGTGAGCCGGCTCGGCAGGCGGCCTCCTCCTCCTCTCACGGCCACGGCAGCTACGGGGGTCCTTTCCCACCGCTCCTTCGCTTTCCCTTCCTCGCCAGCCGTAATAAATAGACACCCCCTCCACCCTCTTTCCCCGACCTCGTGTTGTTCGGAGCGCACACACACACAACCAGATTTCCCCAAATCCCACACACAGCCAGATTTCCCAAATCCACCCGTCGGCACCTCCGCTTCAAGGTACGCCGCTCGTCCTCCCCCCCTCTCTACCTTCTCTAGATCGGCGTTCCGGTCCATGGTTAGGGCCGTGGTTCTACTTCTGTTCATGTTTGTGTTAGATCCGTGTTTGTGTTAGATCCGTGCTGCGTTCGTACACGGATGCGACCTGTACGTCGGACACGTTACGATTGCTAACTTGCCGGTGTTTCTCTTTTGGGGAATCACGGGATGGCTCTAGCCGTTAGCGAGACGGGATCGATTTCGTGATTTTTTTTGTTTCGTTGCATAGGGTTTGGTTTGCCCTTTTCCTTTATTTCAATATATGCCGTGCACTTGTTTGTCGGGTCATCTTTTCATGCTTTTTTTTGTCTTGGTTGTGATGATGTGGTACGGTTGGGCGGTCGTTCTAGATCGGAGTAAGTCTGTTTCAAACTTCTTTGGTGGATTTATTAATTTTGGATGCGTATGTGTGTGCCATACATATTCATAGTTACAATTGAAGATGATGGATGGAAATATCGATCTAGGATAGGTATACATGTTGATGCGGGTTTTCTTGATGCATATACAGAGATGCTTTTTGTTCGCTTGGTTGTGATGATGTGGTGTGGTTGGGCGGTACGTTCATTCGTTCTAGATCGGAGTAGAATCTTTGTTTCAAACTACAGTGTGGTATTGTTGCAGGCTGTATGTGTGTGCTCATATGTTTGCAGTTACCGAGTTTAAGATGGATGGAAATATCGATCTAGGATAGGTATACATGTTGATGTGGGTTTTTCTTGATGCATATACATGATGGCATATGCGACATCTATTCATATGCTCTAACCTTGAGTACCTATCTATTATAATAAACAAGTATGTTTTATAATTATTTTGATCTTGATATACTTGGATGATGGCATATGCAGCAGCTATATGTGGATTTTTTAGCCATTGCCTTCATACGCTATTTATTTGCTTGATGCTTGTTTCTTTTGTCGATGCTCACCCTGTTGTTTGGTGTTACTTACGCCGGGTCGACTCTAGAGGATCCGGGTAGTCGGAGTCCCTTCCATCACTACGAATCTTGGAGCGCCTTGCCCCTCTTTGATCGAGTTGCGTTTCCCGATCCTGCCAAGGACTTTGTCCCGGATCTAAACGACTATGAATCACCAACATTCGAAATAGATCTTCTCTCAAAACCCCTTGACTTTGACAACTTCCCCACATACTCTCTACCAACGGTGGATTCAACCAAGACTTTGTACTCCGAAGAACCACTTGTTTGCTTCGACTTTGACTTCGCGAACCCGGCTATCGAAAATTATATAACCACATCGTCGGGACTGTTGGACGCGAGTGCCAAGCCGACATCGCCCTTCCCACCTTCACACGGCCAAGCAAATGCCCATTCCCTAGTTGCAAGTCGGCCACGATCTTTGACGGACGGGACTTTAGGCGGCATTCACCAGGCAACACTTCAAGCGCTTTTTACGTCGCTACTCGAGAATGCCCTCGATCCAGCTCAAGACCTGCAAGAAGTCGGCACCAAAGGCTTTGCGACTCGCAAGGACCGTGCTCGGCATGAGTCTAAGCACAAACCAACAGTGCGGTGCCCTTGGCAAGACAAGGAAGGACAACAATGTACGAGGGGTCTTTAGCGGGGTGGATAACATGGGAGGGCTATGTGCGAGGAGAAGAACCATCTTTTTCAAAGATGACGGGAACTACGAGACCCTTGTTGAAGTCAAGTTCGAAGGTGACACCCTGGTGAATGAATCGGTAGGGCATTGACTTTAAGGAGGATGGAAACATTCTCGGCCACAAGGTTTGGAATACAACTATAACTCCCACAATGTGTACATCATGGTAGACAAGCAAAAGAATGGCATCAAGGTCAACTTCAAGATCAGACACAACATTGAGGATGGCCCCATGTTATCCACACGCTTTAAAGACCCTGAGACATTGTTGTCCTTCCTTGTCTTGCCAAGGGCACCGCGCGGTGGTTTTGTGCTTAGACTCATGCGAGCACGGTCCTTGCGAGTCGCAAAGCCTTTGGTGCCGACTTCTTGCGGGTCTTGGTGGCGGGAGCATTCGAGTAGCGACGAGAAAAAGCGCTTGAAGTGTTGCCGGTAATGCCGCCTAAAGTCCCGTCCGCTTTCAAAAGGCGCGAAAGCGACCTGGCAGGGAATGGGCATTTGCTTGGCGTGTGAAGGTGGGAAGGGCGATGGGCGGCAGCCTTTGCGTCCAACGGTCCAGAACGATGTGGTTATATAATTTTCGATAGCGGGTTCGCGAAGTCAAAGTCGAAGCAAACAAGTGGTTCTTCGGAGTACAAAGTCTTGGTTGAATCCACCGTTGGTAGAGTATGTGGAAGTTGTCAAAGTCATAAAGTTTCGAGAAGATCTATTTCGAATGTTGGTGATTCATAGTCGTTTAGATCCAGGACAAAGTCCTTCGCCGGGATCGAAGCACAACTCGATCAAAGAGAGCAAGCATTTCAAAATTCACCCAGTGATGGAAGGAGGCCGGCTACGTAGTCCTACTCTTCAAGATCGAGAGCCTGCGAAAACGCAAACAACAGAGTGAGCATCGACAAAAAAGCGATCGCAGCTATAGAGCTTGCGTATGAAAGCGAGACACAAAATCACCTGCTACCGCATGCTCACCATCATCCAAGTATATCAAGATCAAAATAATTATAAAACATGCTGACTATTATAATGGATAGGTACTCAGGTAGAAGTATATGGGTAAAGATGCTGCATATCGTATATGCATCAATAAAACACATCAACTGTGTATACTATCTAGATACGATATTTCCATCCATCTTAAACTCGTAATACTGAAGACATGACACACACATACGGACAAAATTAATAAATGCACCAGGTAGTTTGAAACAGTATTCTACTCCGATCTACAAACATGGTGACAACAATACCACATCATCACAACCAAACGGCAAAAGCATCTCACCATAGCCATTAAATCAGTAAAACCACATCAATAAATAACATGCCTATCACAGATCGATATTTCCATCCATCATCTTCAATTCGTAACTATGAGCCATCGGCACTACCTTACGAATACAAGAATTAATAAATCCACGAGCCAGACGAAACAGAATTCTCCGATGACGACCGCCCAACCGGTACATCATCACAACAAGACAAAAAAGCATGAAAGATGACCGACAAACAAGCACTCCTCCTCAACGGGGAAATAAAGGCCAAATAAACCCCATCACTAGCAGACAAAAATCGTGGGGAAGTCGATAAGGTCGCGAACGTGAGCCATCCCAGGATTCCCCAAAAAGAAACAACTGGCAAGTTAGCAATCAGAGGGCGTGTCTGAATGCCGAGTCGCCGCCCAGCAGCACGGATCCTAACACAGGCACGGTCTGCTACCAAACATGAACAGGAAAAGTGAAACCTCGGACCCTCAACCACCATGGACCGAGACGCCGATCTAGAGAGGCTGTAGAGAGAACGGCAGCGCGCGAGCGGTCTCGGCCGGGGCGGGGAAATACGGACGTGGGGCCCCGGGGGGGGCTCTGGTTGTGCGTGACGTGCGTCCTCCAGCAACACGGACGAAGAGGGTGTCTGGCCCCCGATGCCGGCGGGCGCGAAGAAGCCGAAGGAAAGTGGAAAATCCCCCGTAGCTACCGGCCCCACTAGGAGCCGCCTGCCGTGCCGGCTTCCGTCTGCCGCTCCGCCACACAGCCCCTCTGGATGCCGGCTGGCGGGCCTCAACGGTGGAAACCAGAAGGAGAATCCGAGGCAGCGACGAATGCGTGCCGCTCTAACCGCTGGCGACGCGCGCTATCCTACTGTTCAGCACGGGCCAATGATACGTTAGGCCTAGGTCGGCCGTTGCTGGGCTGGCATTATCTACCGAGAACAGAAAATGTTTCCTTCGTTTTTTAATTTCTTAAAGTATTTGTTTAATTTTTAGTCACTTTATTTTATTCTATTTTATATCTAAATTATTAAATAAAAAAACTAAAAAATAGAGTTTTAGTTTTTCTTAATTTAGAGGCTAAAAATAGAATAGATGTACTAAAAAATTGAACCATTAACCTAAACCCCCAAATAGATGTACTAATAAAATGAATGAAGTATTGCTATAGATGAAGCTATTTGCAAAAAAAGGAGAACACATGCACACTAAAAAGATAAAACTGTAAGTCCTGTTAAATCAAATACTCAATTAATCCTTTTAGACCATGTCTAACTGTTCATTTATATGATTACTCTAAAACACTGATATTGACAACAGTACTATGATTATATTATTCGTAAATTAAAGTTTAAATATATGTATAAAGATAGATAAACACACTTCAAACAGAGATATTGTTAAAAAAATATGTGGTAATTTTTATAACTAGGCATGCAATGCTCATTATCTGGGAGGGGGCACGGCCCGAGATCACCTTACTGCCGCTTCTGCGGGTTCCCATGGGCACTCAGACTATGTTGTTTTTAAGTTTTGAATATATATCTTTTCATGTGTGTTGAACAAAAATACCGGTTCCCGTCCGATTTCGACTTTAACCCGACCGGATCGTATCGATTTTCGTGCCGTATTTATCCGTTCGTTTCGTTACGTATATCCCGTTTTCGTTTCCGTCCATAAGTTAATATGAAAATGAAAACGGTGGGGTGTATTTTGACCGTTACGACGTTTTCATCCTAGTTAGGGCCGACAGTATTTCGACCGCGCCGTGTCATGTTCGCGGACTCCCGAAGAGGATGCACATGTGACCGGCGGGGTTGCGGGGATGATCGTTTAAACTATCGTCTTCGCTTCTTGATAGTTTAAACGGATCGCCGTGTCCCTCGGTCACATGTGCATCCTCACAGTCCGGCCGAACACGGACACGGCGCGGTCGAAATGCTCGTCGTCCTAACTAGGGATGAAAACGGTGATGCCGGTCGGTAAAATACCTCTGGATTTTCATTTTCATATTTAACTTGCGGGACGGAAACGAAGCAGGATATACCGGTAACGAAAACAGACGGGATAAATACGGTAATCGAAACCGATACGATCCGGTCGGGTTAAAGTCGAAATCGGACGGGAACCGGTATTTTTGTTCGGTAAAATCACACATGAAAACATATATTCAAAACTTAAAAACAAATATAAAAAATTGTAAACACGCTCCATGGGAATTCGTAAGGAGCTGCAGTGCAGCGTGACCAGTCGTGCCCCTCTCTAGAGATAATGAGCATTGCATGTCTAAGTTATAAAAATTACCACATATTTTTTTTGTCACACTTGTTTGAAGTGCAGTTTATCTATCTTTATACATATATTTAAACTTTACTCTACAGATAATATAATCTATAGTACTACAATAATATCAGTGTTTTAGAGAATCATATAAATGAACAGTTAGACATGGTCTAAAGGACAATTGAGTATTTTGACAACAGGACTCTACAGTTTTATCTTTTTAGTGTGCATGTGTTCTCCTTTTTTTTTGCAAATAGCTTCACCTATATAATACTTCATCCATTTTATTAGTACATCCATTTAGGGTTTAGGGTTAATGGTTTTTATAGACTAATTTTTTTAGTACATCTATTTTATTCTATTTTAGCCTCTAAATTAAGAAAACTAAAACTCTATTTTAGTTTTTTTATTTAATAATTTAGATATAAAATAGAATAAAATAAAGTGACTAAAAAATTAAACAAATACCCTTTAAGAAATTAAAAAAACTAAGGAAACATTTTCTTGTTTCGAGTAGATAATGCCAGCTGTTCGCCGTCGACGAGTCACAACGGACACCAACCGGCGAACCGAGCGGCGTCGCGTCGGGCCAAGCGAGCGAGACGGCACGGCATCTCGTCGCTGCCTCTGGACCCCTCTCGAGAGTTCCCGCTCCACCGTTGGACTTGCTCGCTGTCGGCATCCAGAAATTGCGTGGCGGGCGACGGGCGTGAGCCGGCACAGCAGGCGGCCTCCTCCTCCTCTCACGGCACGGCAGCTACGGGGATTCCTTTCCCACCGCTCCTTCGCTTTCCTTCCTCGCCCGCCGTAATAAATGAACACCCCCTCCACACCCTCTTTCCCCAACCTCGTGTTGTTCGGAGCGCACACACACACAACCAGATTTCCCCCAAATCCCACACACAACCAGATTTCCACCAAATCCACCCGTCAGCACCTCCGCCAAGGTACGCCGCTCGTCCTCCCCCCCCTCTCTGCCGCTATCTGATGGTGATTGCGGTCCAGCGGTTGGGAGCCCGGTAGTTCTACTTCTGTTCATGTTTGTGTTAGATCCGTGTTTGTGTTAGACACGTTCGTACACGGATGCGACCTGTACGTCAGACACGTTCTGATTGCTAACTTGCCAGTGTTTCTCTTTGGGGAATCCTGGGATGGCTCTAGCCAGATTTCATGAGACGGGATCGATTTCATGATTTTTTTTTGTTTCGTTGCATAGGGTTTGGTTTGCGCTTTCTTTATTTCAATATATGCCGTGCACTTGTTTGTCGGGTCATCTTTTCATGCTTTTTTTTGTCTTGGTTGTGATGATGTGTAGTGCAGTTGGACGGTCGTTCTAGATCGGAGTAGAATTCTGTTTCAAACTACCTGGTGGATTTATTAATTTTGGATACGTATGTGTGTGCCATACATATTCATAGTTACGAATTGAAGATGATGGATGGAAATATCGATCTAGGATAGGTATACATGTTGATGCGGGTTTTACGATGCATATACAGAGATGCTTTTTGTTCGCTTGGTTGTGATGATGTGGTGTGGTTGGGCGTGCGTTCATTCGTTCCTAGATCGGAGTAGAATACTGTTTCAAACTACACAGTTGTATTTATTAATTTTGGAACTGTATGTGTGTATCATACATCTTCATAGTTACGAGTTTAAGATGGATGGAAATATCGATCTAGGATAGGTATACATGTTGATGTGGGTTTTGCGGTACATATACATGATGGCATATGCAGCATCTATTCATATGCTCTAACCTTGAGTACCTATCTATTATAATAAACAAGTATGTTTTATAATTATTTTGATCTTGATATACTTGGATGATGGCATATGCGGCGGCTATATGTGGATTTTTTAGCCACTGCCTTCATACGCTATTTATTTGCTTAGTGCTTGTTTCTTTTGTCGATGCTCACCACATTTGTTTGGTGTTATACACGAGGTCGACTCTAGAGGATCCCCGGGTAGTCGGTCCCTTCCATCGCTACGAATCTTGGAGCGCCTTGCCCCTCTTTGATCGAGTTGCGTTTCCCGATCTTCCAAGGACTTTGTCCCGGATCTAAACGACTATGAATCACCAACATTCGAAATAGATCTTCTCTCGAAACTTATGACTTTGACAACTTCCCCACATACTCTCTACCAACGGTGGATTCAACCAAGACTTTGTACTCGAAGAACCACTTGTTTAGCCGACTTTGACTTCGCGAACCCGGCTATCGAAAATTATATAACCACATCGTCGGGACGGTGGACGCGGTGCCAAGCCCGGCTTATCGCCCTTCCCACCTTCACACGGCCAAGCAAATGCCCATTCCCTAGTTGCAAGTCGGCCACGAGTCTTTGAAAGCGGACGGGACTTTAGGCGGCATTACCGGCAACACTTCAAGCGCTTTTTCGTCGCTACTCGGGGAATGCCTCCCGGTCGAAGCAGACACCTGCAAGTCGGCACAAAGGCTTGCGACTCGCAAGGACGATGCTCGGCATGATGCAACTTCTCCAACGGTCTGATTGCCCTTGGCAAGACAAGGAAGGACAACAATGTACGAGGGTCTTTAGCGGGGTGGATAACATGGGAGGGCTATGTGCGAGGAGAGAACCATCTTTTCCAAAGAGATGACGGGAACTACAAGACCCGCGCTGAAGTCAAGTTCGAAGGTGACACCACAGTGAATAGAATCGAGTTTGAGGGCATTGACTTTAAGGAGGATGGAAACATTCTCGGCCACAAGCTGGAATACAACCATAACTCCCACAATGTGTACATCATGGCCGACAAGCAAAAGAATGGCATCAAGGTCAACTTCAGATCAGACACAGCATTGAGGATGGCCCCATGTTATCCACTGCTAAAGACCCTCGGACATTGTTGTCCTTCCTTGTCTTGCCAAGGGCACCGCACGCTGTTGGTTTGTGCTTAGACTCATGCCGAGCACGGTCCTTGCGAGTCGCAAAGCCTTTGGTGCACGACTTCTTGCGAGGTCTTGAGGGCGGCGAGGGGCATTCGGTAGCGACGGAAAAGCGCTTGAAGTGTTGCGGGTAATGCGCTAAAGTCCGTCCGCTTTCCAAGGCGTGGCCGACTTGCAACTAGGGAATGGGCATTTGCTTGGCCGTGTGGAAGAGCGTGGCGGCAGCCCCTTTGCGTCAACGATCCCAGAACGATGTGGTTATATAATTTTCGATAGCTAAGTTCGCAGTCAGAAGTCGAGCTCAGCAAGTGGTTCTTCGAATTACAAAGTCTTGGTTAGATCCCTTAGATTGGTAGAGAGTATGTGGGGAAGTTGTCAAAGTCATAAGTTTGAGAGAAGATCTATTTCGAATGTTGGTGATTCATAGTACGTTAAATCTGGGACAAAGTCACTGGCAGGATGAAATAAAAGAGCAATATCTCAAAATTCGTGATAACTGTCAAGCAGATCGTTCAAACATTTGGCAATAAAGTTTCTTAGATTGAATCCTGTTGCCGGTCTTGCGATGATTATCATATAATTTCTGTTGAATTACGTTAAGCATGTAATAATTAACATGTAATGCATGACGTTATTTATGAGATGGGTTTTTATGATTAGAGTCCGCAATTATACATTTAATACGCGATAGAAAACAAAATATAGCGCGCAAACTAGGATAAATTATCGCGCGCGGTGTCATCTATGTTACTAGATCGACGCTGAGCTCGAATTCTAGTGGAGGCCATATTGCAGTCATCCCGAATTAGAAAATACGGTAACGAAACGGGATCATCCCGATTAAAAACGGGATCCCGGTGAAACGGTCGGGAAACTAGCTCTACCGTTTCCGTTTCCGTTTACCGTTTTGTATATCCCGTTTCCGTTCCGTTTTCGTTTTTACCTCCGGTTCGAAATCGATCGGGATAAAACTAACAAAATCGGTTATATACGATAACGGTCGGTACGGATTTTCCCATCCGCTTTCATCCCTGGTCCTAACGTCGACCGTTTCCATCCATCTAGCTTCTCCTTGAACCACATGAGCGCTAAGCTTGAATTCTTGCGGAGATTCGGGTCAAGGGGAAGCCAGCTTGCCACCACCCCACGTCAGCAAATACGGAGGCGCGAAGTTTGACGGCGTCACCCGGTCCTAACGGCGACCAACAAACCAGCCAGAAGAAATTACAGTAAATAAGTGCACTTTGATCCACCTTTTATTACCTAAGTCTCAATTTGGATCACCCTTAAACCTATCTTTTCAATTTGGGCCGGGTTGTGGTTTGGACTACCATGAACAACTTTTCGTCATGTCTAACCCCTTTCAGCAAACATATGAACCATATATAGAGGAGATCGGCCGTATACTAGAGCTGATGTGTTTAAGGTCGTTGATTACTTTTTGAGAAAAAAAAATCCAAATCGCAACAATAGCAAATTTATCTGGTTCAAAGTGAAAAGATATGTTTAAAGGTAGTCCAAAGTAAAACTTATAGATAATAAAATGTGGTCCAAAGCGTAATTCACTCAAAATCAACGTGATGTGTACCAAACGGAACAAAACCAGCATCTTCTCAGCTTCCCAACCGCTCGCTCGCCCGCCTCGTATTCCGTCACGGTGGTTTCAGCGTGGCGGGATTCTCAAGCAGACGGTGATGTCACGGCACCGGGACTCCTCCACCGCAACCGCCATAAATACCAGCCCCCTCATCTCCTCTCCTCGCATCAGCTCCACCCCGAAAAATTTCTCCCCAATCTCGCGAGGCTCTCGTCGTCGAATCGAATCCTCTCGCGTCCTCAGGTACGCTGCTTCTCACTCTCTCCTTTTCGTTTCGATTCGATTTCGGACGGGTGAGGTTGTTTTGTTGCTAGATCCGATTGGTGGTTAGGGTTGTCGATGTGATTATCGTGAGATGTTTAGGGGTTGTAGATCTGATGGTTGTGATTTGGGCACAGTTGGTTCGATAGGTGGAACGTGGTTAGGTTTTGGGATTGGATGTTGGTTCTGATGATTGGGGGAATTTTTACGGTTAGATGAATTGTTGGATGATTCGATTGGGGAAATCGGTGTAGATCTGTTGAGGTGGCTGGAACTAGTCATGCCTGAGTGATTGGTGCGATTTGTAGCGTGTTCCATCTTGTAGGCCTTGTTGCGAGCATGTTCAGATCTACTGTTCCGCTCTTGATTGAGTTATTGGTGCCTTGGGTTGGTGCAAACACAGGCTTTAATATGTTATATCTGTTTTGTGTTTGATGTAGATCTGTAGGGTAGTTCTTCTTAGACATGGTTCAATTATGTAGCTTGTGCGTTTCGATTTGATTTCATATGTTCACAGATTAGATAATGATGAACTCTTTTAATTAATTGTCAATAGTGAAATAGGAAGTCTTGTCACTATATCTGTCATAATGATCTCATGTTACTATCTGCCAGTAATTTATGCTAAGAACTATATTAGAATATCATGTTACAATCTGTAGTAATATCATGTTACAATCTGTAGTTCATCTATATAATCTATTGTGGTAATTTCTTTTTACTATCTGTGTGAAGATTATTGCCACTAGTTCATTCTACTTATTTCTGAAGTTCAGGATACGTGTGCTGTTACTACCTATCTGAATACATGTGTGATGTGCCTGTTACTATCTTTTTGAATACATGTATGTTCTGTTGGAATATGTTTGCTGTTTGATCCGTTGTTGTGTCCTTAATCTTGTGCTAGTTCTTACCCTATCTGTTTGGTGATTATTTCTTGCAGATGCAGATCTTTGTCAAAACATTGACCGGCAAGACTATCTACCCTGGAGGTGGAGTCCTCTGACACCATCGACAATGTCAAGGCTAAGATCCAAGATAAGGAGGGCATCCCCCCGGACCAGCAGCGATTGATCTTCGCTGGCAAGCAGCTCGAAGATAGCAGGACCCTTGCTGACTACAACATCCAGAAGGAGTCCACCCTTCTACCTTGTCCTCCGCCTCCGTGGTGGAATGAAAAACCCCAGACTCACCGCGACGTCTGTCGAGAAGTTTCTGATCCGAAAAGTTCGACAGCGTGTCCGACCTGATGCAGCTCTCGGAGGGCGAAGAATCTCGTGCTCAGCTTCGATGTAGGAGGGCGTGGATATGTCCTGCGGGTAAATAGCTGCGCCGATGGTTTCTACAAAGATCGTTATGTTTATCGGCACTTTGCATCGGCCGCGCTCCCGATTCCGGAAGTGCTTGACATTGGGGAGTTTAGCGAGAGCCTGACCTATTGCATCCCGCCGTTCACAGGGGTGTCACGTTGCAAGACCTGCCTGAAACCGAACTGCCCGCTGTTCTACAACCGGTCGCGGAGGCTATATGGATGCGATCGCTGCGGCCGATCTTAGCCAGACGAGCGGGTTCGGCCCATTCGGACCGCAAGGAATCGGTCAATACACTACATGGCGTGATTTCATATGCGCGATTGCTGATCCCCATGTGTATCACTGGCAAACTGTGATGGACGACACCGTCAGTGCGTCCGTCGCGCGAGCTCTCGATGAGCTGATGCTTTGGGCCGAGGACTGCCCCGAAGTCCGGCACCTCGTGCACGCGGATTTCGGCTCCAACAATGTCCTGACGGACAATGGCGCATAACGCAGGTCGTGGGCCTGGAGCGAGGCGATGTTGGGGATTCCCAATACGAGGTCGCCAACATCTTCTTCTGGAGGCCGTGGTTGGCTTGTATGGAGCAGCAGACGCGCTACTTCGAGCGGGAGGCATCCGGAGCTTGCAGGATCGCCACGACTCCGGGCGTATATGCTCCGCATTGGTCTTGACCAACTCTATCAGAGCTTGGTTGACGGCAATTTCGATGATGCAGCTTGGGCGCAGGGTCGATGCGACGCAATCGTCCGATCCGGAGCCGGGACTGTCGGGCGTACACAAATCGCCGCGGAAACAGCGACCGTCTGGACCGATGGCTGTGTGAAAGTACTCGCCGATGGTGACAGCCCCAGCACTCGTCCGAGGGCAAAGAAATAGGCTTTCAGCCAGTTTGGTGGAGCTGCCGATGTGCCTGGTCGTCCCGAGCCTCTGTTCGTCAAGTATTTGTGGTGCTGATGTCTGCTATTGTCTGGTTTAATGGACCATCGAGTCCGTATGATATGTTAGTTTTATGAAACAGTTTCCTGTGGGACAGCAGTATGCTTTATGAATAAGTTGGATTTGAACCTAAATATGTGCTCAATTTGCTCATTTGCATCTCATTCCTGTTGATGTTTTATCTGAGTTGCAAGTTTGAAAATGCTGCATATTCTTAGTAAATCGTCATTTACTTTTATCTTAATGAGCTTTGCAATGGCCTATGGGATATAAAAGATTATTCTGGAGGGAAGTGATGCTGGAAGGACTATGCTGTCCCTGATTTATATTTGGAGCCACTATGAGCATTTGGGCTTTCTTTTCAGAACGCTGTAGGCGTGTGTTGAAATCTTTGCGACATTCAATTTGATATATGATTCGAGGTAATTGGGCTTTAATTTGTCATCTCATGTAACATCTTTTTGTTTCTTAGCTGCTTGATTTCTCTATTTCGTAGCATTGGAAGATAATAGTAGAATGATGATATACTCCAATACTTGCAATTTCAAAACCGTTAGAAAGAAGGAAAATCACGGCCGCTGGATCGAATTCGGCACAGAGTGTTCAGCCCCAGTACATTAAAAACATCCGCAATGTGTTATTAAGTTGTCTAAGCGTCAATTTGTTTACACCACAATATATCCCGGAGTAGTATTACATATATGTATTGATATATTTTATTTAGATCCAGAAAAAAAGGAAGAAGAAAAAAGGCCCAAACCCACAACGTAGGTAGCTTTCCCGGATCGAAAGAATCGTTCCCTAGTTTACTATGCTGTGAGCACTCCATCGATCTCTTACGGTCATCTCGCCTCCTCGACTCCTAAAAAATCTTGCCTGCGGCTCCTCCCCCAAATTCTTCAATCTGGAGAACGGCGTAGTCGCTCCCGTTCACTTTCAAATTCGAGATGGTGCTCCCGTTGCATCCACTAATGGAGACCGATGGTGCTATTCCCTTCCCCCGATCACTTCTTATAGCATCCTCGGTGTTCCCCATCAATAGACGTTTGAAGCATCACACGCATCGGAGATGGTCGTCTCCCTCCTTAAACCTAATCAGAGTATTATTTTCATTACATTTGTTAAACAGCCGCCTCTTCGCTTCTGGTTATCCACTGACATCTTCCAATTTTCTCTTCCTAGTTTCTACTCTTTTGTTGGCCTGTTCTTCGAACCTTCATTGGATCCGGAAACTACATCGACAATTCGACATCAAGCCATATGCCCATACCCAAATGCTTTTGTTCCTTGTATTAACAGTTTTACGTAATTATTTGGTGACATGTGTTCTAAGAAATGTTTATCTGTGAAAATAAAGTGAAAAAAACATAAATGGATTTACGTTGCTATCAGTTTGACTATCGTTTTCATACTTAGAAGAGCAAGATGATAGTCTTTTGTGCAACTTAAAGAAGCTCTTTTGTAGAACTATATTATTTCCATACTATTATGATGTAGTTCTCTCGACCATCAATCCTATGCTATGTACAGAATTACTTTAGAAATATAATATATTCTTAATTTTTATTTGTATACGGGGCCTGAATTTGTCGTCTCTTCCCAAGGCTACAGAATATGTATGGACCGGCCACAGGCCACATTATTGTTTCTTCTGATTAGATGTGACGTTTCGTGTGTGTGCGTGCGTGCGTGTGTGTGTGTGTGTGTGGGTGTGGGTGTGTAGGGGGGGGGGGGGCTTAGCTAGGCCAGGCTATAATCACATGCCCGATGCTAGAATACTTTGTGGCTAAGAATAAGCTGCACCAGGAGATTTCCTACGGGTGGAGGGTAAGGAGCCGATGGAAGCTTGGTGAAGAACCAAAGCTTGACATGCTTACACCTCGGAGCTCTGGCGAAGTGCTCGACGAAGAACCGCAGAGCTATGACCCAAAACCATTGTTTTAAATAATGGGTATAGTCATACATAGCGCTTGTTGTCCCTTTTTATGCGTAGTCTTAGTTAAATGGTATAAAGCGTATAAATAGAGCAGTATGCTTAGCTTCATTCCATTATACGTGATTTAAATGCCGCCGTTATTTATTAAATGCATTGTTAAAACATTTAAAACCCAAGCCCACGAAAAATTCAACGCCCCTATCAAGCACGGATGAGCTCCGTAAAAGGTATGGTCTTGTTGTGAGTACCAAGTGACCACCATGATTCTGCTGCTGTTGTACCCAGCAAATTAGAAGGGAAAGTGTTGAACAAATCCAACATTTGAGCTTGTCACGCATCAGAGAAGCAGGCATGAGAAGATGCCCACTGAACACCATGATCTTGTGCTGTTGTACCGTGTTAGGTCACAGTTGGACTATACTTTTAGCAGAGAACACACACCTCATCGGGATGAAAGTTCCAACGGATGCCCTTGTCAGAATCTTACATGCTATGAGACGATGTTGAAGGTGGTTTAATTTCCATCATTGATGCCAAGGCACGGCCATAATTTGGTTGGATCTTGTAGAATGATAAGATGTGATTCATACGTTTTGTACCTTGTATGGCAAGTTACTGTATTACTACATCGAATTAAATTTTTGGCAAAAAAAGAGACTGGACCTTAATTCAGCAAAATGTTTGTTTTGAATTTTATTTTTGTTTCTAGTAAAATATAGAATTTTCTTTTGACTTAGGAAAAAATAGTTATGCCGCCGATTACAAGTAATAATATGAATTTGAGGTTTTTAATTTAAGATATTCGGTTGTAAAATATATTATTTAAGGCGTGAGCGGCTAGTGTCCGTGGACGCGTCCGCAGGTGTTTGAAGAATGGGATTTGCATGTTAGCGTTGTAGATGCTCTAACAACCTTTCAATTCAACTAAGCACCTAATACCACAACATGCATGCATCCAACGGAATCAGGAATCAGTGAACATCAACAACTGCGGTGGTGTGTGTGTCTCGGGCCGGTTATGTAGAGAGGATTGATTTCATGGAACGCTTGGGATGAATGGATGCAAAAGAAAAGAAAAAGTTAAGAAAAAGATCTCCTACTAGCAAACTGTGCAGATCATTTCTTTCGTGCCAAGACACACATGTTTTGTCTTTTTCAATGAATTCGAAGCTTTATTCAACGAAGCAGTAACCGGTTACAACCAGACCGTAGGCAACTGATAAGGAAATCGGGGCTGTGGTCTAGCCAGCTTTCCGTCCCATCTAGTGAGCACACGTGGGCACACTCGTGAGCCGAAACATTAAGTTCCTTACTAACATGCCGAGGAAAAGGAAACAAAACTAGGTATAATAGCCCCTAATTCTCTTAAGATAGACGTCACAATTGATTTTGTGGTGATGTGCGAATTCCAAAGGTCGATCAACTCGAAGCAATCAACTTGTATTATCACACATGTGAGTATCTCCACAGCTGAGAAGAATGCTCCCTCCCATAACGCCAGCAGTTCAGAGGAACAAAGGATTTGAAACTCCCGGTGATGGCTTGCACCAGGCTGCCAAGAATGCCAGGTGCGAGTGAGCTACTCCTCCTGCTCCCGCTGAGGGGCATCTCGGCTACCGATCCATCGGCATTGATAGTGATCCATCCCACCTCCGGTGAATGCCAGGATTGTGCTGGTAGAACTTACTCGCCGTGTCGGAAGCTTGAGGAGTGCTAGGGTTTCATGCACCCATTTGATGGCCAGAACCGGGTCATACCCCATCTTTATCATGAGTCCATATACTACGAGAAGACCAAATGGCATGCATGATAAATTCTGATCTTGCATCTAATAGCATCTGCAGAGAATTGCTGGTCCAGCAAAATGTCACGCGCCCAAGTGTGTGGTCAAGACCTAGGTAGATTCAGCTCGAACCAGTCTTTGGCGGCAATCCAAAACTTCTTAGCATGTGTACAAGAGATGAGCGCATGCATAGAGTATCATCCATGGCCTGACATATGTCACACCCACCAATCGGTTTAATATGACGATACTTCAGGGTCATTTAGTCTGTTGGGGATGCCATGAAGAACCCTCCAACCAGAACACTCAAACCTTTGGTACAACATGTAGTTTCCATGGTTCCACCTTTGTTTGTTTGAAGATGAAGTGTCCGTGATCCTTCCTTCCTCTAAATTACTAAGCCCATTGCGAGGTCGCGAAGAGGACGATACGCCGATTTAACAGTATAATGACCCAACTTTTCCAGAGCCCATGCCAAAGTATCATCATCCCCAGCCGGATTCAAGGTATGTTCAAAATCGCCTCCGCATCTGGGTGAAGAAAGTTTTGTCGGTCCAGATCCACATCCCAACTTCCTGTATAAGCATCAATCAAATCCCAAACCCTTTTCCAAAGGTGCGTTACCAATCAAGCCCATGGGTTTCAACGTTGCTGTATTTGGATCCACTGGTCTGTCCATATAGAAACAGAGGAGCCGTCTTCAATTCTGTGAATAAGTGCATTATCCAAAGCTTTTCTGCCGCTTTGTAACGCTCTCGGAGCAGGTAGATGATGCTCGACTGGGGATCGTTGCATGCAGAAAATCAGTATATGGGTAGTACTTTCCCTTCAGTACACGCGCACATAAACTGTTGGGGTGTGTCAACAACTGCCAGTCCTGTTTGCCTAACATGGCAAGATTAAATAGTTCAGGGTCCTAAAGCCATCCCTCCCTTTGATCTTCGACTCAGTCAATTTCTTCCACGATAGACAATGAAGTGCTCTCTTGTAAATAGAATTGCCCCACCAATGTTGGCCCATTGGTGGGGTGATTTTTGCAAACTTGTTTTGTCAACAAGAAACAACTCATAATGTATGTCGGTATAGCCTCGGCAACAGATTTAAGCAAAACCTTCCGTCCCGCACAGGCTAGGTTCCTCACCGACCAACCACATATTTTGCCATGCACCCGCTCAATAATAAGAGAGAATGTGCCGTTGGTGGGTCTTCCAATTGCAGTAGGTAGCTCCCAGGTAGCGTTCTGAAAACGCTTCCACGAGAATCCCAAGAGCAGATTTTAATGTCCCTTTTAAAGGGCTGACCTGTACTGAACAAGAGAATGAACTTTTTCTCGGTTCACAGCCGGACCTGAGCAGTCCTCATAAATCCGGAGAATGTCATTGACTCTAGGTTTTATACAGTGATTTGGTTGGATCTTGTAGAATGATAAGAAGGGATTTGTATATCTTGTATCTTATGTAGCAAGTTACCTTGTTACACTACAATCATCGAATTAAACTTTCGATGACAAAAGGACTGGACCTTAATTCAGAAAAATGTTAAATTTCAATTTTGTTTTCAAGTGTATTATAGAAAAAAATTATTTGGCAAAAGTATTGTAGATTTTTATTTTGACTTAGAAAGAAGTTATAACTGATGAGGCCACCAAGAGGGATAAAATCATTAGTGATGGTGAAAACTGAGTAAGAGATAAGTAAATATGGCATCCGACTAGAAAACCACTCACGGCAGCAGAACAAGATTTTACCATAAGTAAGACTGCGCATCATCAGATGAGAAAACCACGTGAAAACTCAAGCCAGCAACACGGTATTTTTCCTTTTCCGAACACACGTACATTCATTCAAGGCTCTAATAGAATTTTTGGATGAAGCAGGCTACAACAATCAGTAGCCTTACAGATGGGGCTCATGTACTCCAACACGCGAACCAACCTGTTGATGGACGGGACCGGCCCAGCCGCCGCTGAAGTTGTTGGTTGTAGAGGCTCATCCCCACCCGGAACGCTACCGGAGGTACATCCATACTGAGTTGGTGAGGTTTTGCAACAGCACGGTGGCGACTAGCCTACTACAGATCGTGCTTTGTCGTTCCGCTGCTTCCCAGTGGCTCCATTGGCCTTGGTTGATGAGGACAGCGACTGCGGTGGCGTGTGTCTCGGGCTGGTTATGTAAGAGGATTGATTTCATGGAACATATATATGAGACGAATGGATGCAAACAAGCAAAAAAGGTAAGAAGATCCTACTAACAAACTGCAAGATCATTTCTTTGGTGCCAAGGCGCGCCAGTGATTTGGTTTTCTTTGGTGCCAAGGCGCGCTAGTGAGTTGGTTGGATCTTGTGCAATGATAAGATGGGATCTGTACACCTTGTATCTTGTACTATAGCAAGTTACCTTACCGTTATACGTATGGTTAGATCAAAATGGAGGAAACAGAGTTTAGGATTTAGCCGTTTTGATTCGGCATCAAAATGCATGGTTCGAGCTATGTACGCGATTCGGCATCAGGCTTGTTCTTCCTTTATGCTTTAAGGCAGGTTAGAAGTATACTCGCCGGTCGTCAATTATGGATTTCAACAAGCCTGATTCGACCGTCCAAGAGGAATAAAATCATTACTGATATGGAGAAAACTTGAGCAAGCTAGATAAACTTGAGCAAGCCTTCTCCTCAACATTGCATCCAATTAAAGAACCACTCAAGCCAGCCAAACGAGATTAATTATATGTAACACTAGGCAGAAAACCACGTCTATTAAACAAAAGAAAAAAAGAGATCGAGAACCATGTACTCCCTCCGTGTAAAATTCTTATCTTAAGGTTCGTATAGAAATATAGATGTAATAAATGTTTAAACGTGAGCGTATCTAACAAATTTAAGAAAGAATTTTGGGACGGAGGGAGTATGTATTTCTTTTTGCGAGCACATGTCCATTCATTCAAGACTGATAATTTTTGGGGTGAAGGCTACAACAATCAATAGCCTCCTTACACAAAGACAGCCACCAAAACGGAAGCAACCAAATCACCACATGTTGCTTTTTATTAAGCGCACACGAGATCACATAATAACGAACTAAACTTAAATCGTCCCTAGTAAAAATAAAAATGCACCTAAACTCTCCTAATTAAGATTCAACCCGGACATGACGTCGATCGATCGTGACGCCAAGATCGATGACGCTATCTAGTTGGTGCCCTTGACGATAAGCTCGAGGGCGGTGGCGGTGGTGGGCAGCGCTGGAATGGCTCCTTCTTGGTGGTGCGATGGCTCCGCAAGCGTTTGAGAATTGCAACACCTCCCTCAGCTTGGCCTCATTCTGTTCAGTCAAAAGAGAACTTCATAATTAGCAACATGCTCACTCACTCGATCATACTGGAATTCAATCATGCATCTGATAATATATCTTCTTACGTAGAAGATGAGTCGTCCTTGGCGACGTTGAGAAGAGGATCAACGAAGGCATCGCCGGCGCCGGTGGTATCAACCGTGTTGACGGCGTACCCAGCCACGGATCCTTGAAGTCCTTGGTGAAGTACCTGCACCCCTTCTCGCCGTCGGTGACGACGAGCAGCTTGAAGACCTCGAGCCAGAGGAGAGGACGTTCTTCTCGTCGTAGGCGTCGCCCTGGGTGAGGAAGGCCACCTCGTCGTCGCTCACCTTGATGAAGTCGGCCTCCTTCCAGATGCTCATGATGCCGTCGCGGGCAGCCTGGGCGGAGGGCCAGAGCAGGGCGCGTTGGGGTCGTAGGAGCAGGATGCCGGCAGACTTGGCGGCACGCATGGCGGCGACGTGGGCGGAGCGGCATGGCTCGGTGATGAGCGAGATGGAGCCGTAGTGGAAGATGCGGGCACGGCGGATGAGGTCGAGGTTGAGCTCCGCCTCCGTGAGGAGCATGTCGGCGGACGGGTTGCGGTAGAACATGAACTCACGCTCGCCGTTGGTCTTGAGTGTGACGAAGGCCAGCGCGGTGCGCGTGCTGGTCGAAGAGCGGCCCTCGGCGTTCACGCCGTTCTGCTTCAGGATGTCCACCAGCATGTGCCCGAACTCGTCGTCGCCGAACTGATCGAAAAAGAAAACATCATCATCCGATTAGAATTTCAAATGCCGCCTAACCTATGGCGACACAAACCTTCGGCATGATCAGATCCGGACGGGAGCACAAAGGGTCTGAGCCTACCGAGGCGGATCTAAATGCACGCGCCCGGATGGCAACGAACGCAAGGACATGAGAATCATCATCTGTTATCAGGGACGGCGATTGATCCGTGTACGTACCTTGCCGACGAAGGCGGACGAGCCGCCAAGCTTAGAGATGGCGCATGCGACGTTGGCGGGCGCGCCTCCGGTTTGCCCTTGACGAAACCTCCGGACTCGGCGAGGAGACGCCGGCCACGTCCGGCACAAAGTCGATCAGCATCTCACGAAGGAGACGACGAGGCCGGATGCCGCACTTTGCGGCAGTGGGAGGAACAACATCACCTAGAGCGCCATTGGAATTGACTAAACGAACGAGAACGCGAAGGGATGAACCGATCAAGCTGGGAGAAGGTGCTGATTGACTTGGACTCGGGAGCCGGGGGGACGGCTTATAAAGGGCCGCCTTGGGGGTTGGCTTGACCGAAGGATCCGGTTAGTTATCAAATCTTATCCTACTTTTGTTACAATCCGCTAATCAGTTTAGGTTAGGATAACTCCTTTTTTGTTGCGAACCGAGGTTGGATAATCATGCTGCCATGAGTAATTGTTCGGTTCATGAAGGATTTTGTTTGGGTATTATACTACACTAGTAAGGCCCTGTTTGGAACCATCCAAATTATATAATCGTTTTATAATCTATTATGTCTTCAAACAGGACATATTATTACAGTTTATAAAAACTAGATGGCTAGATTATTAAAGCGAAAGCTGTAATCAATATGGGGGGGGGAAATCAGATTATGGATTACTAATAAATACCCTTGAAACCTTGGAGATAATTACCTACTGACACCGTCACGGTATCGTCGTGTTGCAGATTCTTCTGTTACAACTAAAAAGCGAGAGGGCAGACATGTCATTGTACAATATAAAACCTGAATTATAGTTTATATAATCTGGTCTTCAGCAATACCCACCTAGATTATTTTTATAAATCAGATTATATAATCTATCTTCATAATCCAGATTATTTACAATCCTATTGTGGTTCCAAACTGAGTTCTATAACATGCTGCATTTGCTCGTCTAAAAAAAATGAATTCATATGATATCATATTAAAATAATTTTTGATTTTTTCCAGACGGGTACAAAATGTTATTATGTCACTCAAATTGTATTTTCAAAATTGTGACATATTTGTGATCCAACACACGTATCATAATCTATAGTAAAACATATAGAGTTTTTATCGAGTCATTTATCGGTGTCTTATATAAAACAATATGAGAACGTGCGTAACATACGATCCGAAACATGTCTCCGCACAAATTCACGATCACATAATAATTGTACTATAACTGTTAGAGAAATATATTTTAAAAACCAGGACTAAGCACGTCTTGATGGTCCATCTTTTGAAGCAATGAAAGTCCATCTATAGATGCGATCTATGATGCTTCCTTCAATGTAGCATGGGATATTGTTTTTAAGATTCATTTTCTTCATACTTTGAAATATTGCATAAAATCGCTTCCTCGGTTCAAAACAATCCTACATATGAATTAGACAACATTGTTATAACAAGTTTGTATGTGCAATGTGTATAAAAATAAGCATGGTGTAAATACTCACACAGGGTATGAAGAATATAGCTTTCCATCGCACAATGAGTGCATGTAGTTAAAAACCAAATACCCTGACAAGGCATTTTACATGTATGAAATAAATACATAATGTGCGTTGGATGAATGAATTATTTAATATTATAATGCATTAAAAATATTCGTACTAGTGTGAGTTTGTTGGAACAATAGGAACTATACATTCCCATTTAGAGATATAGTCCTTGAATGCAAGGGTTTGCAACTTGGAGTGCGAGGTTAGGTTTTGACTACGGTTTTAAGTATATTCTTTCCCAAAGTAGGTATTGGTATCGGATCAAAAATTGAGATAACTTTTTTATCTTGATCCAAGACAAACAATAGAAACAACCCAATAATATCCCAAAGGCAATAAAATCTGCAATGATGCATACGTTTATAAGTACATGAAATATTAATTAAAACCAAATAAACCAATGTTATAAACGAAAAATTAAAATCTTATCCTCTGCTTTGTGAGATATGATACTCATTGATATTGAGACCTTTATGAAACAATGTTTGAATCATAGTGTTGATGAACGTCGCATGGAAACAAAAAATTTCCTACGCGCACGAGGAGCCTATCATGGTGATGCCGCTCTACGAGAGGATGTGGCAATCTACCGTGCCCTTGTAGACCGTACGACGAAGCGTTAGTGAACGCGGTTGATGTAGTGGAACGTCCCTCACCGTCCCTCGATCCGCCCCGCGAACTATCCCGCGAACGAGTCCGATCTGAGTGCCGAACGGACGGCACCTCCAGCGTTCCGCACACGTACGGCTCGACGATGATCTCGGCCTTCTTGATCCGGCAAGAGAGACGGAGAGGTGAAGGTTCTCACGACGGCGTGACGGCGCTCGGAGGTTGGTGATGATCTTGTCTCATCGGGGGCTGCGCGACTCCGCGAAACGCGATCTAGAGGTGAAACCGTGGAGATATGTGGTCGGGCTGCCGTGGCAAAAGTTGTCTCAAATCGGCCCTAAACCCTCATATATAGGAGGAGGAGGAGACTTGCCTTGGGGGTCAAGGACTCCCAAGGGAGTCGGCAGGAGCCAGGGAAGGCCTCCCCCTCCCCAAGCCAATTCTACTTGGTTTGGAAAGGTGGAGTCCTTCTTCCCTTTCCCACCAGCCTCCCCCTCCCAAACCGAATTATACTTGGTTTGGAAGGTGGGTCCTTCTTCCCTTCCCACCTCCTTCTTTTTTCCTTTCCTCTTTGATTTTCTCACATGCGCATAGGCTCTTTCTTGGGCTCTCTCACCAGCCCACTAAGGGCTGGTGCGCCACCCCCAAGGCATATGGGCTTCCCCGGGGTGGGTTGCCCCCCTCCGGTGAACACCGGAACCCATTCGTCATTCCGGTACATTCCCGGTAACAGAAAACCTTCGGTAATCAAATGAGGTCATCCTATATATCAATCTTCGTTTCCGGACCATTCGGAAACCCTGCGTGACGTCCATGATCTCATCCAGGGACTCCGAACAACATTCGGTAACCAACCATATAACTCAAATACGCATAAAACAAGTCGAACCTTAAGTGTGCGAGACCCTACGGGTTCGAGAACTATGTAGACATGACCAGAGACTCCTCGGTCAATATCCAATAAGCGGGACTGGATGCCCATATTGGATCCTACATATTCTACAAAGATCTTATCGTTTGAACCTCGGTGGGATTCATATAATCGTATGTCATTCCCTTTGTCCTTCGGTATGTTACTTGCCCAGGATTCGATCGTCGGTATCCGCATACCTATTTAGGTCTCGTTTAGGCAAGTCTCTTTACTCGTTCGGTAATACAAGATCCCGCAACTTACACTAAGTCACATTGCTTGCAAGGCTTGTGTGATGTTGTGGCGAGTGGGCAGAGATACCTCTCCGTCACACGGAGTGACAAATCCAGTCTCGATCCATACTAACTCAACGAACACCTTCGGAGATACACCTTAGAGCATCTTTATAGTCACCGGTTACGTTGCGACGTTTGATACACACAAGCATTCCTCCGGTGTCCGTGAGTTATATGATCTCATGGTCATAGGAACAAATACTTGACACGCAAAAAATAGTAGCAACAAAATGACACGATCAACATGCTACGTCTATTAGTTTGGGTCTAAGTCCATCACATGATTCTCCTAATGATGTGATCAGTTAGCAAGGGACAACACTTGCCTATGGCCCGGAAACCTTGACCATCTTTGATCAACGAGCTGACTAGTCAACTAGAGGCTTACTAGGGACAGTGTTTTGTCTATGTATCCACACAAGTATTGTGTTTCAATCAATACAATTATAGCATGGATAATAAATGATTAT

>ONT2

AGCATTGCTTCGTTCGGTTACGTATTGCTAATTGGCAGGCGGTATATACTTCTAATAAGTCTAAAGCATAAAGGAAGAACAAGCCTGATGCGAATGTGTTACATAGCTCGAACCATGCATTCTGATGCCGAATCAAGCAGAAGCAACTCTAAGCTCTGTTTCCTCCATTTTGATCTAACCATACGGTATAACGGTAAGGTAACTTGCTATAGTACAAGATACAAGGTGTACAGATCCCATCCTTATCATTGCACAAGATCCGGCAACTCGCAAGCGCGCCTTGGCACCAAAACAAATCACTGGCGCGCCTTGGCACCAAAGAAATGATCTTGCAGTTTGTTAGTAGGATCTTCTTACCTTTTTGCTTGTTTGCATCCATTCGTCTCATATATGTTCCATGAAATCAATCCTCTTACATAACCAGCCCGAGACCGCCACCGCAGTCGTCGTCCTCATCAACAAGACCAATGGAGCCACTGGGAAGCAGCGGAACGACAAAGCACGATCTGTAGTAGGCTAGTCGCCAATGTGCTGTTACCAAAACCTCACCAACTCAGCATGGATGTACCTCCGGTAGCGTTCCGGGTGGGGATGAGCCTCTACAACCAGCAACTTCAGCGGCGGCTGGGCCGGTCCCGTCCATCAACAGGTTGGTTCGCGTGTTGGAGTACATGAGCTATCTGTAAGGCTACTGATTGTTGTAGCCTGCTTCATCCAAAAATTTATTAGAGCCCTTGAATGAATGTACGTGTGTTCGGAAAAGGAAAAATACCGTGTTGCTGGCTTGAGTTTTCATTGGTTTTCTCATCTGATGATGCACAGTCTTACTTATGGTAAAATCTTGTTCTGCTGCCGTGAGTGGTTTTCTAGTCGGATGCCATATTTACTTATCTCTTACTCTCAGTTTTCACCATCACTAATGATTTTATCCTCTTGGTGGCCTCATCAGTTATAACTCTCTTTTTCTCTAAGTCAAAATAAAAATCTACAATACTTTTGCCACAATTTTTTTCTATAATACACTTGAAAACAAAATTGAAATTTAACATTTTTCTGAATTAAGGTCCAGTCCTTTTTGTTTACCGAAAGTTTAATTCGATGATTGTAGTAACAAGGTAACTTGCTATACAAGATACAAGATATACAAATCCCTCTTATCATTCTACAAGATCCAGCAAATCACTGTATAAAACCTAGAGTCAATGACATTCTCCGGATTTATGAGGACTGCTCAGGTCCGGCTGTGAACCGAGAAAAAAGTTCATTCTTGTTCAGTGCAAATACAGGTCAGCCCTTAAAAGGGACATTAAAATCTGCTCTTGGGATTCCGGTGGAAGCGTTTTCAGAACGCTACCTGGGCCTACCTACTGCAATTGGAAGAATCACCAACGGCACATTCTCTCTTATTGAGGGTGCATGGCCAAAATGTGGTTGGTCGGTGAGGAACCTAGCCTGTGCGGGACGGAAGGTTTTGCTGAAATCTGTTGCCGAGGCTATACCGACATACATTATGAGTTGTTTCTTGTTGACAAAATAAGTTTGCAAAAAATCACCTCACCAATGGCCAAATATTGGTGGGGCAATTCCTATTTACAAGAGAGCACTTCATTGTCTATCGTGGAAGAAATTGACTGAGTCGAAGATCAAAGGAGGGATGGGTTTTAGGGACCCTGAACTATTTAATCTTGCCATGTTAGGCAAACAGGACTGGCAGTTGTTGACACACCCCAACAGTTTATGTGCGCGTGTACTGAAGGGAAAGTACTACCCATATACTGATTTTCTGCATGCAACGATCCCCAGTCGAGCATCATCTACCTGGAGAGCGTTCGTGCCGGGCAGAAAAGCTTTGGATAATGCACTTATTCACAGAATTGAAGACGGCTCCTCTGTTTCTATATGGACAGACCATTGGATCAAGCAGCAACGTTGAAACCCATGGGCCGGATTGGTAACGCACCTTTGGAAAGGGTTTCAGATTTGATTGATGCTTATACAGGAAGTTGGGATGTGGATCTGGACCGACAAAACTTTCTTCACCCAGATGCGGAGGCGATTTTGAATATACCTTTGAATCCGGCTGGGGATGATGATACTTTGGCATGGGCTCTGGAAAAGTTGGGTCATTATACTGTTAAATCGGCGTATCGTCCTCTCGCGACTCGCAATGGGCTTAGTACTCTAGAGGAAGGGATGATCACGGACACTTCATCTTCAAACAAACAGTTATGGAACTATGGAAACTACATGTTGTACCAAAGGTTTGAGTGTTCTGGTTGGAGGGTTCTTCATGGCATCCTACCAGACTAAATGACCTGAGTATCGTCATATTAAACCGATTGGTGGGTGTGACATATGTCAGGCCATGGATGATACTCTTATGCATGCGCTCATCTCTTGTACACATGCTAAGAAGTTTTGGATTGGCAAAGACTGGTTCGAGAGCTGAATCTACCTAGGCTCCGACCACACACTTGGGCGCGTGACATTTTGCTGGACCCAGCAATTCTCTGCAGATGCTATTAGATGCAAGATCATAATTATCATGCATGCCATTTGGTCTTCTCGTAGTATATGGACTCATGATAAAGATGGGTATGACCCAGTTTCTGGCCATCAAATGGGTGCATGAAACCCTAGCACTCCTCAAGCTTCCGACACGGCGAGTAAGTTCTACTTCAGCACAATCCTGGCATTCACCGGAGGTGGGATGGATCACTATCAATGCCGATGGATCGGTGTTTCCAGATGCTCCCTCAGCAGGAGCAGGAGGGAAGTAGCTCACTCGCACCTGGCATTCTTGGCAGCCTGGTGCAAGCCACTTACCGGGAGTTTCAAATCCTTTTGTCTCTGAGCTGCTGGCGTTATGGGAGGGAGTGATTTTTCTCAGCTGTGGAGATACTCACATGTGATAATACAAGTTGATTGCCCGAGTTGATCGACCTTTGGAATTGCACATCACCACAAAATCAATTGTGACGTCTATCTTAAGAGAATTAGGGGCTATTATACCTAGTTTTGTTTCCTTTTCCTCGGCATGTTAGTAAGGAACTTAATGTTTCAGCTCACGAGTGTGCCCAATGTGCGTGCTCACTAGATGGGACGGAAAGCTGGCTAGACCTGGCCCCGATTTCCTTATCAGTTGCCTACGGTCTGGTTGTAACAGGTTACTGCTTCGTTGAATAAAGCTTCGAATTCATTGAAAAAGACAAAAACATGTGTGTGCCTTGGCACGAAAATGATCTTGCACAGTTTGCTAGTAGGAGATCTTTTCTTAACTTTTCTTTTCTTTTGCATCCATTCATCCCAAACACGTTCCATGAAATCAATCCTCTCTACATAACCGGCCCGAGACACACACACCACCGCAGTTGTTGATGTTCACTGATTCCTGATTCCGTTGGATGCATGCATGTTGTGGTATTAGGTGCTTAGTTGAATTGAAAGGTTGTTAGAGCATACACAACGCTAACGTACAAATGGGTCATGACAGCCACATCGGACGCGTCCATGGACACTAGCCGCTCACGCCTTAAATAATATATTTTACAACCGAATATCTTAAATTAAAAACCTCAAATTCATATTATTACTTGTAATCGGCGGCATAACTATTTTTCCTAAGTCAAAAGAAAATTCTATATTTTACTAGAAACAAAAATAAAATTCAAAACAAACATTTTGCTGAATTAAGGTCAGTCTCTTTTTTTGCCAAAAATTTAATTCGATCTTAGTAATACAGTAACTTGCTATACAAGGTACAAAACGTATGAATCACATCTTATCATTCTACAAGATCCAACCAAATTATGGCCGTGCCTTGGCATCAATGATGGAAATTAAACCACCTTCAACATCGTCTCATGCTAGCATGTAAGATTCTGACAAGGGCATCCGTTGGAACTTTCATCCCGATGAGGTGTGTGTTCTCTGCTAAAAGTATAGGGTCCAACTGTGACCTAACACGGTACAACAGCACAAGATCATGGTGTTCAGTGGGCATCTTCTCATGCCCGCTTCTCTGATGCGTGACAAGCTCAAATAATTTGGATTTTGTTCAACACTTTCCTTCTAATTTGCTGGGTACAACAGCGAGCGAAGTCATGGTGGTCACTTGGTACTTCAACAACAAGACCATACCTTTTACGGAGCTCATCCGCGTGCTTGATAGGGGCGTTGAATTTTCGTGGGCTTGGGTTTTGGGCAAGTATTTTAAATAAGCGAGTACATAATGAATAACGGCGGAAATACAAATCAGCTATAGCGGAGCTAAAGCAGGCTATAGCGTTCTATTTATACGTGATACATTTAACGGCACCATGATAAAGGTATAGCAAGGCTATAGAGGGACTATAGCCGGCTATTTAAAACAATGGTTTTGGGTCATAGCTGCAACGGTTCTTCGTCGAGCACTTCGCGGAAGCTCCGAGGTGTGTTATACGTCGCTTTGGTTCTTCACCAAGCTTCCATCGGCTCCTTGCTCCTCCACCCATAGGAAATCTCCCGGTGCAGCTTATTCTTAGCCACAAAGTATTCTAGCATCGGGCATGTGATTATAGCCTGGCTAAGCTAAGCCCCCCCCCCCCCTACACACCCACACCCACACACACACACATACGCACGCACGCACACACACGAAACGTCACATCTAATCAGAAGAAACAATAATGTGCCCAGGGCCGGTCCATACATATTCGGGGCCTTGGGAAGGAGACGACAAAATGGGTCCTTGTATACAAATAAAATTAAGAATATATTATATTTCTAAAGTAATTCTGTACATAGCATAGATTGATGGTCGAGAGAACTACATCATAATAGTATGGAAATAATATAGTTCTACAAAAGAGCTTCTTTTAAGTTGCACAAAAAAGACTATCATCTTGCTCTTCTAAGTATGAAACCATAGTCAAAACTGATAGCAACTTGAACCATTTATGTTTTCACTTTATTTTCTCACAGATAAACATTTCTTAGAACACATGTCACCAAATAATTACAGAAAAACTGTTAATACAAGGAACAAAAGCATTTGGGTATGGGCATATGGCTTGATGTCGAATTGTCGATGTAGTTTTTCTGGATCCAATGAAGGTTGAAGAACAGGCCAACAAAAGAGTAGAAACTAGGAAAGAAAAATTGAAGATGTCGGGTGGAGCAATCGAGAAGCGAAGAGGCGGGTTTGTTTAACAGCGAATGAAAATAATACTTGATTAGGCTAAGGAGGGAGACGTACCATCTCGATGTGGTGATGCTTCAAACGTCTATTGATGGGGAACACCGAGGATGCTATAAGAAGTGATTGGGGAAGGGAATAGCACCATCGGTCTCCATTAGTGGATGCACGGGAGCACCATCTCGAATTTGAAAGTGAACGGGAGCGACTACGGCTGTTCTCGGGATTGAAGAATTTGGGGGAGGGCTTGAAAGAGGCAAGATTTAGGAGTCGAGGAGGCGAGATGGCCGTAGGAGATCCCGATGGAGTGCTGCGGCGGCATAGTAAACGGAACGATTCTTTCGATCCGGAAAGCTACCTACGTTGTGGGTTTGGGCCTTTTTTCTTCTTCCTTTTTTCTGGATCTAAATAAAAAATATACTAATACATATATGTAATACTACCTGGGATATATTGTGGTGTAAACAAATTGACGCTTAGACAACTTAATAACACATTGCGGACGTTTTTAATGTACTGGGGTTGAACACTCTGTGCCGAATTCAGGATCCAGCGGCCGTGATTTTCCTTTCTTTCTAACGGTTTTGAAATTGCAAGTATTGGAGTATATCATCATTCTACTATTATCTTCCAATGCTACGAAATAGAGAAATCAAGCAGCGAAGAAACAAAAAAGATGTTACATGAGATGACAAATTAAAGCCCAATTACCTCGAATCATATATCAAATTGAATGTCGCAAAGATTTCAACACACGCCTACAGCGTTCTGAAAAGAAAGCCCAAATGCTCATAATTGGCTCCAAATATAAATCAGGACAGCATAGTCCTTCCAGCATCACTTCCCTCCAGAATAATCTTTTATATCCCATAGGCCATTGCAAAGCTCATTAAGATAAAAGTAAATGACGATTTAATAAGAATATGCAGCATTTTCAAGCTTGCAACTCAGATAAAACATCAACAGGAATGAGATGCAAATGAGCAAATTGAGCACATATTTAGGTTCAAATCCAACTTATTCATAAAGCATACTGCTGTCCCACAGGGAAACTGTTTCATAAAACTAACATATCATACGGACTCGATGGTCCATTAAACCAGACACAAGTAGACATCAGCACCACAAATACTTGACGAACAGAGGCTCGGGACGACCAGGCACATCGGCAGCTCCACCAAACTGGCTGAAAGCCTATTTCTTTGCCCTCGGACGGAGTGCTGGGGCGTCGGTTTCCACTATCGGCGAGTACTTCTACACAGCCATCGGTCCAGACGACGCTTCTGCGGGCGATTTGTGTCGCCCGACAGTCCCGGCTCCGGATCGGACGATTGCGTCGCATCGACCCTGCGCCCAAGCTGCATCATCGAAATTGCCGTCAACCAAGCTCTGATAGAGTTGGTCAAGACCAATGCGGAGCATATACGCCCAGGTCGTGGCGATCCTGCAAGCTCCGGATGCCTCCGCTCGAAGTAGCGCGTCTGCTGCTCCACCTAAAACCAACACGGCCTCAGAAGAAGATGTTGGCGACCTCATGTTGGGAATCCCCGAACATCGCCTCGCTCCAGTCAATGACCGCTGTTATGCGGCCATTGTCCGTCAGGACATTGTTGGAGCCGAAATCCGCGTACGAGGTGCCGGACTTCGGGGCAGTCCTCGGCCCAAAGCATCAATGCATCGAGAGCCTGCGCGACGGACGCACTGACGGTGTCGTCCATCACAGTTTGCCAGTGATACACATGGGGATCAGCAATCGCGCATATGAAATCACGCCATGTAGTGTATTGACCGATTCCTTGCGGTCGAATGGGCCGAACCCGCTCGTCTGGCTAAGATCGGCCGCAGCGATCGCATCCATAGCCTCCGCGACTTCGGTTGTAGAACAGCGGGCAGTTCGGTTTCAGGCAGGTCTTGCAACGTGACACCCTGTGAACGGCGGGAGATGCAATAGGTCAGGCTCTCGCTAAACTCCCCAATGTCAAGCACTTCCGGAATCGGGAGCGCGGCCGATGCAAAGTGCCGATAAACATAACGATCTTTGTAGAAACCATCGGCGCAGCTATTTACCCGCAGGACATATCCACGCCCTCCTACATCGAAGCTGAAAGCACGAGATTCTTCGCCCTCCGAGAGCTGCATCAGGTCGGACACGCTGTCGAACTTTTCGATCAGAAACTTCTCGACAGACGTCGCGGTGAGTTCAGGCTTTTCATTCCACCACGGAGGCGGAGGACAAGGTGAAGGGTGGACTCCTTCTGGATGTTGTAGTCAGCAAGGTCCTGCCATCTTCGAGCTGCTTGCCAGCGAAGATCAATCGCTGCTGGTCGAGGATGCCCTCCTTATCTTGGATCTTAGCCTTGACATTGTCGATGGTGTCAGAGGACTCCACCTCCAGGGTGATAGTCTTGCCGGTCAATGTTTTGACAAAGATCTGCATCTGCAAGAAATAATCACCAAACAGATAGAACAAGAACTAGCACAAGATTAAGGACACAACAACGGATCAAACAGCAAACATATTCCAACAGAACATACATGTATTCAAAAGATAATGCCGACACATCACACATGTATTCAGATAGGTAGTAACAGCACACGTATCCTGAACTTCAGAAATAAGTAGAATGAACTAGTGGCAATAATCTTCACACAGATAGTAAAAAAGAAATTACCACAATAGATTATATAGATGAACTACAGATTGTAACATGATATTACTACAGATTGTAACATGATATTCTAATATAGTTCTTAGCATAAATTACTGGCAGATAGTAACATGAGATCGATATGACAGATATAGCGACAAGACTTCCTATTTACCATTGACAATTAATTAAAAAGAGTTCATCATTATCTAATCTGTGTGAACATATGAAATCAAATCGAAACGCACAAGCTACATAATTGAACCATGTCTAAGAAGAACTACCCTACAGATCTACATCAAACACAAAACAGATATAACATATTAAAGCCTGTGTTTGCACCAACCCAAAAGGCACCAATAACTCAATCAAGAGCGGAACAATCAGATCTGAACATGCTCGCAACAAGGCCTACAAGATGGAACACGCTACAAATCGCACCAATCACTCAGGCATGACTAGTTCCACAATTCCCCAACAGATCTACACCGATTTCCCCAATCGAATCATCCAACAATTCATCTAACCGTAAAAATTCCCCCCAATCATCAGAACCAACATCCAATCCCAAACCTAACCACGATTCACACCTATCGAACCAACCGTGCCCAAATCACAACCATCAGATCTACAACCCCTAAACATCTCACGATAATCACATCGACAACCTAACCACCAATCGGATCTAGCAACAAAACAACCTCACCCGTCGAAATCGAATCGAAACGAAGCGAGGAGAGGAGAAGCAGCGTACCTTGAGGACGCGAGAGGATTCGATTCGACGACGAGAGCCTCGCGAGATTGGGAGAAATTTTTCGGGGGTGGAGCTGATGCGAGGAGAGGAGATGAGGGAGCTGGTATTTATGGCGGTTGGGTGGTGGGAGGAGTCCCGTGCCGTGACATCACCGTCTGCTTGGAGAATCCGCCACGCTGAAACCACCGCGGTTTCCGGGAATACGAGGCGGGCGAGCGAGCGGTTGGGAAATTTCGAGAAGATGCCGTTTGTCTCCGTTTGGTACACATCACGTTGATTTTTTTTGAGTGAATTACACGCTTTGGACCACATTTTATTATCTATAAGTTTTACTTTGGACTACCTTTAAACATATCTTTTCACTTTGAACCAGATAAATTTGCTATTGTTGCGATTTGGATTTTTTTCTCGTGCAATCAACGACCTTAAACACATCAGCTCTAGTATACGGCCGATCTCCTCTATATATGGTTCATATGTTTGCTGAAAGGGGAAGTTAGACATGACGAAAAGTTGTTCATGGTAGTCCAAACCACAACCCGGCCCAAATTGAAAGATAGGTTTAAGGGTGATCCAAATTGAGACTTAGGTAATAAAAGGTGGATCAAAGTGCAATTTACTTTTTTTACTGTAATTTCTTCTGGCTGGTTTGTTGGTCGCCGTTAGGACCGGGTGACGCCGTCAACCCCGCGCCTCCGTATTTGCTGACGTGGGGTGGCGCGCTGGCTTCCCCTTGACCCGAATCTCCGCAAGAATTCAAGCTTAGCGCCCATGTGGTTCAAAGGAAAGCTAGATGATGAAACGGTCGACGTTAGGACCAGGGATGAAAGTAGGATGGGAAAATCCCGTACCGACCGTTATCGTATAACCGATTTTGTTAGTTTTATCCCGATCGATTTCGAACCCGGGTAAAAAACGAAAACGGAACGGAAACGGGATATACAAAACGGTAAACGGAAACGGAAACGGTAGAGCTAGTTTCCCGACCGTTTCACCGGGATCCCGTTTTTAATCGGGATGATCCCGTTTCGTTACCGTATTTTCTAATTCAGGATGACTGCAATATGGCCTCCACTAGAATTCGAGCTCAGCGTCGATCTGAATAACATAGATGGCACCGCGCGCGATAATTTATCCTAGTTTGCGCGCTATATTTTGTTTTCTATCGCGTATTAAATGTATAATTGCGGGACTCTAATCATAAGCCCCATCTCATAAATAACGTCATGCATTACATGTTAATTATTACATGCTTAACGTAATTCAACAGAAATTATATGATAATCATCGCAAGACCGGCAACGGATTCAATCTTAAGAAACTTTATTGCCAAATGTTTGAACGATCTGCTTGACAAGCTCCTCTAGAATCTTGGAGCGCCTTGCCCCTCTTTGATCGGTTGGTTTCGATCCTGCCAAGGACTTTGTCCCAGATCTAAGCGACTATGAATCACCAACATTCGAAATAGATCTTCTCTCGAAACTTATGACATGACAACTTCCCCGCGCCTCTCTACCAACGGTGGATTCAACCAAGACTTTGTACTCGAAGAACCACTTGTTTGCTTCGACTTTGACTTCGGCGAACAGGCTATCGAAAATTATATAACCACATCGTCGGGACTGTTGGAGCCTTGTGATGCCAAGCCGGCTTATCGCCCTTCCCACCTTCACACGGCCAAGCAAATGCCCATTCCACAGTTGCAAGTCGGCCACGAGTCTTTGAAAGCGGACGGGACTTTGGGCGGCATTACGGCAGCACTTCAAGCGCTTTTGCAGCAAGCTGCAGAATGCCTCGATCGGCTCAAGACACACAAGAAGTCGGCACCAAAGGCTTTGCGACTCGCAAGGACCGTGCTCGGCATGAGTCTAAGCACAAACCAACGGTGCGGTGCCCTTGGCAAGACAAGGAAGGACAACAATGTACGAGGGTCTTTAGCGAGGGTGGATAACATGGGGCCATCCTCAATGTTGTGTCTGATCTTGAAGTTGACCTTGATGCCATTCTTTTGCTTGTCGGCCATGATGTACACATTGTGGGAGTTATAGTTGTATTCCAGCTTGTGGCGAGAATGTTTCCATCCTCCTTAAAGTCAATGCCCTTCAGCTCGATTCTATTCACCGGTGCTCCACCTTCGAACTTGACTTCGGCGCGCGGGTCTTGTAGTTCCCGTCATCTTTGAAAAAGATGGTTCTCTCCTGCACATAGCCCTCCCATGTTATCCACCCTGCTAAAGACCCTCGGACATTGTTGTCCTTCCTTGTCTTGCCAAGGGCACTTGTTGGTTTGTGCTTAGACTCATGCGACACGGTCCTTGCGAGTCGCAAAACTGGTGCCGACTCTTGGGTCTTGAGCTGGCGGGGGCATTCGCGTAGCGGCAGAAAACTTGAAGTGTTGTCACCGCCTAAAGTCCCCGTCCGCTTTCAAAAACTGTGGCGCGACCTGCTTAGGGAATGGGCATTTGCTTGGCCGTGTGAGAGTGGGAAGGGCGATAAGGCACGGCTTGGCACTGCGTCCAACAGTCCCGACGATGTGGTTATATAATTTTCGATAGCGGGTTCAGCGAAGTCAAAGTCGAAGCAAACAAGTGGTTCTTCGGAGTACAAGGTCTTGGTTGAATCCACCGTTGGTAGAGAAAGTATGTGGGGAAGTTGTCAAGATCATAAGTTTACGAGAGAAGATCTATTTCGAATGTTGGTGATTCATAGTCGTTTAGATCTGGGACAAAGTCCTTGGCGAGGATCGGAAACGCAACTCGATCAAGGGGCAAGGCGCTCAAGATTCGTAGTGATGGAAAGGGGCCGACTACCGGGGATCCTCTAGAGTCGACACAGAGTAACACCAAACAACAGGGTGAGCATCGACAAAAGAAACGGTACCAAACAAATAAATAGCGTATGAAGGCAGGGCAAAAATCATAGCTGCTGCATATGCCATCATCCAAGTATATCAAGATCAAAATAATTTATAAAACATACTTGTTTATTATAATTAGGTACTCAGACGACATATGAATAAGAATCTTTACCATATGCCATCATGTATATGCATCGGTAAAACCCACATCAACATGTATACCTATCCTAGATCGATATTTCCATCCATCTTAAACTCGTAACTATGAAGATGTATGACACACACATACAGTTCAAAATTAATAAATACACCGGGTAGTTTGAAACTACCACTCCGATCTGAACGAATGAACGACCGCCCAACCACACCACATCATCACAACCAAGCGAACCAAAAGCATCTCTTCGTATATGCATCGATGAAGCCGCATCAACATGTATACCTATCCTAGATCGATATTTCCATCCATCATCTTCAATTCGTAACTATGAATATGTATGGCACACACATACAGATCCAAAATTAATAAATCCACGGAGTAGTTTGAAACAGAATTCTACTCCGATCTAGAGCGACCGCCCAACCAGACCACATCATCACAACCAAGACAAAAAAAAAAGCATGAAAAGATGACTAGACAAACAAGTGCACGGCATATATTGAAATAAAGGAAAAGGGCAAACCAAACCCACCAATGCAACGAAACAAAAAAATCATGAAATCGATCCCGTACGCGGAACGGCTAGAGCCATCCCAGGATTCCCCAAAGAGAAACAACGGCAAGTTAGCAATCGAACGTGTCGACGTACGAATTCGCATCGAGTGTCTGACATCAGCAACACGGATCTAACACAAACACGGATCTAACACAAACATGAACAGAAGTAGAACTGCGGGCCCTAACCATGGACCGGAACGCCGATCTAGAGAAGGTAGAGAGGGGGGGAGGACGACGGCGTACCTTGAAGCGGAGGTGCCGACGGGTGGTTGGGGGAAATCTGGTTGTGTGTGGGATTTGGGGGAAATCTGGTTGTGTGTGTGTGCCGCTCCGAACAACACGAGGTTAGAAAAGAGGGTGTGGAGGGGGTGTCTATTTATTACGGCGGGCGAGGAAGGGAAAGCGAAGGAGCGATTGGGGAAAGGAATCCCCCGTAGCTGCCGTGCCGTGAGAGAGGAGGAGGCCGCCTGCCGTGCCGGCTCGTCTGCCGCTCCGCCACGCATTTCTGGATGCCGACAGCGGAGCAAGTCCAACGGTGGAGCGGAACTCTCGAGAGGGGTCCGAGAGGCAGCGGCACGGAGAGATGCCGTGCCGTCTGCTTCGCTTGGCCCGGCAGCGACGCTGCTGGTTCGCTGGTTGGTGTCCGTTAGACTCGTCGACGGCGTTTAACAGGGCGGCATTATCTACTCGAAACAAGAAAAATGTTTCCTTGGTTTTTTAATTTCTTAAAGGGTATTTGTTTAATTTTTAGTCACTTTATTTTATTCTATTTTATATCTAAATTATTAAATAAAAAAACTAAAATAGAGTTTTAGTTTTCTTAATTTAGAGGCTAAAATAGAATAAAATAGATGTACTAAAAAAATTAGTCTATAAAAACCATTAACCCTAAACCCTAAATATTGGATGTACTAATAAAATGGATGAAGTATTATATAGGTGAAGCTATTTTGCAAAAAAAAAGGAAGAGAACACATGCACACTAAAGATAAAACTGTAGAGTCCTGTTGTCAAAATGCTCAATTGTCCTTTAGACCATGTCTAACTGTTCATTTATATGATTCTCTAAAACACTGATATTATTGTAGTACTATAGATTATATTATTCGTAGAGTAAAGTTTAAATATGTATAAAGATAGATAAACTGCTTCAAACAAGTGTGACAAAAAAAATATGTGGTAATTTTTTATAACTTAGGCAATGCAATATACATTATCTCTAGAGAGGGGGCACGGCCCGGGTCACATGCACTGCAGCTCCTTACGAATTCCCATGGAGGCGTGTTTACAATTTTTTATATTTGTTTTTAAGTTTTGAATATATGTTTTCATGTGTGATTTTACCGGCAAAAATACCGGTTCCCGTCAGTTTCGAAGCTTTAACCCGACCGGATCGTATCGGTTTCGATTGCCGTATTTATCCGTTCGTTTTCGTTACCGGTATATCCCGTTTCGTTTCCGTCATAGGATTAAATATGAAAATGAAAACGGTAGAGTATTTACCGGCAGATTGCCGACCGTTTTTTCATCCCTAGTTAGGACGGCAATTGATTTCGGCAGCGCGGGTGTCCGTGTTCGCGGGACTCCGGAGGGATGCACATGTGACCGAGGGACACGAAGTGATCCGTTTAAGCTATCAGTGTTTAAACTAGCGATGGTTTAAACGGATCACTTCGTGTCTGGTCACCTCTGCATCCTCTGGAGTCCGGGGCCCAGACGGAGATTGCAGCTGTAATCAAAATCACCGTCTAACTAGGGATGAAAACGGTATTAGATGCATCTACCGTTTTCATTTTTCATATTTTAACTAGCCGAGACAGCGCAGAGCGAAAGCGAGAACGTGCCCAAGCTTGAACGGGATTAAATGCGATAATCGAAAAACCGATGCGAATCCCGTGATCATCAAAATAGACGAACTGTTCGGTAAAATCCTGCATGAAAACATATATTCAAAGCAAAAACAAATATAAAAATTGTAAACACGCTCCATGGTAAAACAAGTACCAGTCATTACCCCTATACAGAGATTAATGAGCATTGCATGTCTAAGTTATAAAGAGCCACATGACACTTTATCTATCTTTATACGTATATTAAACTTTACTCTAGAATAATATAATCTATGGTACTACAATAATATCAGTGTTTTAGAGAATCATATAAATGAACAGTTAGGTATGGTCACAAATATTTGTTGTTATCTTTGTGTGCATGTGTTCTCCTTTTTTGCAAAATAATACCTATATAATACTTCATCCATTTTATTGTACATCCATTTAAGGGTTTAGGAGTTAATGTACATCTATTTTATTCTATTTTAGCCTCTAAAGTCAAAACTAAAACTCTATTTTAATTTTTTATTTAATAATTTAGATATAAATAGAATAAATAAAGTGACTAAAAACAAATACTACAGAAAAATTAAAAAACTAAGGAAAACATTTTTCTTGTTTAGTAGATAATGCCAGCCTGTTCTGCCGTCGGCTGGCTGAGACACCAACCAAACGGAGCCCGGCCAGCGCCCGCCCAAACGGCTGTGTGCATCTCGCTCGCTGCCTCTGGACCCCTCTCCCGCGGTCCCTCCACCGTTGGACTATCATGTCGGCGCGGGGTGCGTGGCGGGCGGCAGGCGTGAGCCGGCTGCGCGCGCCCCTCCTCTCTCTGCGGCACGTGCGTGCGGGGTCTCCCCACCGCTCCTTACTTTCCCGCCTCGCCAGCCGTGTGACGCAGACACCCACCTCCTGCCCTCTTTCCCCAACCTCGTGTTGTTCGGAGCGCACACACACACAGCCTTTCCCCCAAATCCCACACAACCAGATTTCCCCAAATCCACCCCGTCAGCACCTCCGCTTCAAGGTACGCCGCTCGTCCTCCCCCCCCCCCCTCTCTACCTTCTCTAGATCGGCGTTCCGGTCCATGGTTAGGGCCCGGTAGTTCCTACTTCTGTTCATGTTTGTGTTAGATCCGTTGTGTTCGATCCGTGCTGCTAGATTCGTACACGGATGCGGCCTGTACGTCAGACACGTTCTGATTGCTGGCTTACCCGATGTTTCTGGGGAATCTGGGATGGCTCCTTCGTTCCTTTGAGACGGGATGTTCATGATTTTTTTGTTTTTTCGTTGCATAGGGTTTGGTTTGCCTTTTCCTTTATTTCAATATATGCCGTGCACTTGTTTGTCGGGTCATCTTTTCATGCTTTTTTTTTGTCTTGGTTGTGATGATGTGGTCTGGTTGGGCGGTCGTTCTAGATCGGAGTAGAATTCTGTTTTCAAACTACACGGTGGATTTATTAATTTTGGATCTGTATGTGTGTGCCATACATATTCATGAATTCTGAGTCGATGATGGATGGAAATATCGATCTAGGATAGGTATACATGTTGATGCGGAGTTTTCTTGATGCATATCACAGAATGCTTTTGTTAACTTGGTTGTGATGTGGTGTGGTGATCGTTCATTCGTTCTAGATGGGAGTAAAATGCTGTTTCCATTACCTGGTGTATTTATTAATTTTGGAACTGTATGTGTGTGTCATACATCTTCATAGTTACGAGTTTGAATGGATGGAAATATCGATCTAGGATAGGTATACATGTTGATGTGGGTTTTACTGATGCATATACATGATGGCTATGCAGCATCTATTCATATACTCCTAACCTTGAGTACTATCTATTATAATAAACAAGTATGTTTTATAATTATTTTGATCTTGATATACTTGGATGATGGCATATGCAGCGACTATATATTAGTTTTAGCCACTGCCTTCATACGCTATTTATTTGCTGTGCTATTTCTTTGTCGATGCTCACTGTTGTTTGGTGTTACTTCTGCAAGTCCAGGCTCTCAGAGAGATCCCCAGGTAATCCAGTCCCTTCCATCTCTGATCTTGGTATGCCCTCTTTGGTGAGTTGCGTTCCCGATCTGCCAAGGACTTTGTCCCGGATCTAAACGACTATGAATCACCAACATTCGAAATAGATCTTCTCTCGAAACTTATGACTTTGACAACTTCCCCACATACTCTCTACCAACGGTGGATTCAACCAAGACTTTGTACTCAGAAGAACCACTTGTTTGCTTCGACTTTGACTTCGCGAACCCGGCTATCGAAAATTATATAACCACATCGTCGGGATGATGGACGCGGTGCCAAGCCGGCTTATCGCCCCTTCCCCACCTTCACACGGCCAAGCTTGTGCCCATTCCCTAGTTGCAAGTCGGCCACGGTCTTTGAAAGCGGACGGGACTTTGGCGGCATTACGACAGCACTTCAAGCGCTTTTCGTCGCTGCTCGAATGCCCTCGGTCGGCTCAAGACACCTCGAAGTCGGCACCAAAGGCTTTGCGACTCGCAAGGGCAGGTGCTCGGCATGAGTCTAAGCACAAACCAACGGTGCGGTGCCCTTGGCAAGACAAGGAAGGACAACAATGTACGAGGGTCTTTAAAGGGGTGGATAACATGGGGCCATCCTCAATGTTGTGTACGATCTTGAAGTTGACCTTGATGCCATTCTTTTGCTTGTCGGCCATGATGTACACATTGTGGGAGTTATAGTTGTATTCCAGCTTGTGGCGAGAATGTTTTCCATCCTCCTTAAAGTCAATGCCCTTCAGCTCGATTCTACTTCACCGGGGTGTCCACCTTCGAACTTGACTTCGGCGCGGGTCTTGTAGTTCCCGTCATCTTTGAAAAAGATGGTTCTCTCTGCCGCCGCCCTCCCATGTTATCCACCACCTTTAAAGACCCTCAGACATTGTTGTCCTTCCTTGTCTTGCCAAGGGCACCGCAACTGTTGGTTTGTGCTTAGACTCATGCCGAGCACGGTCCTTGCGAGTGGCTTTGGTGCCGACTTCTTGCGGGTCTTGGTGGCTTGAGGGGCATTCGAGTAGCGACGAAAAAGCGCTTGAAGTGTTGCCGGTAATGCCGCCTAAAGTCCCGTCGGCTTTCAAAGACTGTGGCCGACTTGCAACTAGGGAATGGGCATTTGCTTGGCCGTGTGAAGGTGGGAAGGGCGATAAGGCCCGAAAGCAGCCTTGCGTCCAACGGTCCGACGATGTGGTTATATAATTTTCGATAGCCGGGTTCGCGAAGTCAAAGTCGAAGCAAGCAGTGGTTCTTCGGGTCCCAAAGTCTTGGTTGAATCCACCGTTGGTAGAGAGTATGTGGGGAAGTTGTCAAAGTCATAAGTTTACGAGAGAAGATCTATTTCGAATGTTGGTGATTCATAATCCGTTTAGATCGGGACAAGTCGCCCAGCGAGATCGGGAAACGCAACTCGATCAAAGAGGGGCAAGGCGCTCCAAGATTCGTAGTGATGGAAGGGGCCCGACTGCAGGGATCCTCTGAGTCGACCTGCGAAGTAACACCAAACAACGGGGTGAGCATCGACAAAAGCGATTACCAAGCAAATAAATAGCGTATGAAGGCGAGGCTAAAAAAATCCACATATAGCTCTTGCATATGCCATCATCCAAGTATATCAAGATCAAAATAATTATAAAACATACTTGTTTATTATAATAGATAGGTACTCAAGGTTAGAGCATATGAATAGATGCTGCATATGCCATCATGTATATGCATCGGTAAAACCCACATCAACATGTATACCTATCCTAGATCGATATTTCCATCCATCTTAAACTCATGTATGAAGATGTATGACACACATGGGATTCCAGAATTAATAAATACACCAGGTAGTTTGAAACAGTATTCTACTCCGATCTAGAACGAATGAGCATTTTTAACCACACCACATCATCACAACCAAGCGAACAAAAAGCATCTCTGTATATGCATCGGTAAAACCCGCATCAACATGTATACCTATCCTAGATCGATATTTCCATCCATCATCTTCAATTCGTAACTATGAATATGTATGGCACACACATACAGATCCAAAAGTCAATAAATCCACCGGGTAGTTTGAAACGAAATTCTACTCCGATCTAGAACGACCGCCCAACCGGACCACATCATCACAACCAAGACAAAAAAAAGCATGGAAAAGATGACCCGACAAACAAGTGCACGGCATATATTGAAATAAAGGAAAAGGGCAAACCAAACCCTATGCAACGAAACAAAAAAATCAATGAAATCGATCCCGTACGAGCCGGCTAGAGCCATCCCGGGATTCCCAAAGAGAAACAACGGCAAGTTAGCAATCGAACGTGTACGACGTACGAATTCGCATCCAGGTGTACGAACGCTAGCAGCACGGATCTAACACAAACACGGATCTAACACAAACATGAACAGAAGTAGAACTACCGGGCCCTAACCATGGACCGGAACGCCGATCTAGAGAAGGTAGAGGGGGGGGAGGAGACGAACGGCGTACCTTGAGAGCGGAGGTGCCGACAGGTTGGATTTGGGGGAAATCTGGTTGTGTGTGGGATTTGGGGGAAATCTGGTTGTGTGTATGCTCCGAACAACACGAGGTTGGGGAAAGAGGGTGTGGAGGGGGTGTCTATTTATTACGAGCGGGCGAGGAAGGGAAAGCGAAGGAGCGGTGGGAAAGGAATCCCCGTAGCTGCCGTGCCGTGAGGAGGGAGGAGGCCGCCTGCCGTGCCCGCTCACGTCTGCCGCTCCGCCACGCAATTTCTGGATGCCGACAGCGGAGCAAGTCCAACGGTGGAGACGGAACTCTCGAGAGGGGTCCGAGGCAGCAGCAGAGAGGAATGCCGTGCCGTACGCCGCTTGGCCCGGCCGACGCTGCTGGTTCGCTGGTTGGTGTCCGTTAGACTCGTCGACGGCGTTTAACAGTGGCATTATCTACTCGAAACAAGAAAAATGTTTCCTTAGTTTTTTTAATTTCTTAAAGGGTATTTGTTTAATTTTTAGTCACTTTATTTTATTCTATTTTATATCTAAATTATTAAATAAAAAACTAAAATAGAGTTTTAGTTTTCTTAATTTAGAGGCTAAAATAGAATAAAATAGATAATACTTAAAAAAATTAGTCTATAAAAACCATTAACCCTAAACCCTAAATGGATGTACTAATAAAATGGATGAAGTATTATATAGGTGAAGCTATTTGCAAAAAAGAGAACACATGCACACTAAAGATAAAACTGTAGAGTCTGTTGTCAAAATACTCAATTGTCCTTTAGACCATGTCTAACTGTTCATTTATATGATTCTCTAAAACACTGATATTATTGTAGTACTATAGATTATATTATTCGTAGAGTAAAGTTTAAATAT

>ONT3

ACTTGTACTTCGTTCAGTTACGTATTGCTCTTTATACATATGTTCAACTTTACTCTACGAATAATATAATCTATAGTACTACAATAATATCAGTGTTTTAAGAAATCATATAAATGAACAGTTAGACATGGTCTAAAGGACAATTGAGTATTTTGACAACGGGACTCTACAGTTTTATCTTTTTTAGTGTGCATGTGTTCTCCTTTTTTTGCAAATAGCTTCACCTATATAATACTTCATCCATTTTATTAGTACATCCATTTAGGGTTTAGGGTTAATGGTTTTTATAGACTAATTTTTTTAGTACATCTATTTTGTCTATTTTAGCCTCTAAATTAAGAAAACTAAAACTCTATTTTAGTTTTTATTTAATAATTTAGATATAAAATAGAATAAAATAAAGTGACTAAAAATTAAACAAATACCCTTTAAGAAATTAAAAAAACTAAGGAAACATTTTTCTTGTTCAGTAGATAATGCCCATTTGTTAAACGCCGTCGCGAGTCTAACGGACTGCCAGCGAACCAGCAGCGCGTCGGGCCAAGCGACGACGGCACGGCATCTGTCCTTCTCTGGACCCTCTCGAGAGTTCCGCTCCACCGTTGGACTTGCTCCCGCTGTCGGCATCCAGAAATTGCGTGGCGGACGTGAGCCGGCACGGCAGGCGGCCTCCTCCTCCTCTCACGGCACGGCAGCTACGGGGATTCCTTTCCCACCGCTCATCGCTTTCCCTTCCTCGCCGCCGTAATAAATAGACACCCCCTCCACACCTCTTTCCCCAACCTCGTGTTGTTCGGAGCGCACACACACACAGCCAGATTTCCCCCAAATCCCACACACAACCAGATTTCCCCCCAAATCCACCCGTCGGCACCTCCGCTTCAAGGTACGCCGCTCGTCCTCCCCCCCCCCCCCCCCTCTCTACCTTCTCTAGATCGGCGGTTCCGGTCCATGGTTAGGGCCGGTAGTTCTACTTCTGTTCATGTTTGTGTTAGATCCGTGTTTGTGTTAGATCGTGCTGCCCTTCAAACGTTCGTACACGGATGCGACACCGTACGTCGAGACACGTTCGATTGCTAACTTGCCGGTGTTTCTCTTTGGGGAATACGGGATGGCTCTAGCCGTTCCGCCGAGACGGGATCGATTTCATGATTTTTTTTTTGTTTCGTTGCATAGGGTTTGGTTTTCTTTATTTCAATATATGCCGTGCACTTGTTTGTCGGGTCATCTTTTCATGCTTTTTTTTTTTTGTCTTGGTTGTGATGATGTGGTGCAGTTTGGGCGGTCGTTCTAGATCGGAGTAGAATTACGGTACAAACTACACGGTGGATTTATTAATTTTGGATCTGTATGTGTGTGCCATACATATTCATAGTTACGAGTGAAGATGGATGGAAATATCGATCTAGGATAGGTATACATGTTGATGCGGGTTTTTTGCGAGTGCATATACGGAGATGCTTTTTGTTCGCTTGGTTGTGATGATGTGGTGTGGTTGGGCGGTCGTTCATTCGTTCTAGATGGGAGTAGAGATACTGTTTCAAACTACCTGGTGTATTTATTAATTTTGGAACTGTATGTGTGTGTCATACATCTTCATAGTTACGAGTTTAAGATGGATGGAAATATCGATCTAGGATAGGTATACATGTTGATGTGGGTTTTATCTTGATGCATATACATGATGGCATATGCAGCATCTATTCATATGCTCTAACCTTGAGTACCTATCTATTATAATAAACAAGTATGTTTTATAATTATTTTGATCTTGATATACTTGGATGATGGCATATGCGGCAGCTATATGTGGTTTTTTTAACCACACCTTCATACGCTATTTATTTGCTTGGTATCTTTTGTTTCTTTTGTCGATGCTCACCATTTGTTGTTTGGTGTGTTACTTACGCAGGTCGACTCTAGAGGATCCCCGGGTAGTCGGTCCTTCCATCACTGCAGATCTTGGAGCGCCTTGCCCTCTTTGATCGAGTTGCGTTTCCCGATCCTGCCAGGACTTTGTCCCAGAGATCTAAGCGACTATGAATCACCAACATTCGAAATAGATCTTCTCTCGGAAACTTATGACTTTGACAACTTCCCCACATGCTCTCTACCAACGGTGGATTCAACCAAGACTTTGTACTCCAAGAACCACTTGTTTGCTTCGACTTTGACTTCGCGAACCCGGCTATCGAAAATTATATAACCACATCGTCGGAATTTGGACGCGGTGCCAAGCCGGCTTATCGCCCTTCCCACCTTCACACGGCCAAGCAAATGCCCATTCCCTAGTTGCAAGAGTCGGCTACAGTCTTTGAAGACGGACGGGACTTTAGGCGGCATTACCGGCAACACTTCAAGCGCTTTTCGTCGCTACTCGGAATGCCCTCGGTCGGCTCAAGACCCTTGCAAGAAGTCGGCACCAAGGCTTTGCGACTCGCAAGGACCGTGCTCGGCATGAGTCTAAGCACAAACCAACAGTGCGGTGCCCTTGGCAAGACAAGGAAGGACAACAATGTCTGAGGGTCTTTAGCGGGGTGGATAACATGGGAGGGCTATGTGCGGAGAGAACCATCTTTTTCAAAGATGACGGGAACTACAAGACCCGCGAGCGAAGTCAGAAGTTCGGGCAGCTGGTGAATAGAATCGAGCTGAAGGGCATTGACTTTAAGGAGGATGGAAACATTCTCGGCCACAAGCTGGAATACAACCTTAACTCCCACAATGTGTACATCATGGCCGACAAGCAAAAGAATGGCATCAAGGTCAACTTCAAGATCGGACACAACATTGAGGATGGCCCCATGTTATCCACCCTGTTGCTAAAGACCCTCGGACATTGTTGTCCTTCCTTGTCTTGCCAAGGGCACCGCCCCATTGGTTTGTGCTTAGACTCATGCCGAGCACGGTCCTTGCGAGTCGCAAAGCCTTTGGTGCGGAGCTTCTTGCGAGGTCTTGAGGTAGTGAGGGCATTCGAGTAGCGACGGAAAAGCGCTTGAAGTGTTGCGGTAATGCCGCCTAAAGTCCGTCCGCTTTCAAAGGCTGGCCGACTTGCAGGGTTAGGGAATGGGCATTTGCTTGGCCGTGTGAAGGTGGGAAGGGCGATAAGGCGGCAACCTTTGCGTCCAACGATCCCGACGATGTGGTTATATAATTTTCGATAGCCGGGTTCGCGAAGTCAAAGTCGAAGCAAACAAGTGGTTCTTCGGAGTACAAAGTCTTGGTTGAATCCACCGTTGGTAGAGTATGTGGGAAGTTGTCAAAGTCATAAGTTTACGAGAGAAGATCTATTTCGAATGTTGGTGATTCATAGTCGTTTAGATGCAGGACAAAGTCCTTGGCGGGGATCGGGAAACGCAGCTCGATCAAAGAGGGAGCAAGGCGCTCCAAGATTCGTAGTGATGGAAGGGACGGCTACCGGGGATCCTCTAGAGTCGACCTGCAGAAGTAACACCAAACAACGGGTGAGCATCGACAAAAGAAACAGTACCAAGCAAATAAATAGCGTATGAAGGCGGGGCTAAAAAAATCCACATATAGGCTTGCTGCATAGTGCCATCATCCAAGTATATCAAGATCAAAATAATTATAAAACATACTTGTTTATTATAATAGATAGGTACTCAAGGTTAGAGCATATGAATAGATGCTGCATATGCCATCATGTATATGCATCAGTAAAACCCACATCAACATGTATACCTATCCTAGATCGATATTTCCATCCATCTTAAACTCGTAACTATGAAGATGTATGACACACATACAGTTCCAAAATTAATAAATACACCAGGTAGTTTGAAACAGTATTCTACTCCGATCTAGAACGAATGGTATGGCGCCCAACCACACCACATCATCACAACCAAGCGAACAAAAAGCATCTGTATATGCATCGGTAAAACCCGCATCAACATGTATACCTATCCTAGATCGATATTTCCATCCATCATCTTCAATTCGTAACTATGAATATGTATGGCACACATGCAGATCCCAAATTAATAAATCCGAGGGTAGTTTGAAACAGAATTCTACTCCGATCTAGAACGGCCAGCCCAACCGGACCACATCATCACAACCAAGACAAAAAAAAGCATGAAAAGATGACCCGACAAACAAGTGCACGGCATATATTGAAATAAAGGAAAAGGGCAAACCAAACCCTATGCAACGAAACAAAAAAAAATCATGAAATCGATCAGAGTACGGAACGGCTAGAGCCATCCCAGGATTCCCCAAAGAGAAACAACGGCAAGTTAGCAATCAGAACGTGTACGACGTACAGGTCGCATCCGTGTACGAACGCTAGCAGCACGGATCTAACACAAACACGGATCTAACACAAACATGAACAAGTAGAACTACCGGGCCCTAACCATGGACCGGAACGCCGATCTAGAGAAGGTAGAGAGGGGGGGGGGGAGGACGAGCGGCGTACCTTGAAGGCGGAGGTGCCGACGGGTGGATTTGGGGAAATCTGGTTGTGTGTGGGATTTGGGGGAAATCTGGTTGTGTGTGTGTGCGCTCCGAACAACACGAGGTTGGGGAAAGAGGGGTGTGGAGGGGGGGTGTCTATTTATTACGGCGGGCGGAGAAAGCGAAGGAGCGGTGGGAAAGGAATCCCCACGTAGCTGCCGTGCCGTGAGAGGAGGAGGAGGCCGCCTGCGGGTGCCGGCTCACGTCTGCCGCTCCGCCACAATTTCTGGATGCGCCAGCGGAGCAAGTCCAACGGTGGAGCGGAACTCTCGAGAGGGGTCCAGAGGCAGCGACGAGATGCCGTGCCGTACGCCGCTTGGCCCGGCCGACGCTGCTGGTTCGCTGGTTGGTGTCCGTTGGAGCTCGTCGACGGCGTTTAACAGGCTGGCATTATCTACTCGAAACAAGAAAAATGTTTCCTTAGTTTTTTTAATTTCTTAAAGGGTATTTGTTTAATTTTTAGTCACTTTATTTTATTCTATTTTATATCTAAATTATTAAATAAAAAAACTAAAATAGAGTTTTAGTTTTCTTAATTTAGAGGCTAAAATAGAATAAAATAGATGTACTAAAGAGTTGGTCTGTAAGGAGCCATTGACACAAACCCTAAATGGATGTACTAATAAAATGGATGAAGTATTATATAGGTGAAGCTATTTGCAAAAAAAGGAGAACACATGCACACTAAAAGATAAAACTGTAGAGTCCTGTATTTGTCCAAATACTAATTGTCCTTTAGACCATGTCTAACTGTTCATTTATATGATTCTCTAAAACACTGATATTATTGTAGTACTATAGATTATATTATTCGTAGAGTAAAGTTTAAATATATGTATAAAGATAGATAAACTGCACTTCAAACAAGTGTGACAAAAAAAATATGTGGTAATTTTTTATAACTTAGACATGCAATGCTCATTATCTCTAGAGAGGGGCACGACCGGGTCACGCTGCACTGCAGCTCCTTACGAATTCCATGGAGCGTGTTTACAATTTTTTATATTTGTTTTTAAGTTTTGAATATATGTTTTCATGTGTGATTTTACCGAACAAAAATACCGGTTCTAGTCCGATTTCGACTTTAACCCGACGGATCGTATCGGTTTTCGATTACCGTATTTATCCCGTTGTTTCGTTACCGGTATATCCCGTTTTCGTTTCCGTCCGCAAGTTAAATATGAAAATGAAAACGGTAAGTATTTTACCGACCGTTACCGTCGTTTTCATCCTAGTTAAGGACGACGAGTGATTTCGGCCGCGGCCGTGCTCCGTGTTCGCCGGGACTCCCCGGAGAGGATGCTGTGGCCAGGGACACGAAGTGATCCGTTTAAACTATCGGTGTTTAAACACTGATAGTTTAAACGGATCGCCACGTCCTCGGTCATGTCGCCCATCTCGTGATCCTGCCGAAGCCGAAATTACGAGCACGGCCCCGGCCCGGCCGAGGTACTCGTCGTCTAATAAAAGGATGAAAACGATACGGCTCGATCGTTTCATTTTTCATGTGCGGGGCAAGCGAGATACTAAAGCAGCAGGACATTGTCAAACCGATACGATCCGTCATGTTGACAAAACACATGAAAACATATATTCAAAACTTAAAACAAATATAAAAAAATTGTAAACACGCCATGGGGAATTCGTGAGGTTGCAGTGCCGCGATGGTGATCCTCTCTAGGAATAATGACATTGCATGTCTAAGTTATAAAAAATTACCACATATTTGTCACACTTGTTTGAAGTGCAGTTTATCTATCTTTATACATATATTAAACTTTACTCTACGAATAATATAATCTATAATTACTACAATAATATCAGTGTTTTAGAGAATCATATAAATGAACAGTTAGACATGGTCTAAAGGACAATTGAGTATTTTGACAACAGGACTCTACAGTTTTATCTTTTTAGTGTGCATGTGTTCTCCTTTTTTTGCAAATAGCTTCACCTATATAATACTTCATCCATTTTATTAGTACATCCATTTAGGGTTTAGGGTTAATGGTTTTTATAGACTAATTTTTTTAGTACATCTATTTTATTCTATTTTAGCCTCTAAATTAAGAAAACTAAAACTCTATTTTAGTTTTTTTATTTAATAATTTAGATATAAATGAATAAAATAAAGTGACTAAAAATTAAACAAATACCCTTTAAGAAATTAAAAAAACTAAGGAAACATTTTTCTTGTTTCGAGTAGATAATGCCGACCTGTTAAACGCCGTCGGCAGGTGCGGACACCAACCAGCGAACCAGCAGCGTCGCGTCGGGCCAAGCGAAGCAGACGGCACGGCATCTCGTCGCTGCCTGCGGACCCCTCTCGAGAGTTCCCGCTCCACCGTTGGACTTGCTCCGCTGTCGGCATCCAGAAATTGCGTGGCGGAGCGGCAGACGTGAGCCGGCACGGCAGGCGGCCTCCTCCTCCTGCGGCACGGCAGCTACGGGGGATTCGCGCTTTCCCGCTCCTTCGCGCCTCCCTTCTCGCCCGCCGTAATAAATAGACACCCCCTCCACACCCTCTTTCCCCAACCTCGTGTTGTTCGGAGCGCACACACACACAACCAGATTTCCCCCAAATCCCACACACAACCAGATTTCCCCCAAATCCACCCGTCAGCACCTCCGCTTCAGGTACGCTCGTCCTCCCCCCCCCCTCTCTACCTTCTCTAGATCGGCGTTCCGGTCCATGGTTAGGGAGCCGGTAGTTCTACTTCTGTTCATGTTTGTGTTAGATCCGTGTTTGTGTTAGATCCGTGCTGCTAGCGTTCGTACACGGATGCGACACCGTACGTCAGACACGTTCGATTGCTAACTTGCCGAGTGTTTCTCTTTGGGGAATCCTGGGATGGCTCTAGCCATGAGACGGGATCGATTTCATGATTTTTTTTGTTTCGTTGCATAGGGTTTGGTTTGCCCTTTTCCTTTATTTCAATATATGCCGTTGCACTTGTTTGTCGGGTCATCTTTTCATGCTTTTGTCTTTTAGTTGTGATGATGTGGTCTGGTTGGGCGGTCGTTCTAGATCGGAGTAGAATTCTGTTTCAAATGCTGGTGGATTTATTAATTTGGATCTGTATGTGTGTGCCATACATATTCATAGTTACGAATTGAAGATGATGGATGGAAATATCGATCTAGGATAGGTATACATGTTGATGCGGGTTTTGCGATGCATATACGAGAGAGATGCTTTTTGTTCGCTTGGTTGTGATGATGTGGTGTGGTTTAGTGGTCGTTCATTCGTTCTAGATCGGAGTAGAATCTTGTTTCAAACTACACAGTGTATTTATTAATTTTGGAACTGTGTGTGTGTGTCATACATCTTCATAGTTACGAGTTTAAGATGGATGGAAATATCGATCTAGGATAGGTATGCATGTTGATGTGGGTTTTACGTGATGCATATACATGATGGCATATGCGGCATCTATTCATATGCTCTAACCTTGAGTACCTATCTATTATAATAAACAAGTATGTTTTATAATTATTTTGATCTTGATATACTTGGATGATGGCATATGCAGCAGCTATATGTGGATTTTTAGCCCTGCCTTCATACGCTATTTATTTGCTTGTGATACTTGTTTCTTTTGTCGATGCTCACCCTGTTGTTTGGTGTTACTTGCGGAGTCGACTCTAGAGGATCCGGGTAGTCGGTCCTTCCATCACTACGAATCTTGGACGCCTTGCCCCTCTTTGATCGAGACTAGTTTCCGATCTGCCAAGGACTTTGTCCCAGATCTAAACGACTATGAATCACCAACATTCGAAATAGATCTTCTCTCAGGAAAACTTATGACTTTGACAACTTCCCCACATACTCTCTACCAACGGTGGATTCAACCAAGACTTTGTACTCCAGAAGAACCACTTGTTTGTGACTTTGTCCGCGAACCCGGCTATCGAAAATTATATAACACATCGTCGGGACTGTTGGACGCAGTGCCAAGCCGGCTTATGGCCTTCCCACCTTCACACGGCCAAGCAAATGCCCATTCCCTAGTTGCAAGTCGGCCACAGTCTTTGAAAGCGGACGGGACTGCGGCGGCGTCTGAGCACTGCTAGCGCTTTTCGCCGCTACTCGGAATGCCCCTCGGTCAGCTCAAGACCTGCAAGAAGTCGGCACCAAAGGCTTTGCGACTCGCAAGGACCGTGCTCGGCATGAGTCTAAGCACAAACCAACGGTGCGGTGCCCTTAGCCAAGACAAGGAGACAACAATGTACGAGGTCTTTAGCGAGGTGGATAACATGGGAGGGCTATGTGCAGGAGAGAACCATCTTTTTCAAAGATGACGGGAACTACAAGACCCGCTAACGAGGTCAGAGTTCGAGTGGCTACAGTGAATAGAATCGAGCTGAAGGGCATTGACTTTGAGAGGATGGGGAAACATTCTCGGCCACAAGCCTAGATAGCTATAACTCCACAATGTGTACATCATGGCGGAGCAAGCAAAAAGAATGGCATCAAGGTCAACTAAATTAGACACAACATTGAGGATGGCCCCAAGTGTTATCCACCACCTTCTAAAGACCCTCGGACATTGTTGTCCTTCCTTGTCTTGCCAAGGGCACGCGCGTTGGTTTGTGCTTAGACTCATGCGGGAACCACGTGGATCCCTTGCGAGTGTAAAGCCTTTGGTGCCGACTTCTTGCGGGTCTCGGCTGGCGGGGCATTACGAGTAGCGACAGGAAAAACGCTTGAAGTGTTGCGGTAATGCCGCCTAAAGTCCGTCATAGCTTTTAAAGGCGCGCAGGCGGGCCTTGCAACTAGGGAATGGGCATTTGCTTGGCGGTGTGAAGGTGGGAAGGGCGGCGATAAAGTGGCTTGTACTTTGCGTCCAACAGTCCCGACGATGTGGTTATATAATTTTCGATAAGCGAGATTCGCGAAGTGCAAAGTCGAAGCAAACAAGTGGTTCGGAGTACAAAGTCTTGGTTGAATCCCATAGTTGGTAGAGTATGTGAAGTTGTCAAAGTCATAAGTTTGCGAGAAGATCTGTCGAATGTTGGTGATTCATAGTCGTTGATCTGGGACAAAGTCCTTGGCAGGATCGGGAAACGCAACTCGATCAAGAGAGCAAGGCGCTCCAAGATTCGTAGTGAGCTTGTCAAGCAGATCGTTCAAACATTTGGCAATAAAGTTTCTTAAGATTGAATCCTGTTGCCGGTCTTGCGATGATTATCATATAATTTCTGTTGAATTACGTTAAGCATGTAATAATTAACATGTAATGCATGACGTTATTTATGAGATGGGTTTTTATGATTAGAGTCCCGCAATTATACATTTAATACGATAGAAAACAAAATATAGCGCGCAAACTAGGATAAATTATCGCGCGCGGTGTCATCTATGTTACTAGATCGACGCTGAGCTCGAATTCTAATTAGAGCCATATTGCAGTCATCCCGAATTAGAAAATACGGTAACGAAACGGGATCATCCCGATTAAAAACGGGATCCCGGTGAAACAGTCGGAAGTAGCTCTACCGTTTCCGTTTCCGTTTACCGTTTTGTATATCCCGTTTCCGTTCCGTTTTCGTTTTTTACCTCGGGTTCGAAATCGATCGGGATAAAACTAACAAAATCGGTTATACGATAACGGTCGGTACGGGATTTTCCCATCCTACTTTCATCCCTGGTCCTAACGTCGACCGTTTCATCATCTAGCTTCTCCTTGAACCACATGGGCGCTAAGCTTGAATTCTTGCGGAGATTCGGGTCAAGGGGAAGCCAGCGCGCACCACCCCACGTCAGCAAATACGGAGGCGCGGGGTTGACGGCGTCACCCGGTCCTAACGGCGACCAACAAACCAGCCAGAAGAAATTACAGTAAAAAAGTAAATTGCACTTTGATCCACCTTTTATTACCTAAAGTCTCAGATTTGGATCACCCTTAAACCTATCTTTTCAATTTGGGCCGGGTTGTGGTTTGGACTACCATGAACAACTTTTCGTCATGTCTAACTTCCCTTTCAGCGACTTATATGAACCATATATAGAGGAGATCGGCCGTATACTAGAGCTGATGTGTTTAAGGTCGTTGATTGCACGAGAAAAAAAAATCCAAATCGCAACAATAGCAAATTTATCTGGTTCAAAGTGAAAAGATACTGAGTGAGTCCAAAGTAAAACTTATAGATAATAAAATGTGGTCCAAAGCGTAATTCACTCAAAAAAAAATCAACGTGATGTGTACCAAACGGAGACAAACGGCATCTTCTCGAAATTTCCCAACGCTCGCTCGCCCGCCTCGTATTCCCGGAAACCGCGGTGGTTTCAGCGTGGCGGATTCTCCAAGCAGACGGTGATGTCACGACACGGGACTCCTCCCACCACCCAACCGCCATAAATACCAGCCCCCTCATCTCCTCTCCTCGCATCAGCTCCACCCCCGAAAAATTTCTCCCCAATCTCGCGAGGCTCTCGTCGTCGAATCGAATCCTCTCGCGTCCTCAAGGTACGCTGCTTCTCCTCTCCTCGCTTCGTTTCGATTCGATTTCGGACGGGTGAGGTTGTTTGTTGCTAGATCCGATTGGTGGTTAGGGTTGTCGATGTGATTATCGTGAGATGTTTAGGGGTTGTAGATCTGATGGTTGTGATTTGGGCACGGTTGGTTCGATAGGTGGAATCGTGGTTAGGTTTTGGGATTGGATGTTGGTTCTGATGATTGGGGGGAATTTTTACGGTTAGATGAATTGTTGGATGATTCGATTGGGGGAAATCGGTGTAGATCTGTTGGGGAATTGTGGAACTAGTCATGCCTGAGTGATTGGTGCGATTTGTAGCGTGTTCCATCTTGTGGGCCTTGTTGCGAGCATGTTCAGATCCTACTGTTCCGCTCTTGATTGAGTTATTGGTGCCTTGGGTTGGTGCAAACACAGGCTTTAATATGTTATATCTGTTTGTGTTTGATGTAGATCTGTAGGGTAGTTCTTCTTAGACATGGTTCAATTATGTAGCTTGTGCGTTTCGATTTGATTTCATATGTTCACAGATTAGATAATGATGAACTCTTTTAATTAATTGTCAATGGTAAATAGGAAGTCTTGTCGCTATATCTGTCATAATGATCTCATGTTACTATCTGCCAGTAATTTATGCTAAGAACTATATTAGAATATCATATTGCAATCTGTAGTAATATCATGTTACAATCTGTAGTTCATCTATATAATCTATTGTGGTAATTTCTTTTTACTATCTGTGTGAAGATTATTGCCACTAGTTCATTCTACTTATTTCTGAAGTTCAGGATACGTGTGCTGTTACTACCTATCTGAATACATGTGTGATGTGCCTGTTACTATCTTTTGAATACATGTATGTTCTGTTGGAATATGTTTGCTGTTTGATCCGTTGTTGTGTCCTTAATCTTGTGCTAGTTCTTACCCTATCTGTTTGGTGATTATTTCTTGCAGATGCAGATCTTTGTCAAAACATTGACCGGCAAGACTATCACCCTGGGTGGAGTCCTCCTGACACCATCGACAATGTCAAGGCTAAGATCAGATAAGGAGGGCATCCCCCGGACCAGCAGCGATTGATCTTCGCTGGCAAGCAGCTCGAAGATGGCAGGACCCTTGCTGACTACAACATCCAGAAGGAGTCCACCCTTCACCTTGATCCTCCGCCTCCGTGGTGGAATGAAAAAGCCTGAACTCACCGCGACGTCTGTCGAGAAGTTTCTGATCGAAAAGTTCGACAGCGTGTCCGACCTGATGCAGCTCTCGGAGGGCGAAGAATCTCGTGCTTTCAGCTTCGATGTAGGAGGGCGTGTCCTGCGGGTAAATAGCTGCGCCGATGGTTTCTACAAAGATCGTTATGTTTATCGGCACTTTGCATCGGCCGCGCTCCCGATTCCGGAAGTGCTGACATTGGGGGAGTTTAGCGAGAGCCTGACCTATTGCATCTCCCGCCGTTCACAGGGTGTCACGTTGCAAGACCTGCCTGAAACCGAACTGCCCGCTGTTCTACAACCGGTCGCGGAGGCTATGGATGCGATCGCTGCGGCCGATCTTAGCCAGACGAGCGGGTTCGGCCATTCGGACCGCAAGGAATCGGTCAATACACTACATGGCGTGATTTCATATGCGCGATTGCTGATCCCCATGTGTATCACTGGAAACTGCGATGGACGACACCGTCAGTGCGTCCGTCGCGCAAGCTCCTCGAGCTGATGCTTTGGGCCGAGGACTGCCCCGAAGTCCGGCACCTCGTGCACGCGGATTTCGGCTCCAACAATGTCCTGACGGACAATGGCCGCATAACAGCGGTCATTGACTGGAGCGAGGCGATGTTCGGGGATTCCCAATGACGAGGTCGCCAACATCTTCTTCTGGAGGCCGTGGTTGGCTTGTATGGAGCAGCAGACGCGCTACGCTTCGAGCGGAGGGCATCGGAGCTTGCAGGATCGCCACGACTCCGGGCGTATATGCTCCGCATTGGTCTTGACCAACTCTATCAGAGCTTGGTTGACGGCAATTTCGATGATGCGGCAGGTACCCAGGGTCGATGCGACGCAATCGTCGATCGAACCGGGACTGTCGGGCGTACACAAATCGCCCGCAGAGCGCGGCCGTCTGGACCGATGGCTGTGTAGAAGTACTCATGGTAAAACCCCAGCACTCGTCCGAGGGCAAAGAAATAGGCTTTCAGCCAGTTTGGTGGAGCTGCCGATGTGCCTGGTCGTCCCGAGCCTCTGTTCGTCAAGTATTTGTGGTGCTGATGTCTACTTGTGTGGTTTAATGTCGAGTCCGTATGATATGTTAGTTTTATGAAACAGTTTCCTGTGGGACAGCAGTATGCTTTATGAATAAGTTGGATTTGAACCTAAATATGTGCTCAATTTGCTCATTTGCATCTCATTCCTGTTGATGTTTTATCAGGTTGCAAGTTTGAAAATGCTGCATATTCTTATTAAATCGTCATTTACTTTTATCTTAATGAGCTTTTGCAATGGCCTATGGGATATAAGGTATTCTGGAGGGGAAGTGATGCTGGAAGGACTATGCTGTGCATGATTTATATTTGGAGCCACTATGAGCATTTGGGCTTTCTTTTCAGAACGCTGTAGGCGTGTGTTGAAATCTTTGCGACATTCAATTTGATATATGATTCGAGGTAATTGGGCTTTAATTTGTCATCTCATGTAACATCTTTTTGTTTCTTCGCTGCTTGATTTCTCTATTTCGTAGCATTGGAAGATAATAGTAGAATGATGATATACTCAATACTTGCAATTTCAAAACCGTTAGAAAGAAAGGAAAATCACGGCCGCTGGATCCGAATTCGGCACAGAGTGTTCAACCCCAGTACATTAAAAACGTCCGCAATGTGTTATTAAGTTGTCTAATCAATTTGTTTACACAATATCCCAGGTAGTATTAATACATATATGTATTAGTATATTTTTATTTAGATCCAGAAAAAAAGGAAGAAGAAAAAAGGCCCAAACCCACAACGTAGGTAGCTTTCCCGGATCGAAAGAATCGTTCCCTAGTTTACTATCGCTGTTGCAACACTCCATCGATCTCCTACGGTCATCTCGCCTCCTCGACTCCTAAAAAATCTTGCCTCTTGACTCCTCCCCAAATTCTTCAATCACGGGAGAACGGCGTAGTCGCTCCCGTTCACTTTCAAATTCGAGATGGTGCTCCCGTGCATCCACTAATGGAGACCGATGGTGCTATTCCCTTCCCCAATCACTTCTTATAGCATCCTCGGTGTTCCCCATCAATAGACGTTTGAAGCATCACCACATCGGAGATGGTACGTCTCCCTCCTTAGCCTAATCAAGTATTATTTTCATTCGCTGTTAAACAACCCGCCTCTTCGCTTCGATTGCTCCACGACATCTTCAATTTTTCTCTTCCTAGTTTCTCTTTTGTTGGCCTGTTCTTCGAACCTTCATTGGATCCCGGGAAAACTACATCGGCAATTCGACATCAAGCCATATGCCCATACCCAAATGCTTTTGTTCCTTGTATTAACAGTTTTTTCTTGTGATTATTTGGTGACATGTGTTCTAAGAAATGTTTATCTGTGAGAAAATAAAGTGAAAAAAACATAAATGGTTCAAGTTGCTATCAGTTTTGACTATGGTTTCATACTTAGAAGAGCAAGATGATAGTCTTTTTGTGCAACTTAAAAGAAGCTCTTTTGTAGAACTATGTCATACTATTATGATGTAGTTCTCTCGACCATCAATCTATGCTATGTACAGAATTACTTTAGAAATATAATATATTCTTAATTTTTATTTGTATACAAGGACCCATTTGTCGTCTCTTCCCAAGGCCCCGAATATGTATGGACCGGCCCTGGGCACATTATTGTTTCTTCTGATTAGATGTGACGTTTCGTGTGTGTGCGTGCGTGCGTATGTGTGTGTGTGTGGGTGTGGGTGTGTAGGGGGGGGGGGGGGGGGGGGCTTAGCTAGGCCAGGCTATAATCACATGCCCGATGCTAGAATACTTTGTGGCTAAGAATAAATGCAGGAGATTTCCTACGGGTGGAGGAGCAAGGAGCCGATGGAAGCTTGGTGAAGAACCAAAGCGACGTATAACACACCTCAGAGCTACGGCGAAGTGCTCGACGAAGAACCGCGAGAGCTATGACCCAAAACCATTGTTTTAAATAGCCGGCTATAGTCCCTCTATAGCCTTGCTATACCTTTTTATCATGGTGCCGTTAAATGGTATCACGTATAAATAGAACGCTATAGCCCGCTTTAGCTCCGCTATAGCTGATTTAGAGCGCCAGATTATTTATTAAATGGCCGTGACAAACATTGCCCAAAACCCAAGCCCACGAAAAAATTCAACGCCCCTATCAAGCACGCGGATGAGCTCCGTAAAAGGTATGGTCTTGTTGTTGAAGTACCAAGTGACCACCATGATTCTGCTTGCTGTTGTACCCAGCAAATTAGAAGGGAAAGTGTTGAACAAAATCCAACATTTGAGCTTGTCACGCATCAGAGAAGCGGGCATGAGAAGATGCCCACTGAACACCATGATCTTGTGCTGTTGTACCGTGTTTAACGAGTTGGACCCCTATACTTTTAGCAGAGAACACACCTCATCGGGATGAAAGTTCCAACGGATGCCCTTGTCAGAATCTTACATGCTAGCATGAGACGATGTTGAAGGTGGTTTTAATTTCATCATTGATGCCAAGGCACGGCCATAATTTGGTTGGATCTTGTAGAATGATAAGATGTGATTCATACGTTTTGTACCTTGTATAGCAAGTTACTGTATTACTACATCGAATTAAATTTTTGGCAAAAAAAGAGACTGGACCTTAATTCAGCAAAATGTTTGTTTTGAATTTTATTTTTTGTTTTCTAGTAAAATATGAAGATTTTCTTTTGACTTAGGAAAAAATAGTTATGCCTAGATTACAAGTAATAATATGAATTTGAGGTTTTTAATTTAAGATATTCGGTTGTAAAATATATTATTTAGTCGTGAGCGGCTAGTGTCCCGTGGACGCGTCCGCGGGTGTTTAGTGAGGCCTGGGATTTGCATGTTAGCGTTGTAGATGCTCTAACAACCTTTCAATTCAACTAAGCACCTAATACCACAACATGCATGCATCCAACGGAATCAGGAATCAGTGAACATCAACAACTGCGGTGGTGTGTGTGTCTCGGGCCGGTTATGTAGAGAGGATTGATTTCATGGAACGCTTGGGATGAATGGATGCAAAGAAAGAAAAGTTAAGAAAGATCTCCTACTAGCAAACTGTGCAAAGATCATTCTTTCGTGCCCGCACACACATGTTTGTCTTTTTCAATGAATTCGAAGCTTTATTCAACGAAGCAGTAACCGGTTACAACCAGACCGTAGGCAACTGATAAGGAAATCGGGGCTGTGGTCTAGCCAACTTTCAATCCCATCTAGTGAGCACGCACATTGGGCACACTCGTGAGCCGAAACATTTAAGTTCCTTACTAACATGCCGAGGAAAACAAAACTAGGTATAATAGCCCCTAATTCTCTTAAGATAGACGTCACAATTGATTTTGTGGTGATGTGCGAATTCCAAAGGTCGATCAACTCGAAGCAATCAGCTTGTATTATCACATGTATCTCCACAGCTGAGAAAAATCACTCCCTCCCATAACGCCAGCAGTTCAGAGACAAAAGGATTTGAAACTCCCGGTAGTGGCTTGCACCAGGCTGCCAAGAATGCCAGGTGCGAGTGAGCTACTCCTCCTGCTCCCGCTGAGGGGGCATCCTAGAGCTACCGATCCATCGGCATTGATAGTGATCCATCCCACCTCCGGTGAATACCAGGATTGTGCTGAAGTAGAACTTACTCGCCGTGTCGGAAGCTTGAGGAGTGCTAGGGTTTCATGCACCCATTTGATGGCCAGAACCGGGTCATACCCATCTTTATCATGAGTCCATATACTACGAGAAGACCAAATGGCATGCATGATAATTATGATCTTGCATCTAATAGCATCTGCAGAGAATTGCTGGTCCAGCAAAAATGTCACGCGCCCAAGTGTGTGGTCGGAGCCTAGGTAGATTCAGCTCGAACCAGTCTTTAGCGGCAATCCAAAACTTCTTAGCATGTGTACAAGAGATGAGCGCATGCATAAGAGTATCATCCATGGCCTGACATATGTCACACCCACCAATCGGTTTAATATGGCCGATACTTCAGGGTCATTTAGTCTGGTAGGATGCCATGAAGAACCCTCCAACCAGAACACTCAAACCTTTGGTACAACATGTAGTTTCCATAGTTCCATAACTGTTTGTTTGAAGATGAAGTGTCCGTGATCATCCCTTCCTCTAGGTAAGCCCATTGCGAGTCGCGAGGAGGACGATACGCCCGATTTAACAGTATAATGACCCAACTTTTCCAGGCCCATGCCAAAGTATCATCATCCCCAGCCGGATTCAAAGGTATATTCAAAATCGCCTCCGCATCTGGGTGAAGAAAGTTTTGTCGGTCCAGATCCACATCCCAACTGTATAAGCATCAATCAAATCTGAAACCCTTTCCAAAGGTGCGTTACCAATCCATTATGGGTTTCAACGTTGCTGTATTTTGGATCCCATGGTCTGTCCATATAGAAACAGAGGAGCCGTCTTCAATTCTGTGAATAAGTGCATTATCCAAAGCTTTTCTGCCAAGCACAGCGCTCTCCAGGTAGATGATGCTCGACTGGGGATCGTTGCATGCAGAAATCAGTATATGGGTAGTACTTTCCCTTCAGTACACGCGCACATAAACTGTTGGGGGTGTGTCAACAACTGCCAGTCCTGTTTGCCTAACATGGCAAGATTAAATAGTTCAGGGCCCTAAAACCCATCCCTCCTTTGATCTTCGACTCAGTCAATTTCTTACGATAGACAATGAAGTGCTCTCTTGTAAATGGAGTACCCCACCAATATTTGGCCATTGGTGAGGTGATTTTGCAAACTTATTTTGTCAACAAGAAACAACTCATAATGTATGTCGGTATAGCCTCGGCAACAGATTTCAGCAAAACTATCTTAGTCACACAGCTAGGTTCCTCACCGATAACCACATATTTTGCCATGCACGCTCAATAATAAGAGAGAATGTGCCGTTGGTGATTCTTCCAATTGCAGTAGGTAGCTCCAGGTAGCTGTTTTCTGAAAACGCTTCCACCGAATCCCAAAGAGCAGATTTTAATGTCCCTTTTAAGGGCTGACCTGTCTTACTGAATTAAGAATGAACTTTTTTCTCGGTTCACAGCCAGTTCAGACGATCCTCTGTGATGGAAGTGTGTTGACTCTAGGGTTTATACAGTGATTTGGTTGGATCTTGTAGATGATAAGAGGATTTGTATATCTTGTATCTTGTATAGCAAGTTACCTTGTTACTACAATCATCAAATTAAACTCGGTAAACAAAAAGGACTGGACCTTAATTCAGAAAAATGTTAAATTTCAATTTTGTTTTCAAGTGTATTATAGAAAAAATTGTGGCAAAAGTATTGTAGATTTTTATTTTGACTTAAGAAAAAGAGTTATAACTGATGAGGCCACCAAGAGGATAAAATCATTAGTGATGGTGAAAACTGAGAGTAAGAGATAAGTAAATATGGCATCCGACTAGAAAACCACTCGGCAGCAGAACAAGATTTTTACCATAAGTAAGACTTGCGCATCATCAGATGAGAAAACCACGTAGAAACTCAATAGCAACACGGTATTTTCCTTTCGAACACACGTACATTCGACTAAGGCTCTAACAGCAAATTTTGGATGAAGCAGGCTACAACAATCAGTAGCCTTACAGATGGGAGCTCATGTGCTCAACACGCGAACCAACCTGTTGATGGACAGGACCAAGAAGCCCAGCCGCATAGAAGTTGTTGGTTGTAGAGGCTCATCCCCACGGAACGCTACGGAGGGCATCTATGCTAGGTTGGTGAGGTTTTGCAACAGCACATTGGCGACTAGCCTACCCAGATCGTGCTTTGTCATTCCGCTGCTTCCCAGTGGCTCCATTGGCCTTGGTTGATGAGGACGGCGACTGCGGTGGCGTGTGTCTCGGGCTGGTTATGGGGTGTTCATATGGAACATATATATGAGACGAATGGATGCAAACAAGCAAAAAAGGTAAGAAGATCCTACTAACAAACTGCAAGATCATTTCTTTGGTGCCAAGGCGCGCCAGTGATTTGGTTTCTTTGAACGAGCGCGCTAGTGAGTTGGTTGGATCTTGTGCAATGATAAGATGGGATCTGCCTTGTATCTTGTACTGTACCTTACCATTATACCGTATGGTTAGATCAAAATGGAGAGAGCAAGAGTTTAGAGTTGCTTCGTTTTGATTCGGCATCAGAATGCATGGTTCGAGCTATGTACGCGATTGGCATCAGGCTTGTTCTTCCTTTATGCTTTAGACGAGTTAGAAGTATATACTCGCCGGTCGTCAATTAGCAATACATG

>ONT4

ACAAGCGGCCGCTTCGTTCAGTTACGTATTGCTAACAGATTAGTTCCAACATGTGTTGCTAAGCCAAAGCCCCGGTGCCAAGAGCGGGAGGTGCTGTTCAGGGCCGCCTTATTGGAGAATTTTAGAGGGTCTTGGAGAGCTGGTACGGTTGAAGAGGAGATGACAGCTCGGTTGAAGATCTTCGTAAGGCGGTGCTCGGTTACGGTGTTGCTGAAGCCACCGTTGATCATAACAATCCAGTATATAGTGTGTTTCTATAAAGGCTCTATGCTTGGCTAGGCCATTGCAGAAGACCGCGTCGGCACATGGCCCTGGAGGACGATGAGTTCGATCTCGGGCTTGGCGTACGCCCTGGCTATGTGATGTTAATTTGTGAAGCGACACGCCCTAGCTATGTGATATTGAATTTCGTTCTTTCCATGCGCTTAGCGCGCGTAATCAAGTGGAGGTAGGTTAATTACCGCCTTGAGACACATTCAGGGCCGGCCCATAGGCAGGGGCAACGTGGAAATTCTGCCGGTTTTATCATCACACTGATATTATTGTAGTACTATAGATTATATGATGGTTTAAACGGATCTTCGTGTCCCTCGGTCACATGTGCATCCTCTCTGGGAGTCCCGGGCGAACACGGACCACGGCGCGGTCGAAATCATATAAATCGTCCTAACTGAAAAGGATGAAAACGGTGGGTAACGGTCGGTAAAATGCCTCTACCGTTTTCATTTCCATATTTAACTTGCGGGACGGAAACGAAAACGGGATATGCCGGTAACGAAAACGAACGGGATAAATACGGTAATCGAAAACGATACGATCAGTCGGGTTAAAAAAGTCGAAATCCGGAATGAGACGGTATTTTGTTCGGTAAAATCACACATGAAAACATATATTCAAAACTTAAAAACAAATATAAAAAATTGTAAACACGCTCCATGGGAATTCGTAAGGAGCTGCAGTGCAACGTGACCGGTCGTGCCTCTCTAGAGATAATGAGCATTGCATGTCTAAGTTATAAAAAATTACCACATATTTTTTTTTGTCACACTTGTTGAAGTGCAGTTTATCTATCTTTATACATATATTAAACTTTACTCTACGAATAATATAATCTATAGTACTACAATAATATCAGTGTTTTAGAGAATCATATAAATGAACAGTTAGACATGGTCTAAGGACAATTGAGTGACTGACAACAGAGCTCTACAGTTTTATCTTTTTAGTGTGCATGTGTTCTCCTTTTTTTTTGCAAATAGCTTCACCTATATAATACTTCATCCATTTTATTAGTACATCCATTTAGGGTTTAGGGTTAATGGTTTTTATAGACTAATTTTTTTAGTACATCTATTTTATTCTATTTTAGCCTCTAAATTAAGAAAACTAAAGCTCTATTTTAGTTTTTTTATTTAATAATTTAGATATAAAATAGAATAAAATAAAATCAAACTAAAAATTAAACAAATACCCTTTAAGAAATTAAAAAAACTAAGGAAACATTTTCTTGTTTCGAGTAAGATAATACCGGCTGTTAAACGCCGTCGACGAGTCTAACGGACACCAACCAGCGAACCAGCAGCGTCGCGTCGGGCCAAGCGAAGCGGGCGGCCTGCCGAGCATCATCGCTGCCTGCGGACCCCCTCTCGAGTTCCGCTCCACCGTTGGACTTGCTCCTTTGTCGGCATCCAGAAATTGCGTGGCGGAGCGGCAGACGTGAGCCAGCACGGCAGGCGGCCTCCTCCTCCTCCCGGCACGGCAGCTACGGGGGATTCCTTTCCCACCGCTCCTTCGCTTTCCCTTCCTCGCCGCCGTAATAAATAGACACCCCCCTCCACACCCTCTTTCCCCAACCTCGTGTGTTCGAGAGCGCACACACACAACCAGATTTCCCAAATCCCACACACAACCAGATTTCCCCCAAATCCACCGTCGAGCACCTCCGCTTCAAGGTACGCCGCTCGTCCTCCCTCTCTACCTTCTCCTAGATCGGCGTTCCGGTCCATGGTTAGAACCCGGTAGTTCTACTTCTGTTCATGTTTGTGTTAGATCCGTGTTTGTGTTAGATCCGTGCTGCTAGCGTTCGTACACGGATGCGACACCGCGTACGTCGAGACACGTTCTGATTGCTAACTTGCCGGTGTTTCTCTTTGGGAATCACGGATAGCTCACGCCGTTCCGCGAGACGGGATCGATTTCATGATTTTTTTGTTTCGTTGCATAGGGTTTGGTTTGCCCTTTTCCTTTATTTCAATATATGCCGTGCACTTGTTTGTCGGGTCATCTTTTCATGCTTTTTTGTCTTGGTTGTGATGATGTGGTCTGGTTGGGCGGTACGTTCTGGATCGGGTAAATTCTGTTTCAAACTTCACAGTGGATTTATTAATTTTGGATGTTTGTATGTGTGTGCCATACATATTCATAGTTACAGGTGAAGATGATGGATGGAAATATCGATCTAGGATAGGTATACATGTTGATGCGGGTTTTGCGAGTGCATATACGGAGATGCTTTTTGTTCGCTTGGTTGTGATGATGTGGTGTGGTTGGGCGGTCGTTCATTCGTTCTAGATCGGAGTAGAATACTGTTTCAAACTATTTTGGTGTATTTATTAATTTTGGAACTGTATGTGTGTGTCATACATCTTCATAGTTACGAGTTTAAGATGGATGGAAATATCGATCTAGGATAGGTATACATGTTGATGTAGGTTTACTTGATGCATATACATGATGGCATATGCAGCATCTATTCATATGCTCTAACCTTGAGTACCTATCTATTATAATAAACAAGTATGTTTTATAATTATTTTAGATCTTGATATACTTGGATGATGGCATATGCAACAGCTATATGTGGATTTTTAGCCACACCTTCATACGCTATTTATTTGCTTGAAATCTTTGTTTCTTTTGTCGATGCTCACCCTGTTGTTTGGTGTTACTTCTGAGGTCGACTCTAGAGGATCCCGGGTAGTCGAGTCCCTTCCATCACTACGAATCTTGGAGCGCCTTGCCCTCTTTGATCGGTTGCGTTTCCCGATCTGCCAGGACTTTGTCCCAGGATCCTAAACGACCGCAGATATTACTTTTATTCGAAATAGATCTTCTCTCGAAACTTATGACATGCTTCCCCCACATGCTCTCTACCAACGGTGGATTCAACCAAGACTTTGTACTCCCGAAGAACCACTTGTTTGCTTCGACTTTGACTTCGCGAACGGCTATCGAAAATTATATAACCACATCGTCGGGACTAATTGAATGGTGCCAAGCCGGCTTATCGCCCTTCCCACCTTCACAAAGCCAAGCAAATGCCCATTCCCTAGTTGCAAGTCGGCCACGGTCTTCCGAAAGCGGACGGGACTTTAGGCGGCATTGCCGGCAACACTTGACGCTTTTGTCGCTACTCGGGAATGCCCTCGATCCAAGCTCAAGACACCGCAAGAAGTCGGCACCAAAGGCTTTGCGACTCGCAAGGACCGTGCTCGGCATGAGTCTAAGCACAAACCAACGATGCGGTGCCCTTGGCAAGACAAGGAAGGACAACAATGTCGAGGGTCTTTAGCGGGGGTGGATAACATGGAGGGCTGTGTGCGGGGAGAGAACCATCTTTTTCAAAGATGGCCAGACTTCTAGACCATGGCGAAGTCAAGTTCGAAGGTGACACCTGGTGAATAAAGTGAAAGTGAAGGAACGCTGGCTTTAAGGAGGATGGAAACATTCTCGTACAAAAATTTGGAATACAACTATAACTCCCCCGATGTGTACATCATAGTGACGAGCAAGAATGGCATCAAGGTCAACTTCAATTGGACACAACATTGAGGATGGCCCCATGTTATCCACCACCTTAAAGACCTCGGACATTGTTCCAATCCTTCTTATCTTGCCAAGGGAGCCTTCCTCCTTGTTAGTTTGTGCTTAGACTCATGCCGAGCACGGTCCTTGCGAGGTCGCAAAGCCTTTGGTGCCGACTTCTTGCGGAGTCTTAGGCGGCCCCGAGGGCATTCGCCGGGTAGCGGCCAGAAGCAGCAGTGTTCCGTATGCCGCCTAAGAGTCCGTCAAGCTTTCAAAAGGCGTAGCCGACTTGCAACTAGGGAATGGAGCATTTGCTTGGCCGTGTGAAGGTGGGAAGGGCGATGGGCGGCAACGCTCGTCCACGGTCCGACGATGTGGTTATATAATTTTCGATAGCGGGTTCGCGAAGTCAAAGTCGAAGCAAACAAGTGGTTCTTCGGAGTACAAGTCTTGGATTTGAATCCGTTGGTAGAGAATTTATGTGAGTTATCAAAGTCCGCATAAGTTAGAAAATCTGGCTGGGTGTGGTGATTCATAATTAGGCCAAGTCCGGGACAAGAATCACTTGAGGTCGAGAAGCTTTAACTCGATCAAAAAGAGGGGAGCAAGCCTCCTCAAAATTTAGTGATGGGAGGACGGCTCGAGATCCTGCGTCGACTGCGGCACCGTCGGCACTCAGTAAAAAGCATTCTGTAGCCAAACTCAAATAGCCGTATAGAAAACGAGGCAAAAATCACATACCTTGCTACTGCTCAACATATCAAGTATATCAAGATCAAAATAATTATAAAACATACTTGACTATTATAATAGATAGGTACTCAGGACAGATGCAACAGATATATGTGCATATGGCCATCATTCACATCAACATGTGCTCTATCCTGAACGACAACATCATCACAAACCGTGCTATGATGTGGCACATCTGATTCAAAAGTAATAAATGCCAGTGATTTGACAGCCTATCGATCTGACAGAACGGACTTCAAACTCCCCATCATCTGACAACGTGAGGCTCTATGCATGACAAAACCAAATCAACATGTACCTATCTGAATCAATTAACAAATATCATCTTCAGTAATAACTATAATTAATAATAGCACTACATACGGGTAAAATTAACTCATTACCAGTGGTTGAAACAAATTCCTCAGTCTAGAACGACCTTAATGAACCACATCATCACAACCAAGACAAAAAAGCATGTGACACATTTCTAGTGGTCGACGTATCAGAATGAGAGGGCAAGCAAACTTATCTGGCAACAAATCATGAAATCGATCCCGTCTAGCCGAACGACTGCATCCGTTCCCAAGGAAACAGCGGCTCTGAGACAACGATCAGAACGTGTACGACGTACGAACCATCAAGAATGCGGCAGCAACGCGGATCTAACAACACGGATCTAACACAAGCAAGACAGAAGTGAAGCATGCCGGGCCTAACCATCGGAGGCGGGGCTGCCGATCTGGAAAGTGAGAGGGGAGGACGACGCTCTGCCGAGCGGGCGCGGCCGGGTGGACTGAGTCAGTTTGTGTGTGGGATTTGGGGGGAAATCTGGTTGTGTGCTGTGTGGCTTCCCGACAACACGGGTGAGAAAGGTGTGGAGGGGGTGTCTATTTTATTACGGCGGCCCGAGAGGGGCGAGGACGGTGGGGAAAGGAATCCGTGTACCGTGCCGTGAGAGGAGGGAGGGAGACACCTGCCGATGCTCGGCTCACGTCTGCCGCTCCACCACGCAGGCCCCTCTGGATGCCGGCCTGTGAGGGCGGGTCCGCGGTGGGCGGGAGGGAGTCCGGGCGCGACGAGATGCCGTGCCGCTCTGCCAAGCCGGCCGGCGCGACGCTACTGAGCCCAGGGTGGTGTGCGTTAGACTCGTCATTGCACCCGGCTGGCGGTGTTTTAACGAGCTGGCATTATCTACTCGGGGCTACTTAGTTTTTTTTAATTTCTTAAAGGAGTATTTGTTTAATTTTTAATTAAAACATTTTATTCTATTTTATATCTGATTATTAAATAAAAAACTAAAATAGAGTTTTTAGTTTTCTTAATTTAAATTTAAAATAGAATAAAATGGATGTACTAAAAAATTAGTCTATAAAAACCATTAACCCTAAACCTAGATGAATGTACTAATAAAATGGTGAAAGTATGTACCAAGGTGAAGTATTTTACAAAGAGCTGTACATAAGCAAAACTGTAAGTCCTGTTGTCAAATACTCAATTGTCCTTTAGACCATGTCTAACTGTTCATTTATATAATTCTCCAACCACTAAATATTATTGGTATATAGTTATATTATTCGTAGAGTGAGTTTAAATATATGTATAAAGATAGATAAACTGCTTCAAACAAGTGTGACAAAAAATATGTGGTAATTTTTTATAACTTAGACATGCAATGCTCATTATCTCTAGAGAAGGGCACGATAGGGATGCATCTGCCACGCAGCTCCTTACGAATTCCCATGGACGTGTTTACAATTTTTTTTATATTTGTTTTTAAGTTTTGAATATATGTTTTCATGTGTGATTTTACCGACAAAAATACCGGTTCCCGTCGATTTCGACTTTAACCCGACCGGATCGTATCGGTTTTCGATTACCGTATTTATCCCGTTCGTTTTCGTTACCGGTATATCCCGTTTTCGTTTCCGTCCCGCAAGTTAAATATGAAAATGAAAACGGTAGGGGTATTTTTGCCGACCGTTACCGACCGTTTTCATCCCTAGTTAGGACGACGGTGATTTCGGCCGCGCGTCGTCGTGTTCGCCGAAGAGACTCAGTCACATGTGGCCGGGGTCACAGTGATCATTTAACTCCTCCGATCGTTAAGCTGCTGATAGTTTAAACGGATCACTTCGTGTCCCTCAGTCACATGTGTCCTCTACGGGAGTCCGGGCGAACACGGACACGGCGCGGTCGAAATCCACTCGTCGTCCTAACTAGGGATGAAAACGGTCGGTAACGGTCGGTAAAATACCTCTACGGTTTCATTTTCATATTTAACTGCGGGACGGAAACGAAAACGGGATATACATTGCGAAAACGAACGGGATAAATACGGTAATCGAAAACACGTCGATCCGGTCGGGTTAAAGTCGAAATCGGTGGAGCCGTGTTGCTGTCGGTAAAATCACACGAAAACATATATTCAAAACTTAAAAACAAATATAAAAAATTGTAAACACGCTCCATGGGAATTCGTAAGGAGCTGCAGTGCAGCAATGACCGAAGTGATGCCCTCTCTAGAGATAATGAGCATTGCATGTCTAGTTATAAAAAATTACCACATATTTTTTGTCACACTTGTTTGAAGTGCAGTTTATCTATCTTTATATATATTTAAACTTTACTCTACGAATAATATAATCTATAGTACTACAATAATATCAGTGTTTTAGAGAATCATATAAATGAACAGTTAGACATGGTCTAAAGGACAATTGAGTATTTTGACAACAGGACTCTACAGTTTTATCTTTTTTAGTGTGCATGTGTTCTCCTTTTTTGCAAATAGCTTCACCTATATAATACTTCATCCATTTTATTGATACATTCATTTAGGGTTTAGGGTTAATGGTTTTTATAGACTAATTTTTTAGTACATCTATTTTAGTCTATTTTGTATCTAATTAGAAAACTAAAATATATTTAGTTTTTTTATTTAATAATTTAGATATGAAATGAATAAAATAAAGTGATCGACTAAATTAAACAAATACCCTTTAAGAAATTAAAAAAACTAAGGAAACATTTTTTTCTTGTTTGAGTAGATCTAATACCCGTATAATCGCAGCTCGGCTGAGTCTAACGGACACCAACCAGCGAACCAGCAGCGTCGCGTCGGGCCAAGCGAAGCAGACGGCACGGCATCTGCAATGGCTGCCTGCGGACCCTCTCGAGAATTCCGCTCCACCCGTTGGACTTGCTCCGCGTCGGCCTCCGAAGTGCGGCGGGCAGCGGACGTGAGCCGGCACAGCAGGCGGCCTCCTCCTCCTCTCACGGCACGGCAGCTACGGGGGTTGCCCACATCTACACGCTTCCTTCCTCGCCCCGCCGTAATAAATAGACACCCCCTCCACACCCTCTTTCCCCAACCTCGTGTTGTTCGGAGCGCACACACACACAACCAGATTTCCCCCCGTCCCACACACAACCAGATTTTCCCCCAAATCCACCCGTCCGGCACCTCCGCTTCAAGGTACGCCCGCTCGTCCTCCCCCCTCTCTACCTTCTCTAGATCGGCGTTCGGTCCATGGTTAGGAACCCGGTGTTCTCTTCTGTTCATGTTTGTTGAATCCGTGTTTGTGGTGGAGTGCTGCTAGCGTTCGTACACGGATGCGACCTGTACGTCGGACGTTCTGATTGCTAACTTGCCAGTGTTTCTCTTTGGGGAATCTGGGATGGCTCTAACCGTTCCGCGAACGGGATCGATTTCATGATTTTTTTTGTTTCGTTGCATAGGGTTTGGTTTGCCCTTTTCTTTATTTCAATATATGCCGTGCACTTGTTTATGGAGTCCATCTTTATGCTTTTTTTGTCTTGGTTGTGATGATGTAGTGCAGGTTGGTAGTTCTAGATCGGAGTAGGCATTCTGTTTCAAACTACACAGTGGTTGTGTTTTGGATGCATGTATGTGTGTGCCATGCATATTCATGGTCTGATGAAGATGATGGATGGAGAATATCGATCTAGGATAGGTATACATGTTGATGCGGGTTTTACTGATGCATATACGGAGATGCTTTTTTTGTTCGCTTGGTTGTGATGTGGTGTGGTTGGCGTTGATTATTGATCCTAAGTCGGAGTAGAATATTTGTTTCGAAACTTCTGGTGTATTTAGCTAATTTTGTTTGTATGTGTGTGTCATACATCTTCATAGTTACGAGTTTAAGATGGATGAAATATCGATCTAGGATAGGTATACATGTTGATGTGGGTTTGTGCGGTACCTTGCTGATGGCATATGCGGCATCTATTCTGTATACTCTAACCTTCAGTGCCTATATTATAATAAACAGTATGTTTATAATTATTTTTGATCTTGATATACTTGGATGATGGCATATGCGGCAGCTATGTGGTTTTTAGCCACCTTCATACGCTATTTATTTGCTTAGTACGTTTCTTTTGTCGATGCTCACACTGTTGTTTAGTGTCGCCACGGAGTCGGCTCTAGAGGATCCCGGGTAGTCGGTCCCTTCCTTCCATCACACTCACAATCCGGAGCGCCTTGCCCCTCCTTTGGTCAGTTGCGTTTCCCGATCTGCCAAGGACTTTGTCCCAGTCTAAACGACTATGAATCACCAACATTCGAAATAGATCTTCTCTCGAGAAACTTATGACTTTGACAACCCCCATACTCTCTACCAACGTGGATTCAACCAAGACTTTTATTACTCCAGAAAGAACCACTTGTTTAGCCGACTTTGACCATGAACCCAGCTCCTCGAAAATTATATAACCACATCGTGGTACATTGGGCGCGGTCTCGGCGGCTACTCGCGCACACACACGGCCAAGCAATGCCCATTCCTAGTTGCAGTCGTGATCTTTGAAAGCGGACGGGACTTTGGGCGGCATTACCGGCAGCACTTCAAGCGCTTTTACCATTGCCGAATGCACCAATTCTGACTCAAGACACGCAGAAGTCGGCACCAAAGGCTTTGCGACTCGCAAGGACCGTGCTCGGCATGAGTCTAAGCTGCGCTTAACGATGCGGTTGCCCTTGGCAAGACAAGGAAGGACAACAATGTACGAGGGTCTTTAGCGAGAATGGATAGCATGGGAGGGCTATGTGCGGAGAGAACCATCTTTTTCAAAGATGACGGGAACTACAAGACCCGCTGGGGAGTCAAGTTCGAAGGTGACACCCTGGTGAATAGAATCGAAATTTGAAGGGCATTGACTTCAGGAGGATGGAAACATTACAGCCCACAGTGGAATACCATTATACTTCCCACAATGTGTACATCATGGCCGACAAGCAAAGAATGGCATCAGGTCCAACTTCAAGATGGACACAACATTGAGGTTGTGCAGCCATCACCACCTTGCGAAGACCCTCGGACATTGGTGTCCTTCATACATGTTGCCAAGGGGCACACGCCTTTGTTGGTTTGTGCTTAGACTCATGCCGAGCACGGTCCTTGCGGTCGCAAAGCCCTTTGGTGAGCTTCTTGCGGGTCTTTGGCTGGGCGAGGGCATTCGAGTAGCGACGGAAAAGCGCTTGGTGTTGCCGGTAGCTCTTGTCCTAAAGTCCCGTGCGCTTTCAAAGGCGTGGCAAGAGCTGCAGCAGGGAATGGGCATTTGCTTGGCCGTGTGAAGGTGGGAAGGGCGATAGGCCCGGCCTTCTTGCGTCCAACGGGTCCGACGATGTGGTTATATAATTTTTCGATAGCCGGGTTCGCGAAGTCAAAATGAAACAACCAGAGTGGTTCTTGGGTACAAAGTCTTGGTTGATCCCACCGTTGGTAGAGTATGTGAGGAGGTTATCCAAAGTCATAGAAGTTTACGAGAGAAGATCTATTTCGAATGTTGGTGATTCATAGTCGTTTAGATGCGGGACAAGTCCTTGGCGAGGATGTCGGGGGAAGCAACTCGATCAAAGAGGGGCAAGGCGCTCAGGAATTCATGATGAGCTTGTCAAACAGATCAGTTGTTTGGCAATAAAGTTTCTTAAGATTGAATCCTGTTGCCGGTCTTGCGATGATTATCATATAATTTCTGTTGAATTACATTAAGCATGTAATAATTAACATGTAATGCATGACGTTATTTATGAGATGGGTTTTTATGATTAGAGTCCCGCAATTATACATTTGTACGCGATAGAAAACAAAATATAGCGCGCAAACTAGGATAAATTATGTACTTGCGCAGTGTCATCTGTTACTTTATAGATCGACGCTGAGCTCGAATTCTAGTGGAGACCATATTGCAGTCATCCCGGAGTGCGAAAACACGGTAACGAAACGGGATCATCCCGATTAAAAACGGGATCGATGAAACGGTCGGGAAACTAGCTCTACCGTTTCCGTTTCCGTTTACCGTTTTGTATCCCGTTTCCGTTCCGTTTTCGTTTTACCTCGGGTTCGAAATCGATCGGGATAAAACTAACAAAATCGGTTATACGATAACGGTCGGTACGGGATTTTCCCATCCTACTTTCATCCACAGTCCTAACGTCGGAAGCCAGTTTCATCATCTAGCTTCTCCTTGAACCACATGGGCGCTAAGCTTGATTCTTGCGGTGGAGACAAGGGGAAGCCAGCGCGCCACCCACGTCAGCAAATACGGAGGCGCGGGGTTGACGGCGTCCACCCGGTCCTAACGGCGACCAACAAACCAGCCAGAAGAAATTACAGTAAAAAAAGTAAATTGCGCTGATCCACCTTTTATTACCTAAGTCTCAATTTGGATCACCCTTAAACCTATCTTTTCAATTTGGGCCGGATTGGTTTGGACTACCATGAACAACTTTTCATCCGCCTGTCTAACTTCCCTTTCAGCAAACATATGAACCATATATAAGGAGATCGGCCGTATACTAGAGCTGATGTGTTTAGGTCGTTGATTGCACGAGAAAAAAAAATCCAAATCGCAACAATAGCAAATTTATCTGGTTCAAAGTGAAAAGATATGTTTAAGGTAGTCAAAGTAAAACTTATAGATAATAAAATGTGGTCCGAAAACGTAATTCACTCAAAAAATCAACGTGATGTGTACCAAACGGAGACAAACGGCATCTTCTCGAAATTTCCCAACCGCTCGCTCGCCCGCCTCGTATTCCCGGAAACCGCGGTGGTTTCAGCGTGGCGGATTCTCCAAGCAGACGGTGATGTCACGGCACGGGACTCCTCCACCACCCAACCGCCATAAATACCAGCCCCCTCATCTCCTCTCCTCGCATCAGCTCCACCCCCGAAAAATTTCTCCCCAATCTCGCAGAGCTCTCGTCGTCGAATCGAATCCTCTCGCGTCCTCAAGGTACGCTGCTTCTCCTCTCCTCGCTTCGTTTCGATTCGATTTCGGACGGGTGAGGTTGTTTTTGTTGCTAGATCCGATTGGTGGTTAGGGTTGTCGATGTGATTATCGTGAGATGTTTGGGGTTGTAGATCGTGGTTATGATTTGGGCTTTGATTTGGTTCGATAGGTGGAATCGTGGTTAGGTTTTGGGATTGGATGTTGGTTCTGATGATTGGGGGAATTTTTACGGTTAGATGAATTGTTGGATGATTCGATTGGGAAATCGGTGTAGATCTGTTGGGGAATTGTGGAACTAGTCATGCCTGAGTGATTGGTGCGATTTGTAGCGTGTTCCATCTTGTAGGCCTTGTTGCGAGCATGTTCAGATCTACTGTTCCGCTCTTGATTGAGTTATTGGTGCCTTGGGTTGGTGCAAACACAGGCTTTAATATGTTATATCTGTTTTGTGTTTGATGTAGATCTGTAGGGTAGTTCTTCTTAGACATGGTTCAATTATGCGGCTTGTGCGTTTCGATTTGATTTCATATGTTCACAGATTAGATAATGATGAACTCTTTTAATTAATTGTCAATGGTAAATAGGAAGTCTTGTCGCTATCTGTCATAATGATCTCATGTTACTATCTGCCAGTAATTTATGCTAAGAACTATATTAGAATATCATGTTACAATCTGTAGTAATATCATGTTACAATCTGTAGTTCATCTATATAATCTATTGTGGTATTTCTTTTACTATCTGTGTGAAGATTATTGCCACTAGTTCATTCTACTTATTTCTGAAGTTCAGGATACGTGTGCTGTTACTACCTATCTTTGAATACATGTGTGATGTGCCTGTTACTATCTTTTTGAATACATGTATGTTCTGTTGGAATATGTTTGCTGTTTGATCCGTTGTTGTGTCCTTAATCTTGTGCTAGTTCTTACCCTATCTGTTTGGTGATTATTTCTTGCAGATGCAGATCTTTGTCAAAACATTGACCGGCAAGACTATCACCCTGGAGGTGGAGGGAGTCCTCTGACACCATCGACAATGTCAAGGCTAAGATCCGAATAAGGAGGGCATCCCCCCGGACCCAGCGGCGATTGATCTTCGCTGGCAAGCAGCTCGAATGGCGGGACCCTTGCTGACTACAACATCCAGAGGAGTCCACCCTTCACCTTGTCCTCCGCCTCCGTGGTGGAATGAAAAGCCTGAACTCACCGCGACGTCTGTCGAAGTTTCACGATCGAAAGGTTCGACAGCGTGTCCGACCTGATGCAGCTCTCGGAGGGCGAAGAATCTCGTGCTTTCAGCTTCGATGTAGGAGGGCGTGGATATGTCCTGCGGGTAAATAGCTGCGCCGATGGTTTCTACAAAGATCGTTATGTTTATCGGCACTTTGCATCGGCCGCGCTCCCGATTCCGGAAGTGCTTGACATTGGGGAGTTTAGCGAGAGCCTGACCCTGGCCATCTCCCGCCGTTCACCAGAGGGTGTCACGTTGCAAGACCACCTGAAACCGAACTGCCCGCTGTTCTACAACCGGTCGCGGAGGCTATGGATGCGATCGCTGCGGCCGATCTTAGCCAGACGAGCAGGTTCCCGGCCCATTCGGACCGCAAGGAATCGGTCAATACACTACATGGCGTGATTTAATACGCGCCGCGATTGCTGATCCCCATGTGTATCACTGGCAAACTGTGATGGACGACACCGTCAGTGCGTCCGTCGCGCAGGCTCTCGATGAGCTGATGCTTTGGGCCAGGGACTGCCCCGAAGTCCGGCACCTCGTGCACGCGGATTTCGGCTCCAACAATGTCCTGACGGACAATGGCCGCATAACAGCAGTCATTGACTGGAGCGAGGCGATGTTCGGGGATTCCCAATACGAGGTCGCCAACATCTTCTTCTGGAAGCGTGGTTGGCTTGTATGGAGCAGCAGACGCGCTACTTCGACGGAGAGCATCCGGAGCTTGCAGGATCGCCACGACTCCGGGCGTATATGCTCCGCATTGGTCTTGACCAACTCTATCAGAGCTTGGTTGACGGCAATTTCGATGATGCAGCTTGGGCGCAGGGTCGATGCGACGCAATCGTCCGATCCGGAGTAGGGACTGCCGGTACACAAATCGCCCGCGAAGCCGCGGCCGTCTGGACCGGTAGACTGTGTAGAAGTACTCGCCGATAGTGGAAACCGACGCCCCAGCACTCGTCCGAGGGCAAAGAAATAGGCTTTCAGCCAGTTTGGTGGAGCTGCCGATGTGCCTGGTCGTCCCGAGCCTCTGTTCGTCAAGTATTTGTGGTGCTGATGTCTACTTGTGTCTGGTTTAATGGACCATCGAGTCCGTATGATATGTTAGTTTTATGAAACGGTTTTCCACCAATGGGACATGGTATGCTTTATGAATAAGTTGGATTTGAACCTAAATATGTGCTCAATTTGCTCATTTTGCATCATTCCTGTTGATGTTTTATCTGAGTTGCAAGTTTGAAAATGCTGCATATTCTTATTAAATCGTCATTTACTTTTATCTTAATGAGCTTTGCAATGGCCTATGGGATATAAAAGATTATTCTGGGAAGTGATGCTGGAAGGACTATGCTGTCCTGATTTATATTTGGAGCCACTATGAGCATTTGGGCTTTCTTTTCAGAACGCTGTAGGCGTGTGTTGAAATCTTTGCGACATTCAATTTGATATATGATTCGAGGTAATTGGGCTTTAATTTGTCATCTCATGTAACATCTTTTTGTTTTCTTCGCTCGCTTGATTTCTATATATTTAGTAAGCAGCATTGGAAGATAATAATGTGAATGATGATATACTCCAATACTTGCAATTTCAAAACCGTTAGAAAGAAAGGAAAATCACGGCCGCTGGATCCGAATTCGGCACAGAAGTGTTCAACCCCAGTACATTAAAAACGTCCGCAATGTGTTATTAAGTTGTCTAAGCGTCAATTTGTTTACCACAATATATCCCAGGTAGTATTACATATATGTATTAGTATATTTTTATTTAGATCCAGAAAAAAAAGGAAGAAGAAAAAAGGCCCAAACCCACAACGTAGGTAGCTTTCCCGGATCGAAAGAATCGTTCCCTAGTTTACTATGCTGCTGCAGCACTACATCGATCTCCTACGGTCATCTCGCCTCCTCGACTCCTAAAAAATCTTGCCTCTTCGGCTCCTCCCCCAAATTCTTCAATCCTGGAAGACAGCGTAGTCGCTCCCGTTCACTTTCAAATTCGAGATGGTGCTCCCGTGCATCCACTAATACTGGAGACCGATGAAGATTTCTATTCCCCTTCCCAAATCACTTTGCTGCAGCATCCTCGGTGTTCCCCATCAATAGACGTTTGAAGCATCACCATGCCCATCAGAGAGATGGTACGTCTCCCTCCTTAGCCTAATCAAGTATTATTTTTCATTCGCTGTTAAACAACCACAACCTCTTCGCTTGATTGCTCCACACGACATCTTCAATTTTTCTCTTCTAGTTTCTACTCTTTTGTTGGCTGTTCTTCGAACCTTCATTGGATCCGGGAAAACTACATCGACAATTCGACATCAAGCCATATGCCCATACCCAAATGCTTTTGTTCCTTGTATTAACAGTTTTTCTGTAATTATTTGGTGACATGTGTTCTAAGAAATGTTTATCGTAATGAGAAAATAAAGTGAAAAAAACATAAATGGTTCAAGTTGCTATCAGTTTTGACTATGGTTTCATACTTAGAAGAGCAAGATGATAGTCTTTTTGTGCAACTTAAAAATTTCTTTTGTAGAACTATATTATTTCCATACTATTATGATGTAAGTTATACTAAATGAAAGCTGATAAATACGTATCACAGAATTACTTTAGAAATATAATATATTCTTAATTTTATTTGTATACGAATGCATTTGTCGTCTCTTCATTTGAATATGGACCGGCCCTGGGCACATTATTGTTTCTTCTGATTAGATGTGACGTTTCGTGTGTGTGCGTGCGTGCGTATATGTGTGTGTGGGTGTGGGTGTGTAGGGGGGGGGGGGGGGGGGGCTTAGCTAGGCCAGGCTATAATCACATGCCCGATGCTAGAATACTTGTGGCTAAGAATAAGCTGCACCGGGAGATTTCCTACAGGTGGAGGAGCAAGGAGCCGATGGAGAGCTTGGTGAAGAACCAAGCATGTGCGCTGGAGCTCTGGCGAAGTGCTCGACGAAGAACCGCAGAGCTATGACCCAAAACCATTGTTTTAAATAGCCGGCTATAGTCCTCTATAGCCTTGCTATACTTTTTAATAAGTAGTGCGGATTAAATGTGTGCGTATAAAATAGAAGCCAATATATGACGCTTTAGCTCCGCTATAGGCGGTTAGAGGCCCGCCGTTATTGTGTAGCCGCTATTTAAAACATTACCCAAAACCCAAGCCCACGAAAAAATTCAACGCCCCTATCAAGCACGGATGAGCTCGTAAAGGTATGGTCTTGTTGTTGAAGTACCAAGTGACCACCATGATTCTGCTGCTGTTGTACCCAGCAAATTAGAAGGGAAAGTGTTGAACAAAATCCAACATTTGAGCTTGTCTGCATCCAGAGAAGCGGGCATGAGAAGATGCCCACTGAACACCATGATGCTTGTGCTGTTGTACCGTGTTAGGTCACAGTTGGACCCCTATACTTTTAGCAGAGAACACACACCTCATCAGGATGAAAGTTCCAACGGATACCCTTGTCAGAATCTTACATACTAGCATGAGACGATGTTGACCACA
